# Enantio- and regioselective asymmetric allylic substitution using a chiral aminophosphinite ruthenium complex: an experimental and theoretical investigation†

**DOI:** 10.1039/d1ra06824e

**Published:** 2021-12-09

**Authors:** Rajesh K. Jena, Dhiraj Das

**Affiliations:** Centre of Excellence in Advanced Materials and Applications, Utkal University, Vani Vihar Bhubaneswar-751004 India rajeshjena@utkaluniversity.ac.in; Department of Chemistry, Indian Institute of Technology Kharagpur Kharagpur 721302 India; Department of Chemistry, University of Calcutta Kolkata India

## Abstract

The design and synthesis of a new chiral aminophosphinite-ligated ruthenium complex is described. The ruthenium complex, [Ru(AMP)_2_(CH_3_CN)_2_][BPh_4_]_2_ {AMP = (*S*)-*tert*-butyl 1-(diphenylphosphinooxy)-3-methylbutan-2-ylcarbamate}, has been found to catalyze nucleophilic addition of phenol and carboxylic acid to allyl chloride in a highly regioselective fashion with enantiomeric excess ranging from 12 to 90.

## Abstract 2

A new chiral –P,N-ligand, and its ruthenium(ii) complex has been synthesized, which catalyzed the highly regioselective *O*-allylation of phenols and carboxylic acids to afford the branched product as the major product with high to low enantioselectivities.

## Graphical abstract

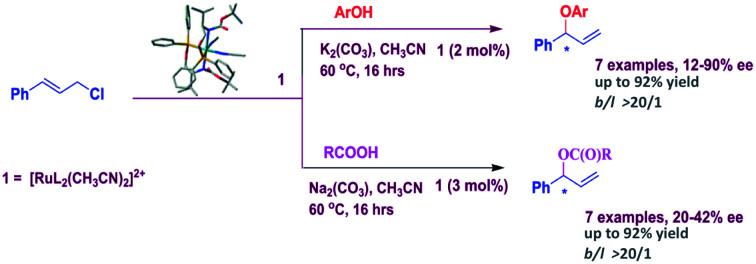

## Introduction

Transition metals containing P,N-ligands have been used in homogeneous catalytic processes, and the bidentate ligand coordination has been found to improve, in some cases, the catalytic activity.^1^ The bidentate ligands with soft P and hard N-donor sites impart some typical features. Due to these features, transition metal complexes containing such ligands are used as pre-catalysts.^2^ It has been found that P,N-ligands can coordinate to the metal center *via η*^1^-P or *η*^2^-P, N.^3^ These coordination modes can change during the turnover of the precatalyst. For example, the behavior of aminophosphine as hemilabile ligands, with the change of coordination to metal from *η*^2^-P, N to *η*^1^- P during the catalysis allows the dangling nitrogen atom to act as a “proton messenger” in the catalytic process.^4^

Optically active allylic aryl ethers are used as precursors for the synthesis of biologically active organic molecules^5,6^ and their derivatives are valuable building blocks for organic synthesis.^7^ An efficient approach to these compounds involves transition-metal-catalyzed allylic substitution with oxygen nucleophiles.^8–11^ Transition metal catalysts have been used for stereospecific allylation of phenol.^12^ The enantioselective version of the reaction has been reported by using chiral palladium complexes.

Aryl ethers are common subunits of biologically active molecules. Apart from their use as precursors for the Claisen rearrangement,^13,14^ aryl allyl ethers are not used extensively as building blocks for natural product synthesis because methods for their enantioselective syntheses are limited. Ruthenium^12*a*^ and rhodium^12*b,c*^ catalyzed stereospecific synthesis of the allylic ether of branched carbonates were reported, and a few enantioselective palladium-catalyzed examples have also been reported.^15,16^ Elegant applications of the palladium-catalyzed chemistry for the synthesis of natural products demonstrate the potential of asymmetric allylic etherification in organic synthesis.^17^ Thus, new and more general regioselective methods for the construction of allylic ethers would be synthetically valuable.

**Scheme 1:**
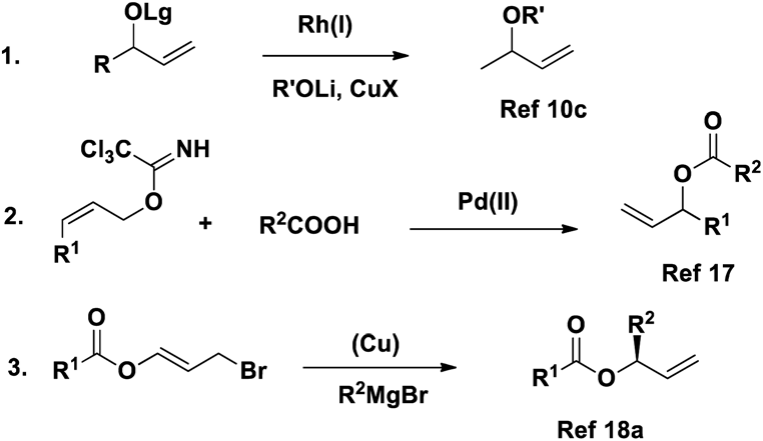
Few reported reactions of metal catalysed allylic substitution.

Although asymmetric allylic substitution with carboxylates seems to be a fascinating direct process, only a few reports are available,^18^ probably because of the high reactivity of the resulting allylic esters with metal catalyst (Scheme 1). Overman disclosed the Pd-catalyzed asymmetric synthesis of chiral allylic esters from (*Z*)-allylic trichloroacetimidates and carboxylic acid.^19^ However, this system does not apply to the *E* isomer, and the relatively large leaving group is unfavorable from the viewpoint of the atom economy. Feringa and co-workers offered another route to allylic esters *via* Cu-catalyzed asymmetric allylic alkylation of 3-bromopropenyl esters,^20^ but the use of a Grignard reagent led to some limitations.

We have been involved in the synthesis and structure of ruthenium(ii) complexes and studies of their catalytic properties towards, carbon–carbon and carbon–heteroatom bond formation.^21^ In continuation of our endeavor we thought to design and synthesize a chiral –P,N-donor ligand and synthesis its ruthenium complex and study the catalytic properties of the complex for asymmetric synthesis.

**Scheme 2:**
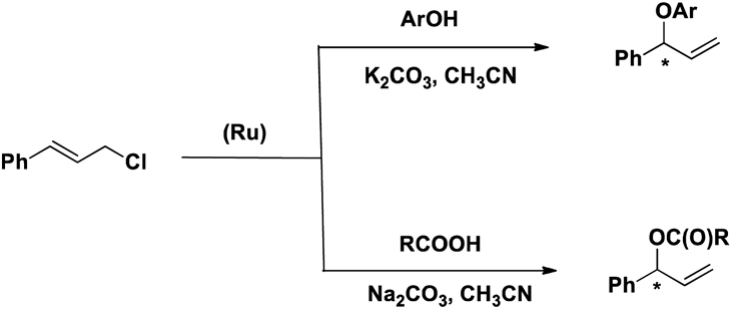
Reactions reported here.

Herein we describe the synthesis and characterization of a new chiral ligand, (*S*)-*tert*-butyl 1-(diphenylphosphinooxy)-3-methyl butan-2-ylcarbamate (L) and its ruthenium complex, [RuL_2_(CH_3_CN)_2_][BPh_4_]_2_ (1). The efficacy of 1 as a catalyst for the regioselective and enantioselective addition of phenols and carboxylic acids to allylic chlorides has been determined (Scheme 2).

## Results and discussion

### Synthesis and characterization of (*S*)-*tert*-butyl 1-(diphenylphosphinooxy)-3-methylbutan-2-ylcarbamate (L)

The ligand, (*S*)-*tert*-butyl 1-(diphenylphosphinooxy)-3-methylbutan-2-ylcarbamate (L) has been synthesized from the reaction of BOC-protected l-valinol with PPh_2_Cl in the presence of Et_3_N in toluene. The ligand was found to be stable in the solid-state. However, it was found to be very susceptible to oxidation in solution when exposed to air. The compound was characterized by high-resolution mass spectrometry (HRMS) and ^1^H, ^13^C, and ^31^P NMR spectroscopy.

The ^31^P{^1^H} NMR spectrum of L in CDCl_3_ shows a singlet at 115.6 ppm, which is similar to that reported for other aminophosphine ligands.^22^ The ^1^H NMR spectrum of the ligand shows a multiplet at 0.93 ppm due to the methyl proton of the isopropyl group and a singlet at 1.45 ppm due to the methyl protons of the tertbutyl group. The signal for the –CH proton of the isopropyl group appears at 2.18 ppm as a broad singlet. The signal for the –CH proton adjacent to the –OC(O)NH group appears at 3.65 ppm as a broad signal and the signal for the –CH_2_ protons appears at 4.02 ppm as a multiplet. The signals for the aromatic protons appear as multiplets in the range of 7.27 to 7.86 ppm. The HRMS shows the molecular ion peak {[M + H]^+^} at 388.2033. The circular dichroism (CD) spectrum of the ligand shows three peaks at 270 nm, 260 nm, and at 250 nm with negative Cotton effect and at 240 nm with positive Cotton effect.

### Synthesis and characterization of [RuL_2_(CH_3_CN)_2_](BPh_4_)_2_ (1)

Complex 1 was synthesized from the reaction of L with *cis*-Ru(DMSO)_2_Cl_2_ in toluene in the presence of NaBPh_4_. The complex has been characterized by HRMS, IR, and ^1^H, and ^31^P NMR spectroscopy. The HRMS (ESI^+^) of 1 shows a peak at *m*/*z* 479.1774, which corresponds to the molecular ion [M^2+^]. The ^31^P{^1^H} NMR spectrum of 1 shows a singlet at 128.5 ppm. The significant shift of the ^31^P signal from that of the free ligand clearly shows that the ligand is bonded to the metal center through the phosphorus atom. The ^1^H NMR spectrum of 1 shows a multiplet at 0.93 ppm due to the methyl proton of the isopropyl group. The signal for the methyl protons of the tertbutyl group appears at 1.44 ppm. The signal for the –CH proton of the isopropyl group appears at 1.98 ppm as a broad singlet. The signal for the –CH proton adjacent to the –OC(O)NH group appears at 3.66 ppm as a broad signal and the signal for the –CH_2_ protons appears at 4.02 ppm as multiplet. The signals for the aromatic protons appear as multiplet in the range 6.87 to 7.86 ppm. In addition to these signals, a singlet appears at 2.01 ppm, which is due to the uncoordinated acetonitrile. The CD spectrum of the complex shows three peaks at 270 nm, 265 nm, 260 nm, and at 250 nm with negative Cotton effect and at 245 nm with positive Cotton effect.

**Fig. 1:**
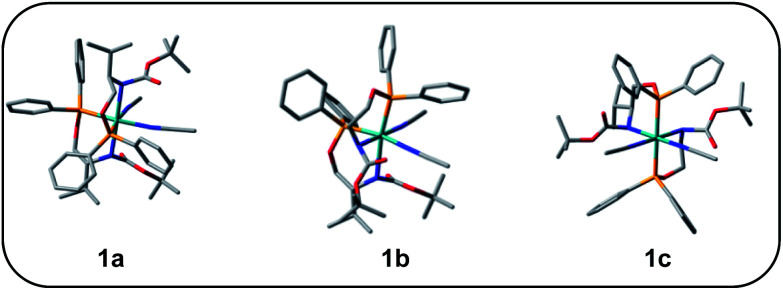
Possible optimized geometries of 1.

Unfortunately, we are unable to get a suitable crystal for the determination of the single-crystal X-ray structure. The structure of the complex was optimized using Gaussian 09 program.^23^ To begin with; we started with three possible geometries of the complex (Fig. 1). Calculation shows that 1a (total energy = −3315.45772645 a.u.) is more stable by 117.92 unit and 117.94 unit from 1b (total energy = −3197.53594805 a.u.) and 1c (total energy = −3197.51898051 a.u.), respectively. Thus from the energy minimization, it can be concluded that the solid-state structure of the compound is the same as that of 1a.

### Studies of the catalytic activity of 1

**Scheme 3:**
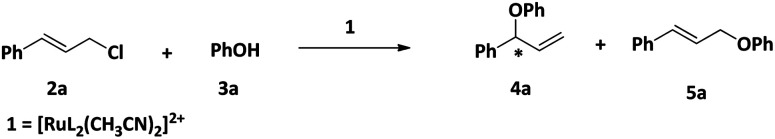
Reaction between phenol and cinnamyl chloride in the presence of 1 and base.

One of our major goals was to design a catalyst of regioselective and enantioselective addition of phenol and carboxylic acids to allylic substrates. To begin with we chose the reaction between cinnamyl chloride (2a) and phenol (3a) in the presence of 1 and different bases (Scheme 3). We found that the reaction proceeded well in the presence of 1.5 mmol of K_2_CO_3_ with very good regioselectivity (entry 4, Table 1).

**Table 1:** Effect of base on the product^a^

Entry	Base (mmol)	Conversion^b^ (%)	B/L 4a/5a^b^
1	Na_2_CO_3_ (1)	52	75/25
2	NaHCO_3_ (1)	35	—
3	K_2_CO_3_ (1)	85	90/10
4	K_2_CO_3_ (1.5)	92	98/2

a Reaction condition: 2a: 2 mmol, 3a: 1 mmol, 1: 2 mol%, solvent: CH_3_CN, time: 16 h, temp: 60 °C.

b From ^1^H NMR.

We then carried out the reaction in various solvents (Table 2) and the ideal solvent was found to be acetonitrile.

**Table 2:** Solvent optimization^a^

Entry	Solvent	Conversion^b^ (%)
1	Toluene	—
2	CH_3_CN	92
3	THF	60

a Reaction condition: 2a: 2 mmol, 3a: 1 mmol, 1: 2 mol%, K_2_CO_3_: 1.5 mmol, solvent: CH_3_CN, time: 16 h, temp: 60 °C.

b From ^1^H NMR.

The temperature and time variation studies show that the reaction precedes best at 60 °C for 16 hours with very good regioselectivity (entry 5, Table 3). Finally, the ideal concentration of 2a, 3a, and 1 was found to be 2 mmol, 1 mmol, and 2 mol% respectively (entry 3, Table 4).

**Table 3:** Effect of temperature on the conversion^a^

Entry	Temp. (°C)	Time (hrs)	Conversion^b^ (%)	4a/5a^b^
1	RT	12	—	—
2	RT	16	Trace	—
3	40	16	48	80/20
4	50	16	75	85/15
5	60	16	92	98/2
6	75	16	89	98/2
7	90	16	87	96/4
8	60	24	90	98/2

a Reaction condition: 2a: 2 mmol, 3a: 1 mmol, 1: 2 mol%, K_2_CO_3_: 1.5 mmol, solvent: CH_3_CN, time: 16 h, temp: 60 °C.

b From ^1^H NMR.

**Table 4:** Optimization of reactants and catalyst concentration^a^

Entry	2a (mmol)	3a (mmol)	1 mol%	Conversion^b^	B/L 4a/5a^b^
1	1	1	1	55	80/20
2	1	1	2	75	82/18
3	2	1	2	92	98/2
4	2	1	1	82	92/8

a Reaction condition: K_2_CO_3_: 1.5 mmol, solvent: CH_3_CN, time: 16 h, temp: 60 °C.

b From ^1^H NMR.

We then proceeded to study the effect of leaving group on the reaction between phenol and different cinnamyl derivatives and chlorine was found to be the best leaving group (entry 1, Table 5). Interestingly the regioselectivity was found to be opposite when allyl acetate was used (entry 4, Table 5).

**Table 5:** Effect of leaving group on conversion of the product^a^

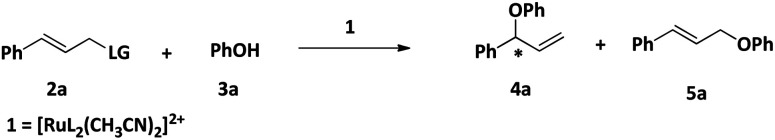			
Entry	Leaving group (2)	Conversion^b^ (%)	B/L^b^
1	Cl (2a)	92	98/2
2	Br (2b)	80	60/40
3	OH (2c)	—	—
4	OAc (2d)	92	2/98

a Reaction condition: 2: 2 mmol, 3a: 1 mmol, 1: 2 mol%, solvent: CH_3_CN, time: 16 h, temp: 60 °C.

b From ^1^H NMR.

After optimizing the reaction condition, we studied the scope of the reaction. The results are summarized in Table 6. The reactions of cinnamyl chloride (2a) with various derivatives of phenol selectively produced the corresponding branched ethers 4 in good yields with very good to low enantioselectivities. The low enantiomeric excess could be due to the higher reaction temperature.

**Table 6:** Reaction phenols with cinnamyl chloride (2a) in the presence of 1^a^

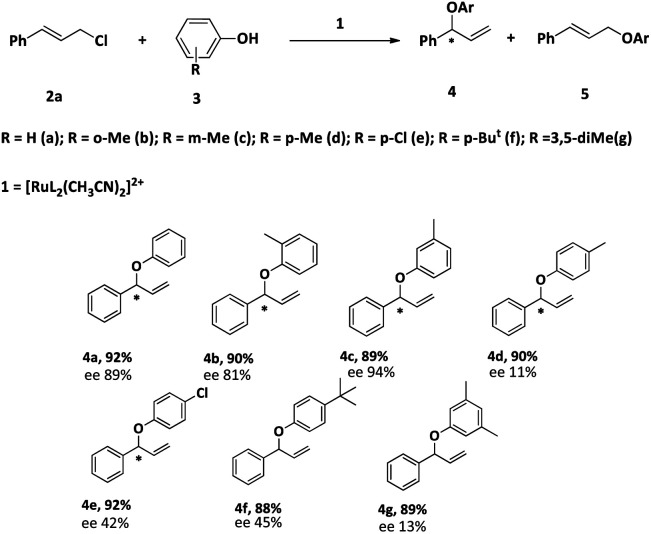

a Reaction condition: 2a: 2 mmol, 3: 1 mmol, 1: 2 mol%, solvent: CH_3_CN, time: 16 h, temp: 60 °C. Yield: isolated.

Having explored the reaction of phenols with cinnamyl chloride, we thought to explore the reaction of another nucleophile, carboxylate, with cinnamyl chloride. The reaction between cinnamyl chloride (2a) and benzoic acid (6a) was chosen as a model reaction for optimization studies. The best conversion was achieved when Na_2_CO_3_ was used.

Similar to the reaction between 2a and 3a, acetonitrile was found to be the ideal solvent for the reaction between 2a and 6a. The best conversion was achieved when the reaction was carried out in the presence of 3 mol% of 1 for 24 hours at 60 °C.

After optimizing the reaction conditions, we studied the scope of the reaction. The conversion and the regioselectivity were found to be very good (Table 7). However, the enantiomeric excess was found to be very poor.

**Table 7:** Reaction carboxylic acids with cinnamyl chloride (2a) in the presence of 1^a^

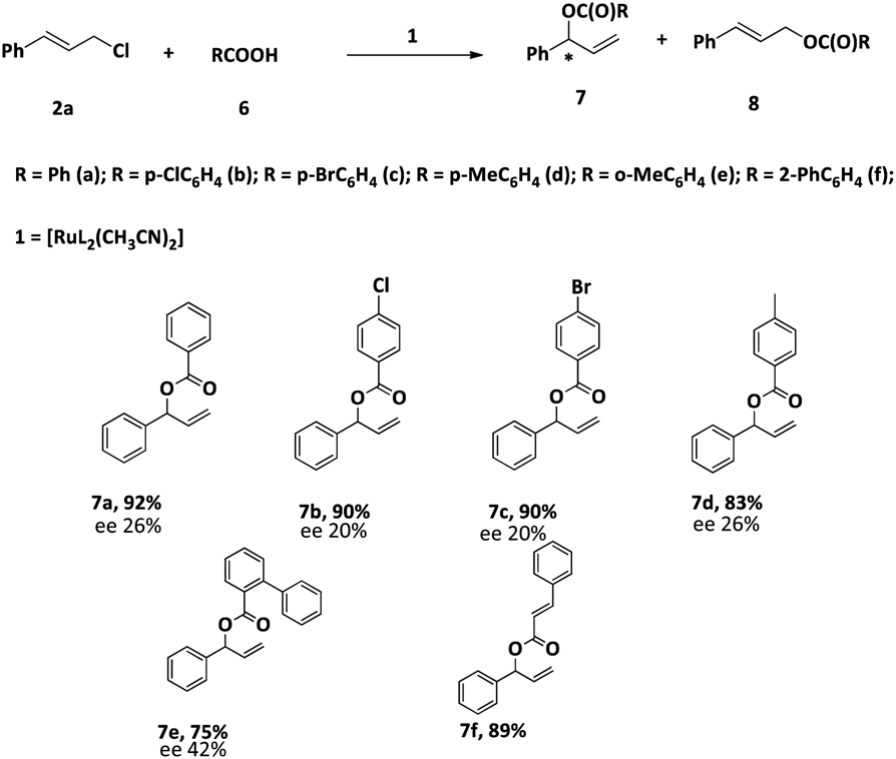

a Reaction condition: 2a: 1 mmol, 6: 1 mmol, 1: 3 mol%, solvent: CH_3_CN, time: 24 h, temp: 60 °C. Yield: isolated.

All the products have been characterized by ^1^H and ^13^C NMR spectroscopy and two new compounds 4f and 4g are further characterized by HRMS.

### Mechanistic investigation

Onitsuka *et al.* have reported regio- and enantioselective *O*-allylation of phenol, alcohol, and carboxylates catalyzed by a chiral cyclopentadienyl ruthenium(ii) complex where they have isolated the *η*^3^-allyl intermediate and structurally characterized by single-crystal X-ray diffraction study and explained the mechanism based on the intermediate.^24^

To obtain information on the reaction mechanism, we examined the stoichiometric reactions of 1 with 2a in CD_3_CN by ^31^P and ^1^H NMR spectroscopy. Thus, we treated 1 with 2a (1 : 1) in CD_3_CN and after heating for a certain time; we recorded the ^1^H NMR as well as ^31^P NMR (Fig. S39 and S40, ESI†). In the ^1^H NMR spectrum, we found a new doublet at *δ* = 5.07 ppm. This signal is the characteristic peak of the *η*^3^-allyl system.^25^ In the ^31^P NMR spectrum, we found two singlets at 142.2 ppm and 115.0 ppm. The peak at 115.0 ppm is due to the free ligand (*vide infra*). The peak at 142.2 ppm can be assigned the intermediate [RuL(CH_3_CN)(*η*^3^-allyl)Cl]^2+^. So from *in situ* NMR studies, it is clear that in the presence of reactant, one chiral ligand is dissociated from the metal center.

**Fig. 2:**
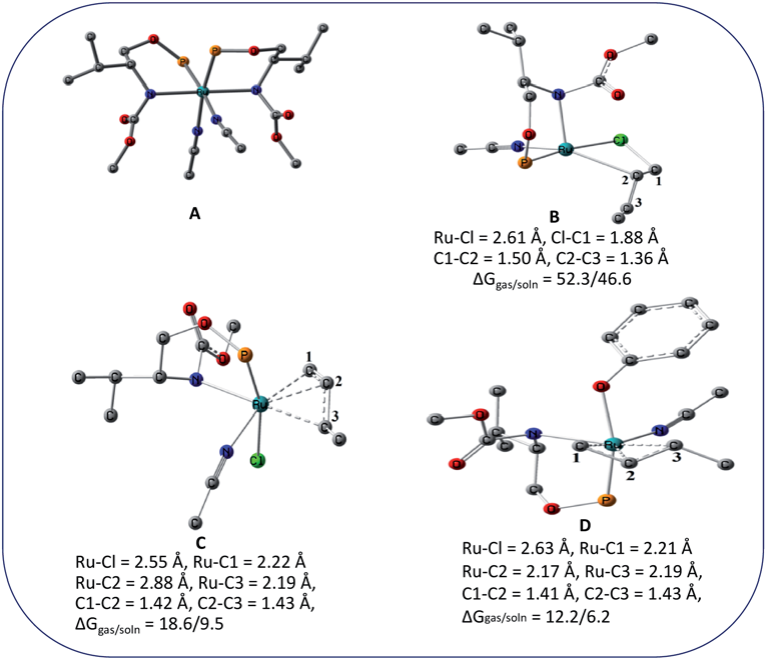
Geometries of the intermediates for the enantioselective allylic nucleophilic substitution. Hydrogen atoms are not shown for clarity.

**Fig. 3:**
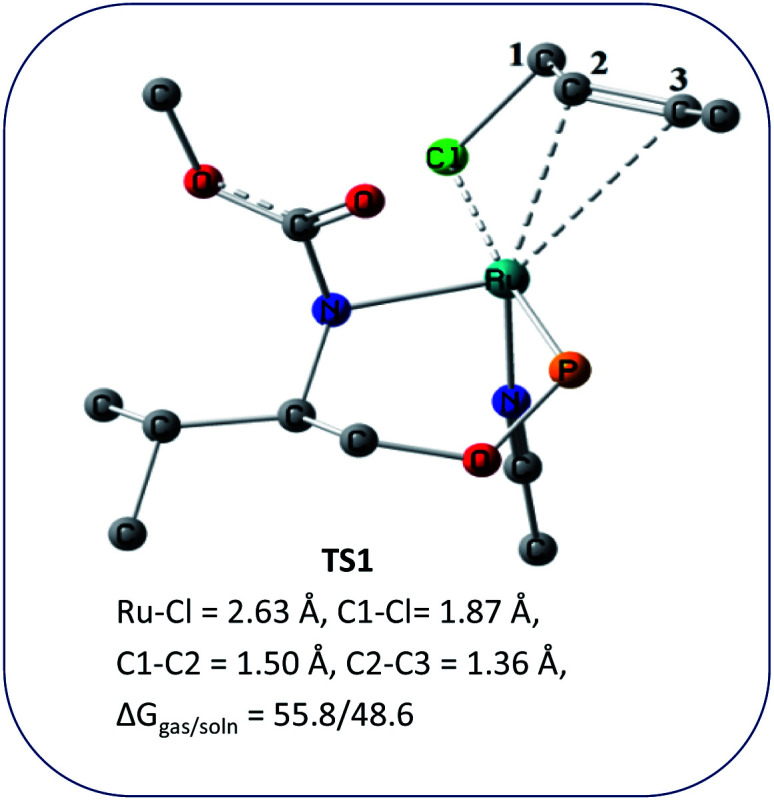
Geometries of the transition state, TS1 for the enantioselective allylic nucleophilic substitution. Hydrogen atoms are not shown for clarity.

**Fig. 4:**
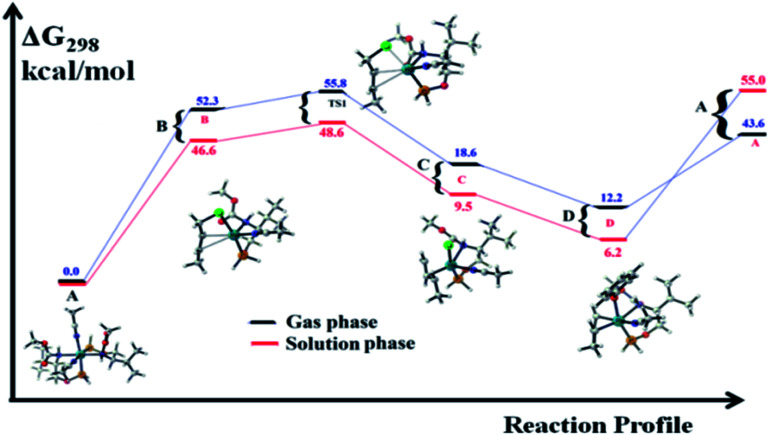
Free energy profiles for the regioselective *O*-allylation of cinnamyl chloride catalysed by 1. The values presented in blue color (dark black line) are Δ*G*_298_ in the gas phase, and the values presented in red color (dark red line) are Δ*G*_298_ in acetonitrile.

We wanted to validate the mechanism of the reaction through theoretical investigations. Accordingly, we performed density functional theory (DFT) computations to gain some insight into the possible mechanism of allylic nucleophilic substitution catalyzed by 1. For smooth calculation, we have replaced the –Ph groups of the phosphorus atom with H atoms and the *tert*-butyl group by a methyl group. We have optimized the geometry of all the intermediates (Fig. 2) and transition states (Fig. 3). We have also computed the relative energies between the competing paths (Fig. 4). A stable minimum was found for every postulated intermediate within the catalytic cycle in terms of the Gibbs free energies (Δ*G*).

The catalytic cycle starts with octahedral ruthenium(ii) complex, A (Fig. 2) where it contains two chiral ligands and two acetonitrile molecules. In presence of reactant, it loses one acetonitrile molecule and one ligand (*vide infra*) and forms the intermediate, B (Fig. 2) where Ru–Cl bond distance is 2.61 Å. Then, oxidative addition of ally chloride to the ruthenium center affords the intermediate C (Fig. 2) where the Ru–Cl bond distance is 2.55 Å *via* the transition state TS1 (Fig. 3) with the Ru–Cl bond distance of 2.63 Å.

**Fig. 5:**
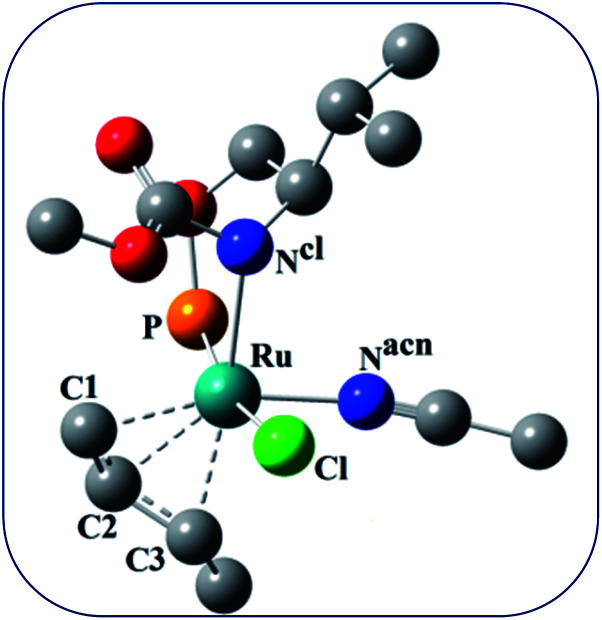
Ball and stick model of the intermediate C.

It has been observed that in the intermediate C, the –Cl atom is more close to the allylic carbon C2 (Fig. 5) than terminal one (C4). High regioselectivity and enantioselectivity of the reaction and previous report by Onitsuka *et al.*^24^ led us to think that the Cl atom of the intermediate C is substituted by the phenoxide on Ru to afford the intermediate D (Fig. 2) which is thermodynamically more stable than C by 6.4 kcal mol^−1^. Then subsequent reductive elimination leads to the generation of A and liberation of the product. The intermediate C is thermodynamically stabilized by 37.2 kcal mol^−1^ from the TS1. The C–C distances (C3–C2 = 1.43 Å, C2–C1 = 1.42 Å) in the intermediate C of the allyl group indicate *η*^3^-coordination of it.

**Scheme 4:**
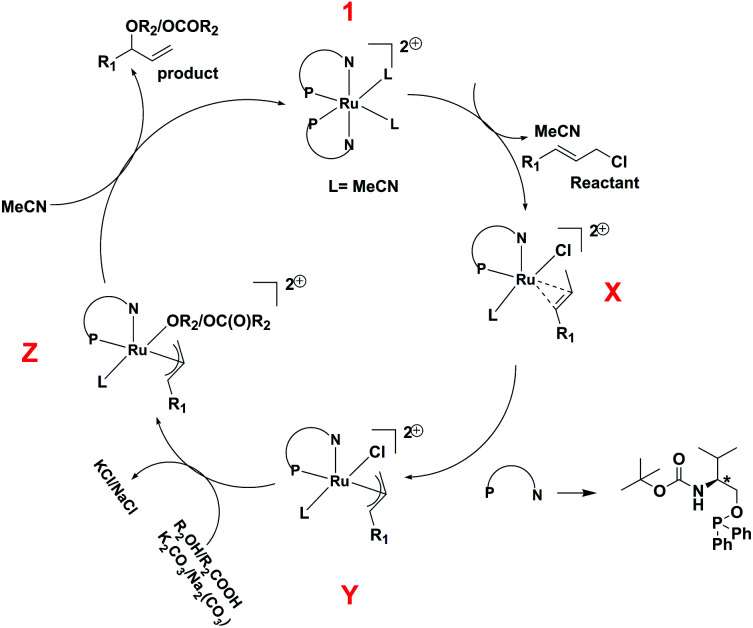
Plausible mechanism of *O*-allylation of phenol and carboxylic acid with cinnamyl chloride.

In view of the *in situ* NMR experiments, DFT studies, and the reported mechanism^25^ of the reaction we suggest a plausible mechanism of the reaction, which has been shown in Scheme 4. The starting complex, 1 loses one ligand and two coordinated acetonitrile molecules, and one allyl chloride is coordinated to the ruthenium center to give the intermediate X, which undergoes oxidative addition to afford the *η*^3^-allyl complex, Y. This is followed by the coordination of the phenol or carboxylate group to give the intermediate Z, which undergoes reductive elimination to afford the product.

## Conclusion

In conclusion, we have described here the synthesis of a new P,N donor chiral ligand and its ruthenium(ii) complex, which catalyze the highly regioselective *O*-allylation of phenols and carboxylic acids to afford the branched product as the major product with high to very low enantioselectivities. Based on the experimental and theoretical investigation a mechanistic path has been proposed.

## Experimental section

### General methods and materials

^1^H and ^13^C NMR spectroscopy were performed on 400 and 100 MHz, respectively. Chemical shifts (*δ*) are reported in ppm relative to residual solvent signals (CHCl_3_, 7.26 ppm for ^1^H NMR, CDCl_3_, and 77.2 ppm for ^13^C NMR and CH_3_CN, 1.94 ppm for ^1^H NMR, CD_3_CN). Infrared spectroscopic data were recorded on KBr plates as thin films. Solvents and reagents used were reagent-grade products. HRMS data of the newly synthesized compounds were recorded on TOF MS in ESI^+^ mode in a methanol–water mixture. Chiral HPLC analysis was performed on a Thermo Separation Products Spectra Series P-100 using Chiralcel and Chiralpak columns.

### Computational details

All the density-functional theory (DFT/B3LYP) calculations were carried out with the Gaussian 09 program.^23^ We employed LANL2DZ effective core potential (ECP) for Ru and all other atoms were treated with the 6-31G* basis set. Geometries of all species studied were fully optimized, and they were characterized as true intermediates on the potential energy surface by the absence of imaginary frequencies, after frequency calculation on the optimized geometries. Zero-point energies (ZPE) and thermal corrections at 298 K were calculated by using the frequencies computed at the same level of theory.

### Preparation of (*S*)-*tert*-butyl 1-(diphenylphosphinooxy)-3-methylbutan-2-ylcarbamate (L)

l-Valinol (60 mmol, 6.18 g) in dichloromethane (50 mL) was treated with triethylamine (66 mmol, 9.3 mL) at room temperature for 0.5 h in two neck round bottomed flask, then a solution of di-*tert*-butyl carbonate (60 mmol, 13.8 mL) in dichloromethane (30 mL) was added slowly at 0 °C. The reaction was allowed to warm to room temperature, and stirred overnight. The mixture was quenched with saturated ammonium chloride (30 mL). The aqueous phase was extracted with dichloromethane (2 × 40 mL) and the combined organic phase was washed with brine (40 mL × 2) and dried over anhydrous sodium sulfate for 0.5 h. The solvent was removed to give the Boc-protected l-valinol, which was used in the next step without further purification.

Boc-protected l-valinol (20 mmol, 4.03 g) was dissolved in toluene (25 mL) in a two-neck round bottomed flask under argon atmosphere and subsequently 30 mmol (4.23 mL) of triethylamine and then 20 mmol (3.55 mL) of chlorodiphenylphosphine were added dropwise at 0 °C. Immediately a white precipitate formed. After 1 h stirring, the reaction mixture was filtered under argon atmosphere. The filtrate was concentrated in rotavapor and upon addition of hexane to the concentrated solution a white solid was obtained. Yield 96% (7.40 g). ESI-MS [M + H]^+^*m*/*z* calculated for C_22_H_30_O_3_NP [M + H]^+^ = 388.1997 observed 388.2033. UV-visible (*λ*_max_; nm): 264. IR (*λ*_max_; cm^−1^): 3275, 298, 1700, 1530, 1365, 730. ^1^H NMR (400 MHz, CDCl_3_, ppm): 0.93 (m, 6H), 1.45 (m, 9H), 2.18 (q, 1H), 3.65 (m, 1H), 4.02 (m, 2H), 7.27–7.86 (m, 10H); ^13^C NMR (100 MHz, CDCl_3_, ppm) *δ* = 18.6, 19.5, 28.5, 29.5, 55.8, 65.7, 65.8, 79.3, 128.3, 128.5, 128.6, 128.7, 128.8, 130.5, 131.3, 131.4, 131.8, 131.9, 132.2, 132.4, 133.6, 156.0; ^31^P NMR (161.98 MHz, CDCl_3_, ppm) *δ* = 115.6 ppm.

### Synthesis of {RuL_2_(CH_3_CN)_2_][BPh_4_]_2_ (1)

In a two-neck round-bottomed flask, *cis*-Ru(DMSO)_2_Cl_2_ (2.5 mmol, 1.21 g) and L (5.12 mmol, 2.0 g) were dissolved in toluene. It was refluxed for one (1) h under argon atmosphere. The reaction solution was allowed to cool to room temperature. The solvent was removed under vacuum, and a reddish-yellow solid was isolated. Then in the second step, we dissolved the reddish yellow solid in acetonitrile and refluxed it for seven hours in the presence of NaBPh_4_ (5 mmol, 1.70 g) under argon atmosphere. The reaction mixture was filtered, and the solvent was removed under vacuum, and the yellow crystalline complex 1 was isolated. Yield 95% (2.27 g). ESI-MS [M + H]^2+^*m*/*z* calculated for C_48_H_64_N_4_O_6_P_2_Ru [M + H]^2+^ = 479.1696 observed 479.1774. UV-visible (*λ*_max_; nm): 326, 276. IR (*λ*_max_; cm^−1^): 3050, 2965, 2929, 1700, 1580, 1480, 1435, 740, 700. ^1^H NMR (400 MHz, CDCl_3_, ppm): 0.93 (m, 6H), 1.44 (m, 9H), 1.98 (q, 1H), 2.01 (s, 6H), 3.66 (m, 1H), 4.02 (m, 2H), 6.87–7.86 (m, 10H); ^31^P NMR (161.98 MHz, CDCl_3_, ppm) *δ* = 128.5.

### General procedure for the synthesis of allylic ether and allylic esters

In a Schlenk tube, a solution of cinnamyl chloride (1.0 mmol) in dry acetonitrile (1.0 mL) and 1 (2 mol%/3 mol%) were taken, and the reaction mixture was stirred at 60 °C for 20 min under argon atmosphere. After that potassium carbonate/sodium carbonate (1.5 mmol) was added, then an acetonitrile solution (1.0 mL) of phenol/carboxylic acid derivative (2.0 mmol/1.0 mmol) were added. After stirring at 60 °C for 16–24 h, the reaction mixture was diluted with diethyl ether, and the insoluble parts were filtered through Celite. The solvent was evaporated; the reaction mixture was concentrated under reduced pressure. The residue was purified by silica gel column chromatography with hexane to give a colorless oil.

#### 1-Phenyl-1-phenoxyprop-2-ene (4a)^24*a*^

Colorless liquid (Rf = 0.64); yield 92%; ^1^H NMR (CDCl_3_, 600 MHz, ppm) *δ* 7.44 (d, 2H, *J* = 7.8 Hz, Ar), 7.37 (t, 2H, *J* = 7.8 Hz, Ar), 7.33–7.25 (m, 3H, Ar), 6.98–6.93 (m, 3H, Ar), 6.10 (ddd, 1H, *J* = 16.8, 10.2, 5.4 Hz, CH=), 5.66 (d, 1H, *J* = 5.4 Hz, CH), 5.37 (d, 1H, *J* = 17.4 Hz, CH=), 5.28 (d, 1H, *J* = 10.8 Hz, CH=); ^13^C NMR (CDCl_3_, 150 MHz) *δ* 157.9, 140.1, 138.0, 129.3, 128.6, 127.8, 126.6, 121.0, 116.5, 116.2, 80.8. HPLC analysis: Chiralpak IA-3, hexane/iPrOH = 90/10 (v/v), 1.0 mL min^−1^, 254 nm; major enantiomer tR = 8.082 min, minor enantiomer tR = 7.746 min; ee 89%.

#### 1-Phenyl-1-(*o*-methylphenoxy)prop-2-ene (4b)^24*a*^

Colorless liquid (Rf = 0.65); yield 90%; ^1^H NMR (CDCl_3_, 600 MHz) *δ* 7.43 (d, 2H, *J* = 7.2 Hz, Ar), 7.37 (t, 2H, *J* = 7.2 Hz, Ar), 7.31–7.28 (m, 1H, Ar), 7.16 (dd, 1H, *J* = 7.6, 0.8 Hz, Ar), 7.07–7.02 (m, 1H, Ar), 6.86 (t, 1H, *J* = 7.4, Hz, Ar), 6.81 (d, 1H, *J* = 5.6 Hz, Ar), 6.08 (ddd, 1H, *J* = 16.8, 10.2, 5.4 Hz, CH=), 5.65 (d, 1H, *J* = 5.4 Hz, CH), 5.40 (d, 1H, *J* = 16.8 Hz, CH=), 5.26 (d, 1H, *J* = 10.8, Hz, CH=), 2.34 (s, 3H, Me); ^13^C NMR (CDCl_3_, 150 MHz) *δ* 156.0, 140.5, 138.3, 130.7, 128.6, 127.7, 127.6, 126.5, 126.4, 120.6, 116.0, 113.4, 80.6, 16.6. HPLC analysis: Chiralpak IA-3 column, iPrOH/hexane = 10%, 1.0 mL min^−1^, 254 nm; major enantiomer tR = 6.770 min, minor enantiomer tR = 5.423 min; ee 81%.

#### 1-Phenyl-1-(*m*-methylphenoxy)prop-2-ene (4c)^24*a*^

Colorless liquid (Rf = 0.65); yield 89%; ^1^H NMR (CDCl_3_, 400 MHz) *δ* 7.46 (d, 2H, *J* = 7.2 Hz, Ar), 7.41 (t, 2H, *J* = 7.2 Hz, Ar), 7.37–7.29 (m, 1H, Ar), 7.16 (dd, 1H, *J* = 7.6, 0.8 Hz, Ar), 7.07–7.02 (m, 2H, Ar), 6.76 (t, 1H, *J* = 7.4, Hz, Ar), 6.08 (ddd, 1H, *J* = 16.8, 10.2, 5.4 Hz, CH=), 5.65 (d, 1H, *J* = 5.2 Hz, CH), 5.35 (d, 1H, *J* = 16.8 Hz, CH=), 5.27 (d, 1H, *J* = 10.8, Hz, CH=), 2.32 (s, 3H, Me); ^13^C NMR (CDCl_3_, 150 MHz) *δ* 158.2, 140.5, 139.6, 138.3, 133.0, 130.7, 128.6, 127.7, 127.6, 126.5, 126.4, 120.6, 116.0, 113.4, 111.8, 80.9, 21.7. HPLC analysis: Chiralpak IA-3 column, hexane/*i*PrOH = 90/10 (v/v), 1.0 mL min^−1^, 254 nm; major enantiomer tR = 8.112 min, minor enantiomer tR = 7.776 min; ee 94%.

#### 1-Phenyl-1-(*p*-methylphenoxy)prop-2-ene (4d)^24*a*^

Colorless liquid (Rf = 0.66); yield 90%; ^1^H NMR (CDCl_3_, 600 MHz) *δ* 7.46–7.44 (m, 2H, Ar), 7.41–7.37 (m, 2H, Ar), 7.33–7.31 (m, 2H, Ar), 7.05 (d, 2H, *J* = 8.4 Hz, Ar), 6.87 (d, 2H, *J* = 8.4 Hz, Ar), 6.10 (ddd, 1H, *J* = 17.2, 10.4, 5.6 Hz, CH=), 5.63 (d, 1H, *J* = 6.0 Hz, CH), 5.36 (dt, 1H, *J* = 17.2, 1.3 Hz, CH=), 5.27 (dt, 1H, *J* = 10.4, 1.3 Hz, CH=), 2.30 (s, 3H, Me); ^13^C NMR (CDCl_3_, 150 MHz) *δ* 156.0, 140.5, 138.3, 130.7, 128.6, 127.7, 127.6, 126.5, 126.4, 125.0, 116.4, 114.9, 81.3, 20.7. HPLC analysis: Chiralpak IA-3 column, hexane/*i*PrOH = 90/10 (v/v), 1.0 mL min^−1^, 254 nm; major enantiomer tR = 6.724 min, minor enantiomer tR = 6.4528 min; ee 11%.

#### 1-Phenyl-1-(*p*-chlorophenoxy)prop-2-ene (4e)^24*a*^

Colorless liquid (Rf = 0.60); yield 92%; ^1^H NMR (CDCl_3_, 400 MHz) *δ* 7.40–7.30 (m, 5H, Ar), 7.18–7.16 (m, 2H, Ar), 6.85 (CH=), 5.52 (d, 1H, *J* = 6.0 Hz, CH), 5.28 (dt, 1H, *J* = 17.2, 1.3 Hz, CH=), 5.21 (dt, 1H, *J* = 10.5, 1.3 Hz, CH=); ^13^C NMR (CDCl_3_, 151 MHz) *δ* 156.6, 139.8, 137.8, 129.4, 128.9, 128.2, 126.8, 126.1, 117.7, 116.9, 81.5. HPLC analysis: Chiralcel OJ-H column, hexane/*i*PrOH = 997/3 (v/v), 0.8 mL min^−1^, 274 nm; major enantiomer tR = 42.1 min, minor enantiomer tR = 46.1 min; ee 42%.

#### 1-Phenyl-1-(*p-tert*-butylphenoxy)prop-2-ene (4f)^24*a*^

Colorless liquid (Rf = 0.65); yield 88%; ^1^H NMR (CDCl_3_, 400 MHz) *δ* 7.42 (d, 2H, *J* = 8.0 Hz, Ar), 7.35 (t, 2H, *J* = 7.6 Hz, Ar), 7.31–7.24 (m, 3H, Ar), 6.89–6.87 (m, 2H, Ar), 6.10 (ddd, 1H, *J* = 17.2, 10.4, 6.0 Hz, CH=), 5.63 (d, 1H, *J* = 6.0 Hz, CH), 5.33 (d, 1H, *J* = 17.2 Hz, CH=), 5.27 (d, 1H, *J* = 10.4 Hz, CH=), 1.28 (s, 9H, *t*-Bu); ^13^C NMR (CDCl_3_, 151 MHz): *δ* 155.9, 143.8, 140.6, 138.3, 128.8, 128.7, 127.9, 126.8, 126.3, 116.6, 115.7, 81.0, 34.2, 31.7, 29.9. HRMS (ESI-TOF) *m*/*z*: [C_19_H_22_O + H]^+^ calculated for 267.1704; found 267.1755. ee 45%.

#### 1-Phenyl-1-(3,5-dimethylphenoxy)prop-2-ene (4g)

Colorless liquid (Rf = 0.66); yield 89%; ^1^H NMR (CDCl_3_, 400 MHz) *δ* 7.45–7.28 (m, 5H, Ar), 6.60 (d, 2H, *J* = 4.0, Ar), 6.06 (ddd, 1H, *J* = 17.2, 10.4, 6.0 Hz, CH=), 5.63 (d, 1H, *J* = 5.6 Hz, CH), 5.34 (d, 1H, *J* = 17.2 Hz, CH=), 5.25 (d, 1H, *J* = 10.4 Hz, CH=), 2.27 (s, 6H, Me); ^13^C NMR (CDCl_3_, 151 MHz) *δ* 158.2, 140.6, 139.2, 138.5, 129.0, 128.8, 127.9, 126.8, 126.3, 123.0, 116.5, 114.1, 80.8, 29.9, 21.6. HRMS (ESI-TOF) *m*/*z*: [C_17_H_18_O + H]^+^ calculated for 239.1391; found 239.1429. HPLC analysis: Chiralpak IA-3, hexane/iPrOH = 90/10 (v/v), 1.0 mL min^−1^, 254 nm; major enantiomer tR = 6.102 min, minor enantiomer tR = 5.876 min; ee 13%.

#### 1-Phenyl-2-propenyl benzoate (7a)^24*b*^

Colorless liquid (Rf = 0.40); yield 92%; ^1^H NMR (CDCl_3_, 400 MHz) *δ* 8.13–8.11 (m, 2H, Ar), 7.55 (tt, 1H, *J* = 7.2, 1.2 Hz, Ar), 7.48–7.44 (m, 4H, Ar), 7.41–7.37 (m, 2H, Ar), 7.31 (tt, 1H, *J* = 7.2, 1.2 Hz, Ar), 6.53 (d, 1H, *J* = 5.6 Hz, CH), 6.10 (ddd, 2H, *J* = 17.2, 10.5, 5.9 Hz, CH=), 5.39 (dd, 1H, *J* = 17.2, 1.2 Hz, CH=), 5.30 (dd, 1H, *J* = 10.4, 1.3 Hz, CH=); ^13^C NMR (CDCl_3_, 100 MHz) *δ* 165.7, 139.2, 136.5, 133.3, 130.5, 129.9, 128.8, 128.6, 128.4, 127.3, 117.0, 76.9. HPLC analysis: Chiralpak IA-3, hexane/iPrOH = 90/10 (v/v), 1.0 mL min^−1^, 254 nm; major enantiomer *t* = 6.688 min, minor enantiomer *t* = 6.592 min; ee 26%.

#### 1-Phenyl-2-propenyl 4-chlorobenzoate (7b)^24*b*^

Colorless liquid (Rf = 0.36); yield 90%; ^1^H NMR (CDCl_3_, 400 MHz) *δ* 8.03 (d, 2H, *J* = 8.4 Hz, Ar), 7.46–7.31 (m, 7H, Ar), 6.50 (d, 1H, *J* = 5.6 Hz, CH), 6.10 (ddd, 2H, *J* = 17.2, 10.5, 5.9 Hz, CH=), 5.37 (dd, 1H, *J* = 17.2, 1.2 Hz, CH=), 5.30 (dd, 1H, *J* = 10.4, 1.2 Hz, CH=); ^13^C NMR (CDCl_3_, 100 MHz) *δ* 164.8, 139.7, 138.9, 136.3, 131.3, 128.9, 128.8, 128.5, 127.9, 126.3, 117.5, 77.4. HPLC analysis: Chiralcel OD-H column, hexane/*i*PrOH = 1000/1 (v/v), 0.4 mL min^−1^, 254 nm; major enantiomer *t* = 35.5 min, minor enantiomer *t* = 39.3 min; ee 20%.

#### 1-Phenyl-2-propenyl 4-bromobenzoate (7c)

Colorless liquid (Rf = 0.35); yield 90%; ^1^H NMR (CDCl_3_, 400 MHz) *δ* 7.97 (d, 2H, *J* = 8.4 Hz, Ar), 7.59 (d, 2H, *J* = 8.4 Hz, Ar), 7.47–7.32 (m, 5H, Ar), 6.52 (d, 1H, *J* = 4.8 Hz, CH), 6.10 (ddd, 2H, *J* = 17.2, 10.5, 5.9 Hz, CH=), 5.39 (dd, 1H, *J* = 17.2, 1.2 Hz, CH=), 5.32 (dd, 1H, *J* = 10.4, 1.2 Hz, CH=); ^13^C NMR (CDCl_3_, 100 MHz): *δ* 165.0, 138.9, 136.3, 132.0.3, 131.4, 129.4, 128.9, 128.6, 127.9, 127.4, 126.3, 117.5, 77.0. HPLC analysis: Chiralcel OD-H column, hexane/*i*PrOH = 1000/1 (v/v), 0.4 mL min^−1^, 254 nm; major enantiomer *t* = 22.3 min, minor enantiomer *t* = 34.6 min; ee 20%.

#### 1-Phenyl-2-propenyl 4-methylbenzoate (7d)^24*b*^

Colorless liquid (Rf = 0.42); yield 83%; ^1^H NMR (CDCl_3_, 400 MHz) *δ* 7.99 (t, 2H, *J* = 8.0 Hz, Ar), 7.48–7.30 (m, 5H, Ar), 7.25 (d, 2H, *J* = 7.8 Hz Ar), 6.52 (d, 1H, *J* = 5.6 Hz, CH), 6.10 (ddd, 2H, *J* = 16.8, 10.4, 5.6 Hz, CH=),5.39 (d, 1H, *J* = 17.2 Hz, CH=), 5.30 (d, 1H, *J* = 10.4 Hz, CH=), 2.42 (s, 3H, CH_3_); ^13^C NMR (CDCl_3_, 100 MHz) *δ* 165.5, 143.9, 139.3, 136.6, 134.3, 129.9, 129.3, 128.7, 128.2, 127.9, 127.3, 117.2, 76.7, 21.8. HPLC analysis: Chiralcel OD-H column, hexane/*i*PrOH = 1000/1 (v/v), 0.4 mL min^−1^, 254 nm; major enantiomer *t* = 17.7 min, minor enantiomer *t* = 26.7 min; ee 26%.

#### 1-Phenyl-2-propenyl 2-methylbenzoate (7e)

Colorless liquid (Rf = 0.32); yield 75%; ^1^H NMR (CDCl_3_, 400 MHz) *δ* 7.99 (t, 2H, *J* = 8.0 Hz, Ar), 7.48–7.30 (m, 5H, Ar), 7.25 (d, 2H, *J* = 7.8 Hz Ar), 6.52 (d, 1H, *J* = 5.6 Hz, CH), 6.10 (ddd, 2H, *J* = 16.8, 10.4, 5.6 Hz, CH=),5.39 (d, 1H, *J* = 17.2 Hz, CH=), 5.30 (d, 1H, *J* = 10.4 Hz, CH=), 2.64 (s, 3H, CH_3_); ^13^C NMR (CDCl_3_, 100 MHz) *δ* 167.5, 143.9, 139.3, 136.6, 134.3, 129.9, 129.3, 128.7, 128.2, 127.9, 127.3, 117.2, 76.7, 22.1.

#### 1-Phenylallyl biphenyl-2-carboxylate (7f)

Colorless liquid (Rf = 0.30); yield 89%; ^1^H NMR (CDCl_3_, 400 MHz) *δ* 7.85 (d, 1H, *J* = 7.6 Hz, Ar), 7.51 (dt, 1H, *J* = 7.2, 1.4 Hz, Ar), 7.40 (dt, 1H, *J* = 7.2, 1.3 Hz, Ar), 7.36 (dd, 1H, *J* = 7.6, 0.9 Hz, Ar), 7.31–7.26 (m, 8H, Ar), 7.12–7.10 (m, 2H, Ar), 6.27 (d, 1H, *J* = 6.0 Hz, CH), 5.80–5.74 (m, 1H, CH=), 5.14 (dt, 1H, *J* = 76.4, 1.3 Hz, CH=), 5.12 (d, 1H, *J* = 1.3 Hz, CH=); ^13^C NMR (CDCl_3_, 100 MHz) *δ* 167.7, 142.6, 141.4, 138.6, 136.1, 131.3, 131.1, 131.0, 130.1, 128.7, 128.5, 128.2, 128.1, 127.3, 127.2, 117.3, 76.8. HPLC analysis: Chiralcel OJ-H column, hexane/*i*PrOH = 100/1 (v/v), 1.0 mL min^−1^, 254 nm; major enantiomer *t* = 23.3 min, minor enantiomer *t* = 32.5 min; ee 20%.

## Conflicts of interest

There are no conflicts to declare.

## Supplementary Material

RA-011-D1RA06824E-s001
