# Supplementary material for: Enantio- and regioselective asymmetric allylic substitution using a chiral aminophosphinite ruthenium complex: an experimental and theoretical investigation
Source: RSC Adv. 2021 Dec 9;11(62):39319–27. doi: 10.1039/d1ra06824e (PMC9044493; doi:10.1039/d1ra06824e)
Supplement: RA-011-D1RA06824E-s001 [file RA-011-D1RA06824E-s001.pdf]

Electronic Supplementary Material (ESI) for RSC Advances.
This journal is © The Royal Society of Chemistry 2021

Supporting information

Enantio- and Regioselective Asymmetric Allylic Substitution by a Chiral Aminophosphinite Ruthenium Complex: An Experimental and Theoretical Investigation

Rajesh K. Jena*[a] and Dhiraj Das[b, c]

[a]Centre of Advanced Materials and Applications, Utkal University, Vani Vihar, BBSR- 751 004, India

[b] Department of Chemistry, Indian Institute of Technology, Kharagpur 721 302, India

[c] Department of Chemistry, University of Calcutta, Kolkata, India

rajeshjena@utkaluniversity.ac.in

Table of contents

Contents **Page number**

^{1}H, ^{31}P NMR spectra, HRMS, IR and CD spectra of L and 1 (Fig S1 – S10)........ 2-6

^{1}H and ^{13}C NMR spectra of **4a** to **4g** (Fig S11 – S24)........7-13

^{1}H and ^{13}C NMR spectra of **7a** to **7f** (Fig S25 – S36)........14-19

HRMS of **4f** and **4g** (Fig S37 & S38)........20

In-situ ^{1}H & ^{31}P NMR spectra of the reaction solution (Fig S39 & S40)........21

Chiral HPLC data of **4a**, **4b**, **4c**, **4d**, **4g** and **7a** (Fig S41 – S46)........22-26

Cartesian coordinates of all optimized geometries........27

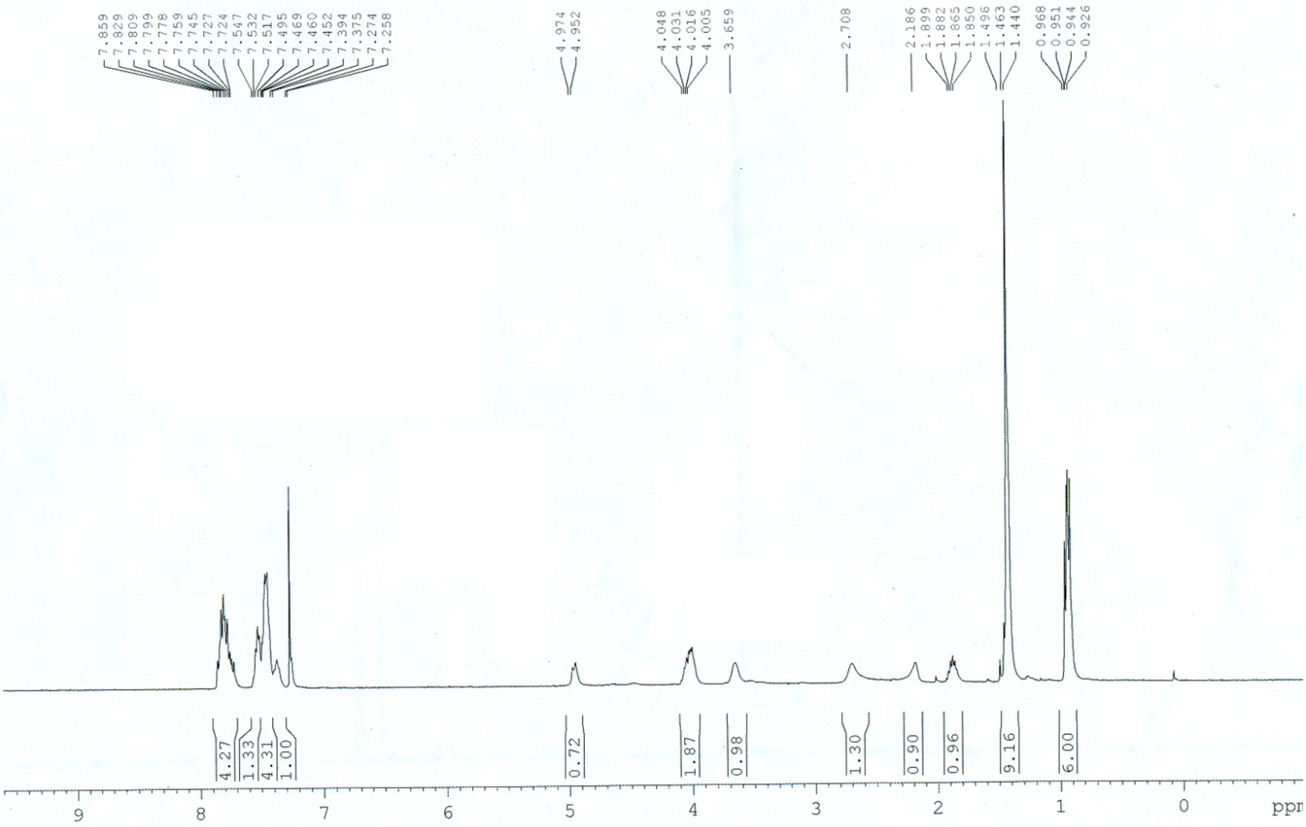

Figure S1 ^{1}H NMR (400 MHz) spectrum of L in $CDCl_3$

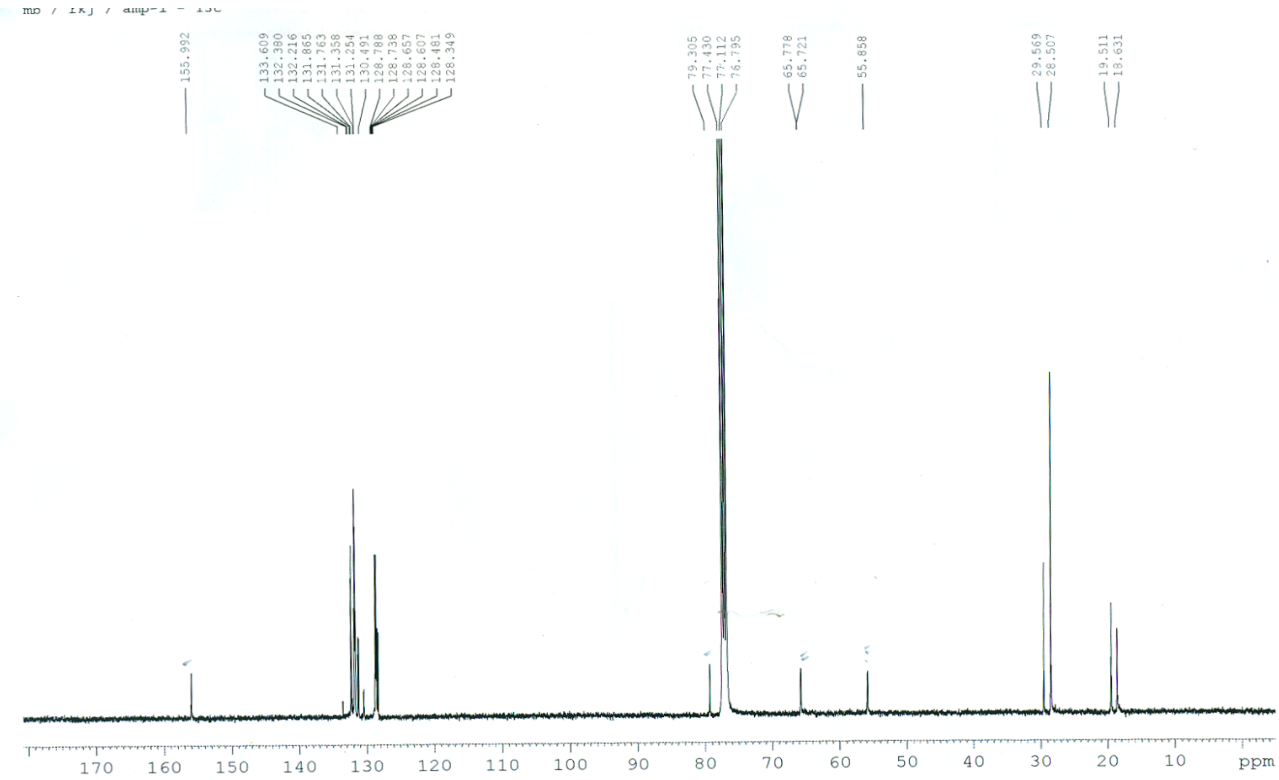

Figure S2 ^{13}C NMR (100 MHz) spectrum of L in $CDCl_3$

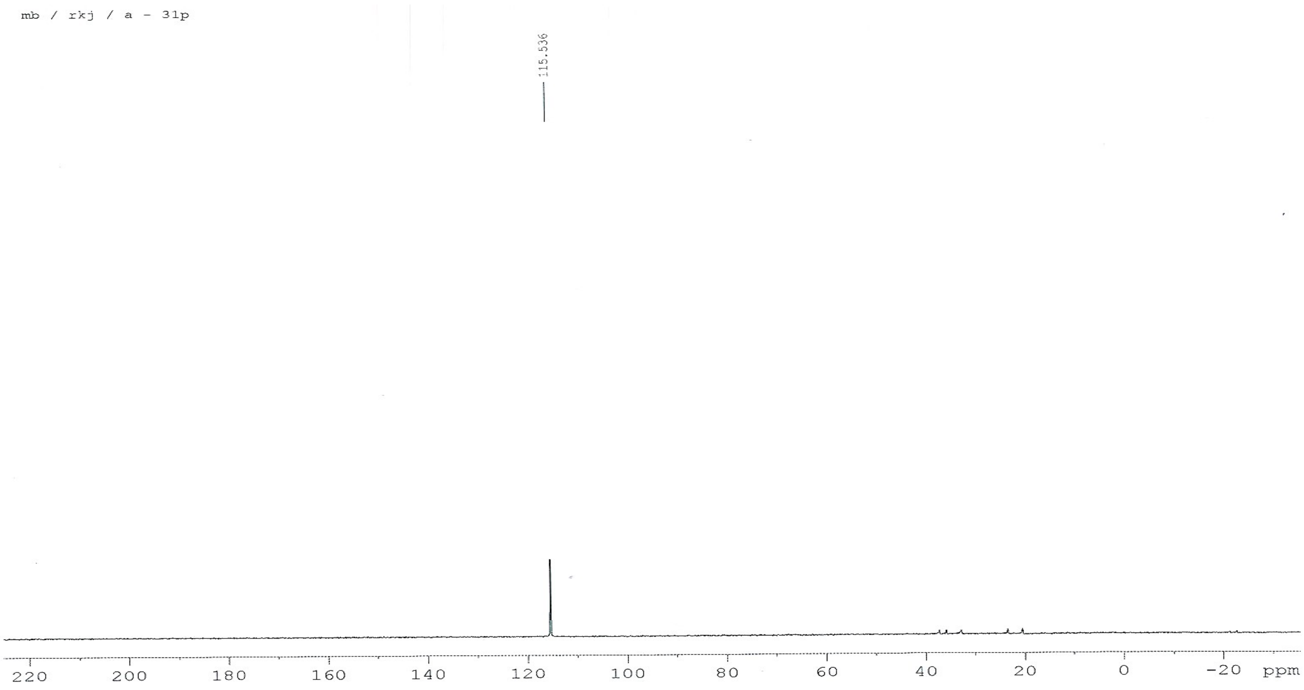

Figure S3 ^{31}P NMR (161.98 MHz) spectrum of L

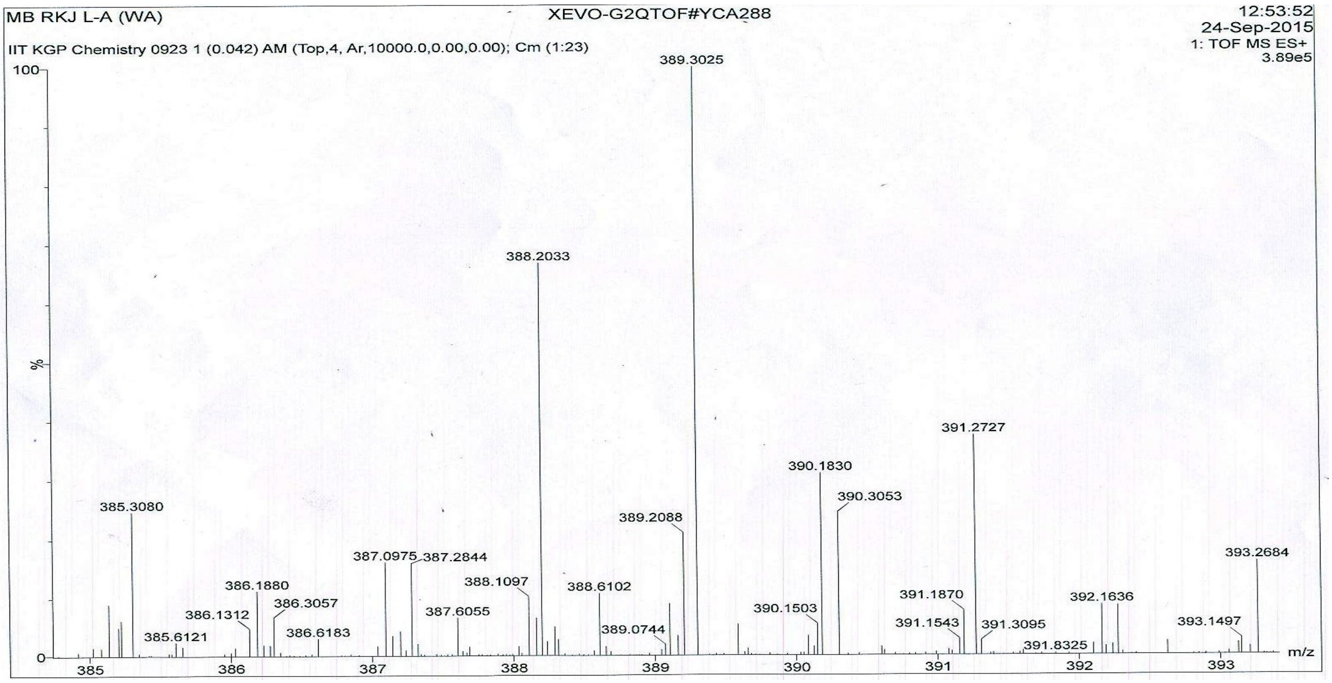

Figure S4 HRMS of L

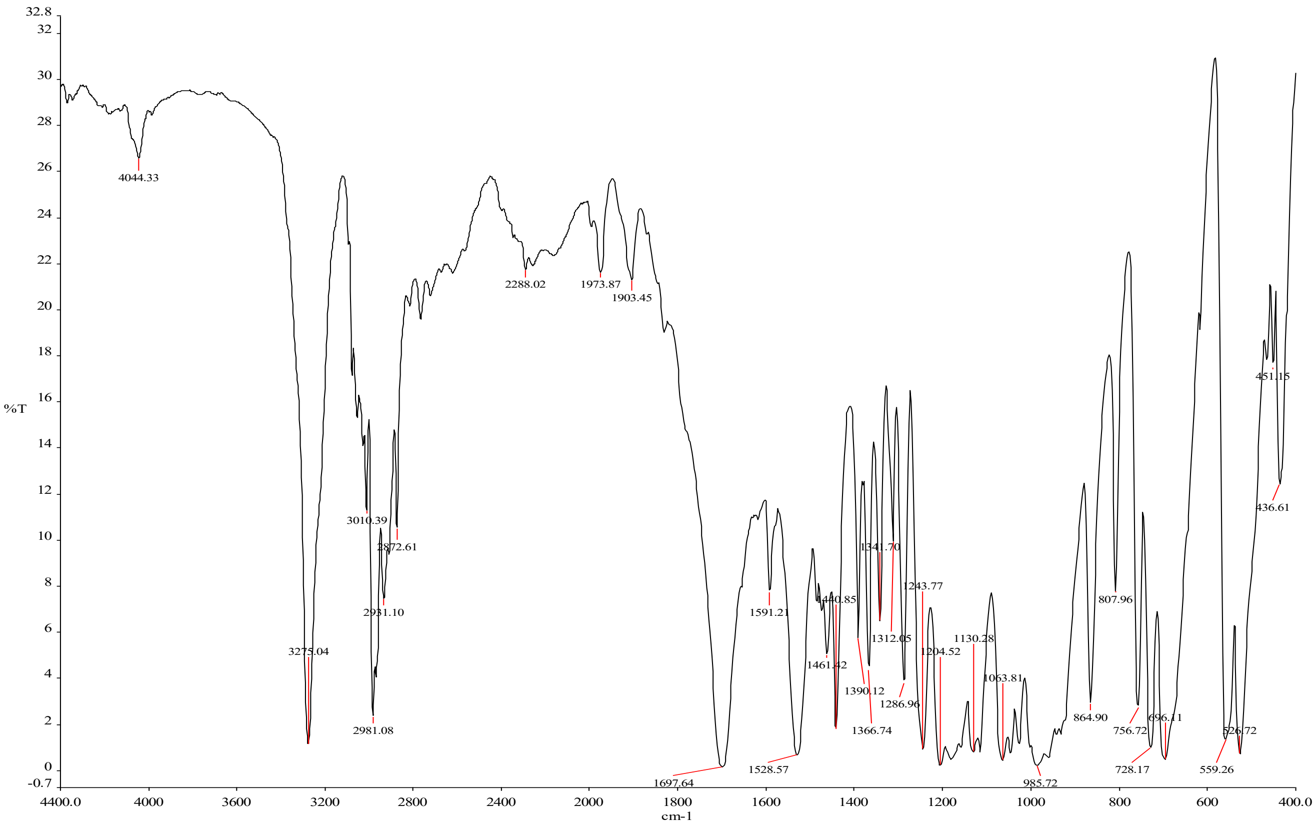

Figure S5 FT-IR spectrum of L

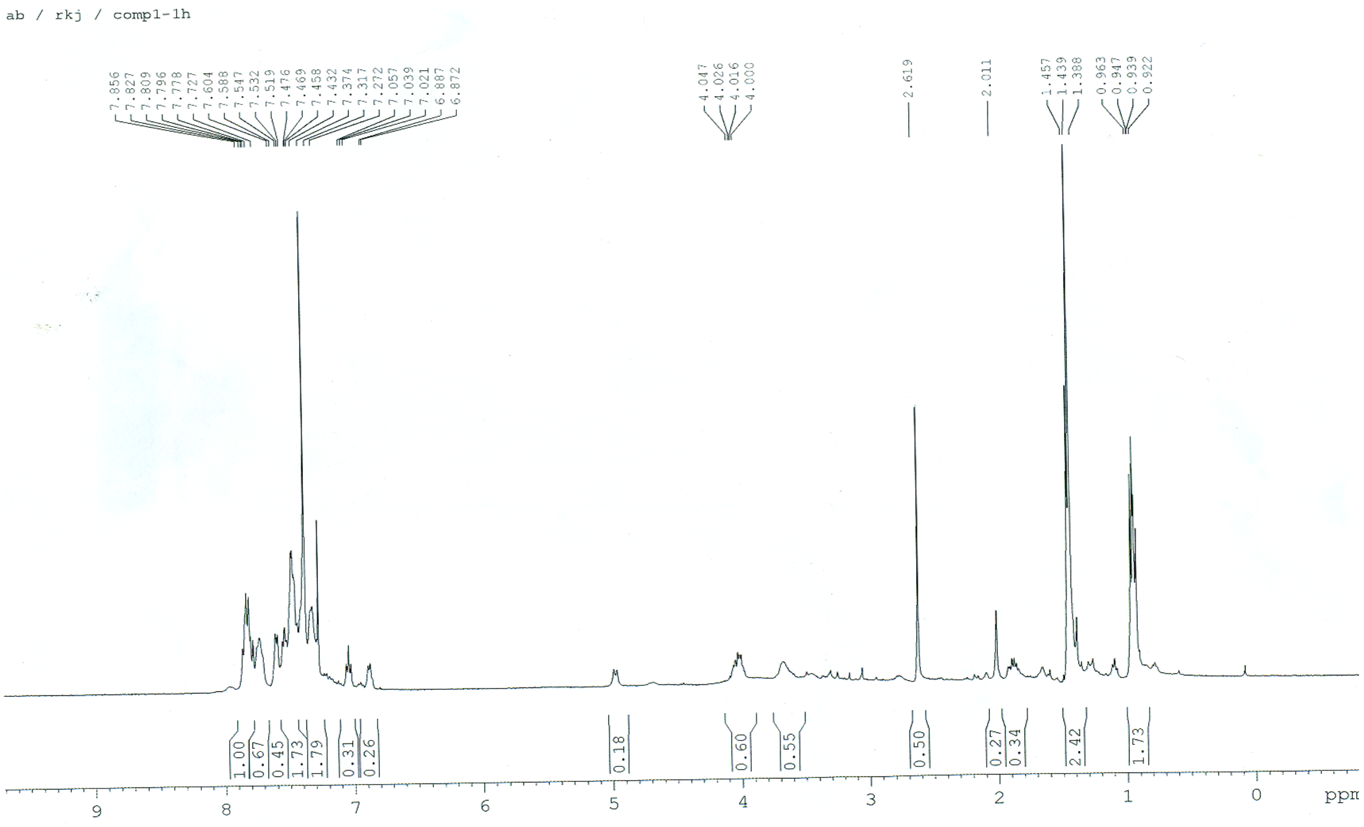

Figure S6 ^{1}H NMR (400 MHz) spectrum of 1

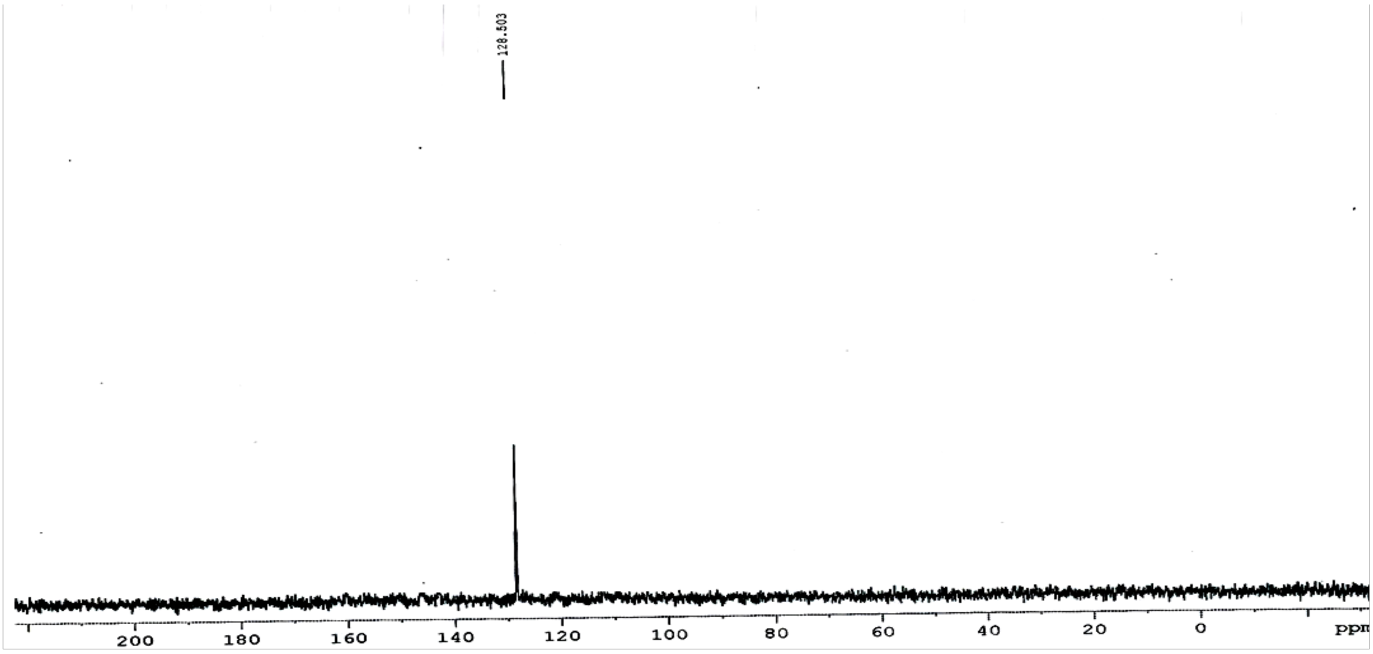

Figure S7 ^{31}P NMR (161.98 MHz) spectrum of 1

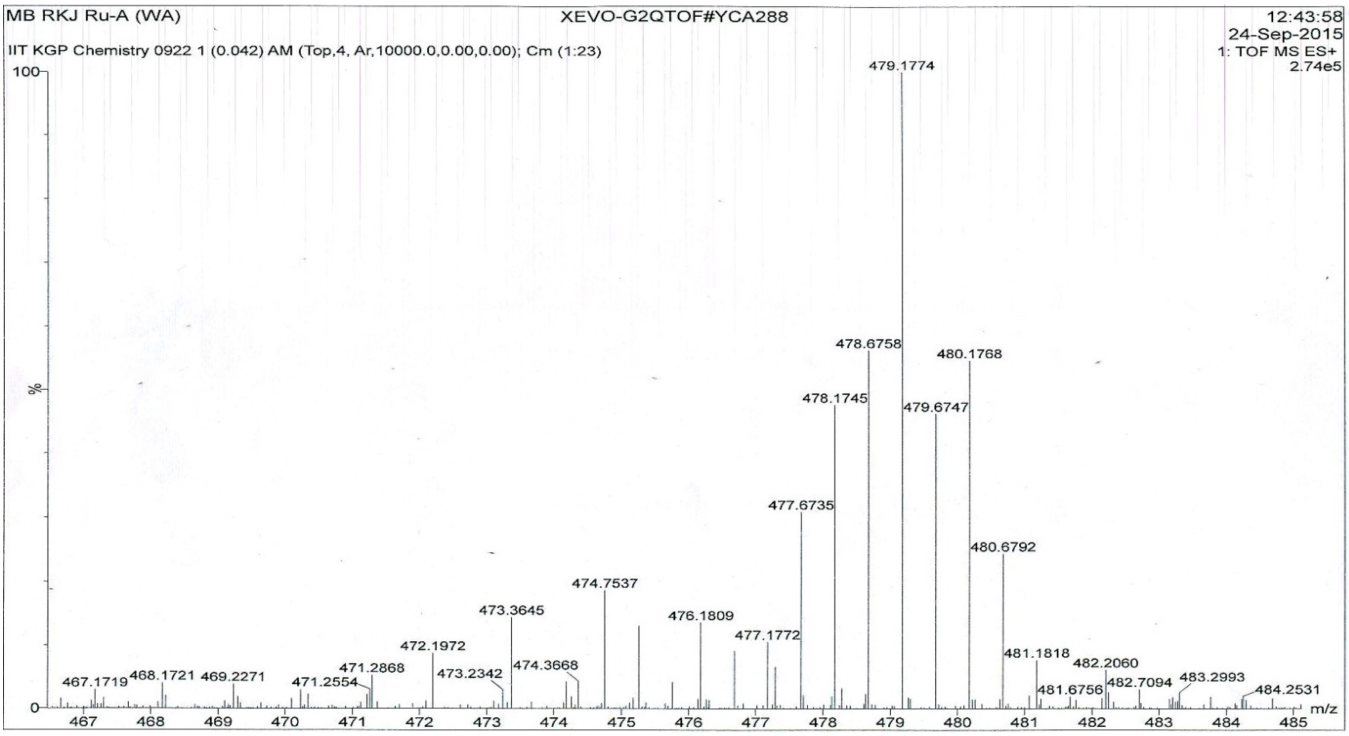

Figure S8 HRMS of 1

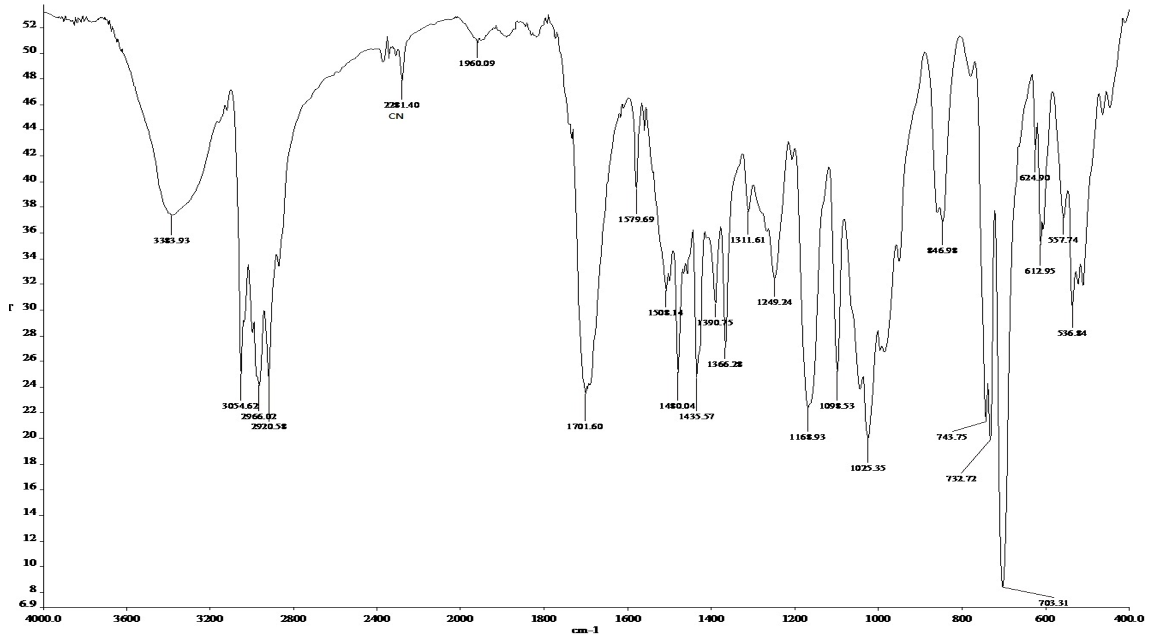

Figure S9 FT-IR spectrum of 1

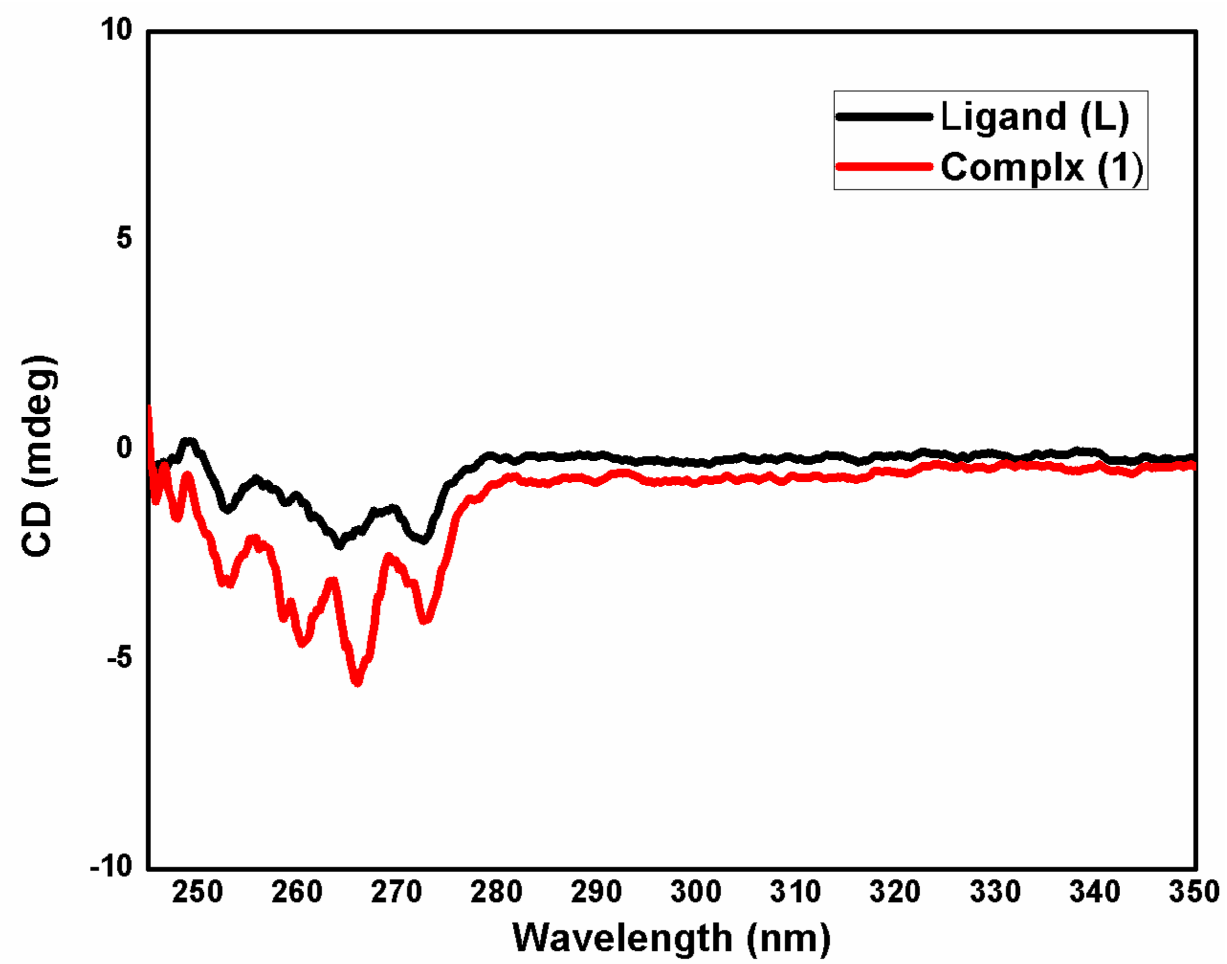

Figure S10 CD plot of L and 1

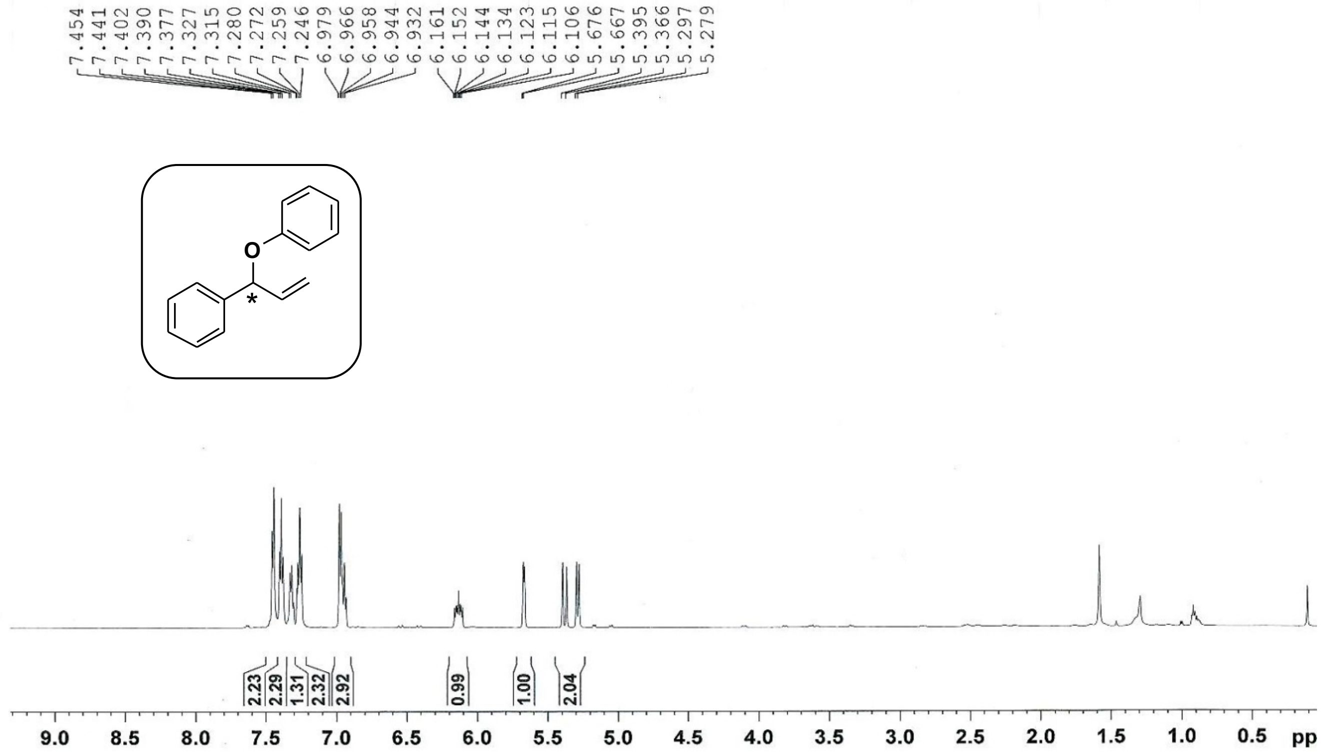

Figure S11 ^{1}H NMR (600 MHz) spectrum of 1-Phenyl-1-phenoxyprop-2-ene (4a) in $CDCl_3$.

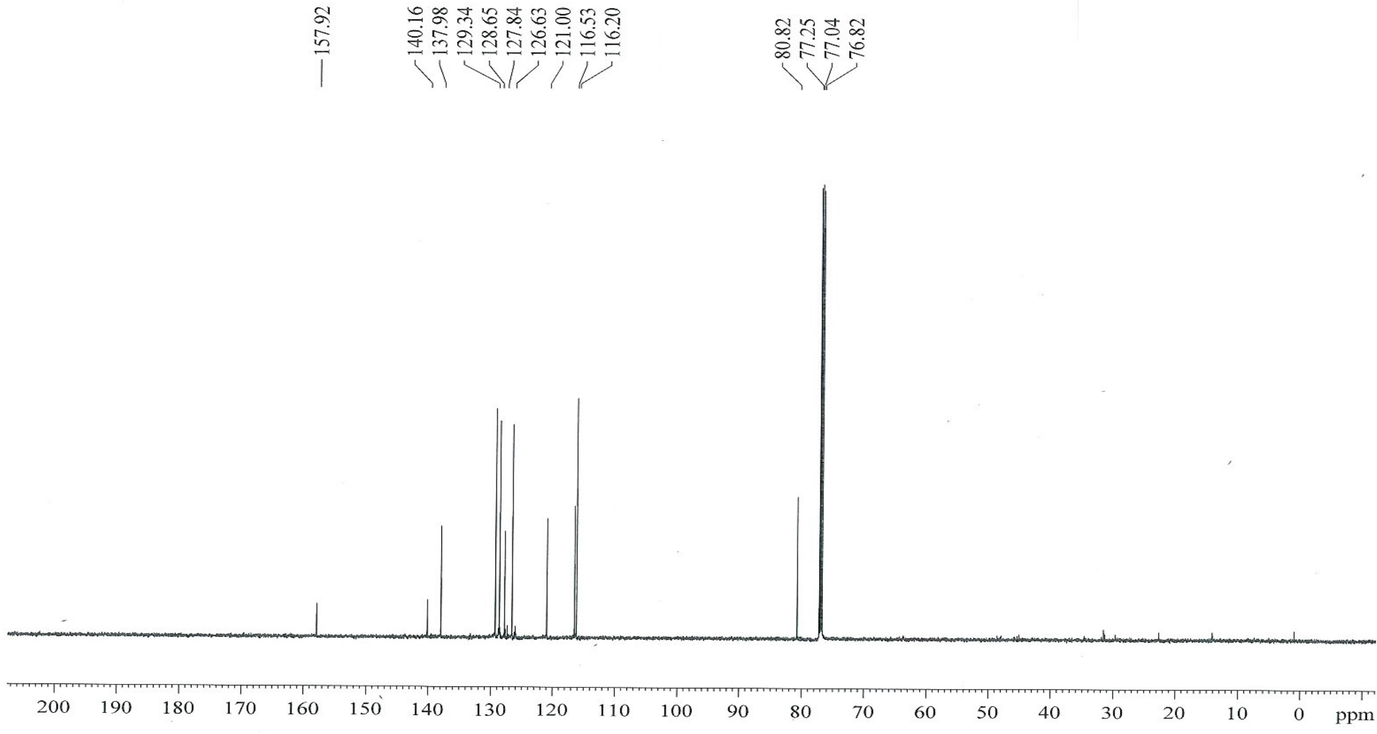

Figure S12 ^{13}C NMR (150 MHz) spectrum of 1-Phenyl-1-phenoxyprop-2-ene (4a) in $CDCl_3$.

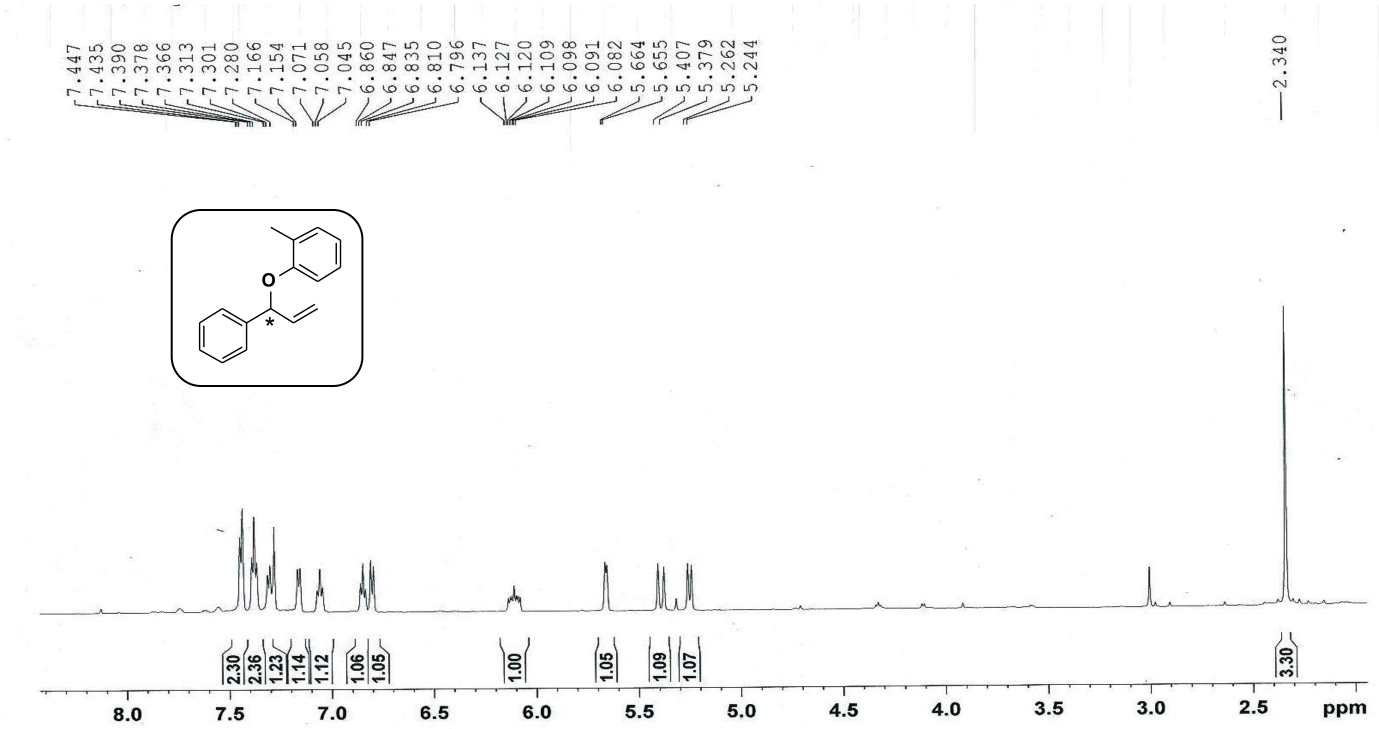

Figure S13 ^{1}H NMR (600 MHz) spectrum of 1-Phenyl-1-(o-methylphenoxy)prop-2-ene (4b) in $CDCl_3$.

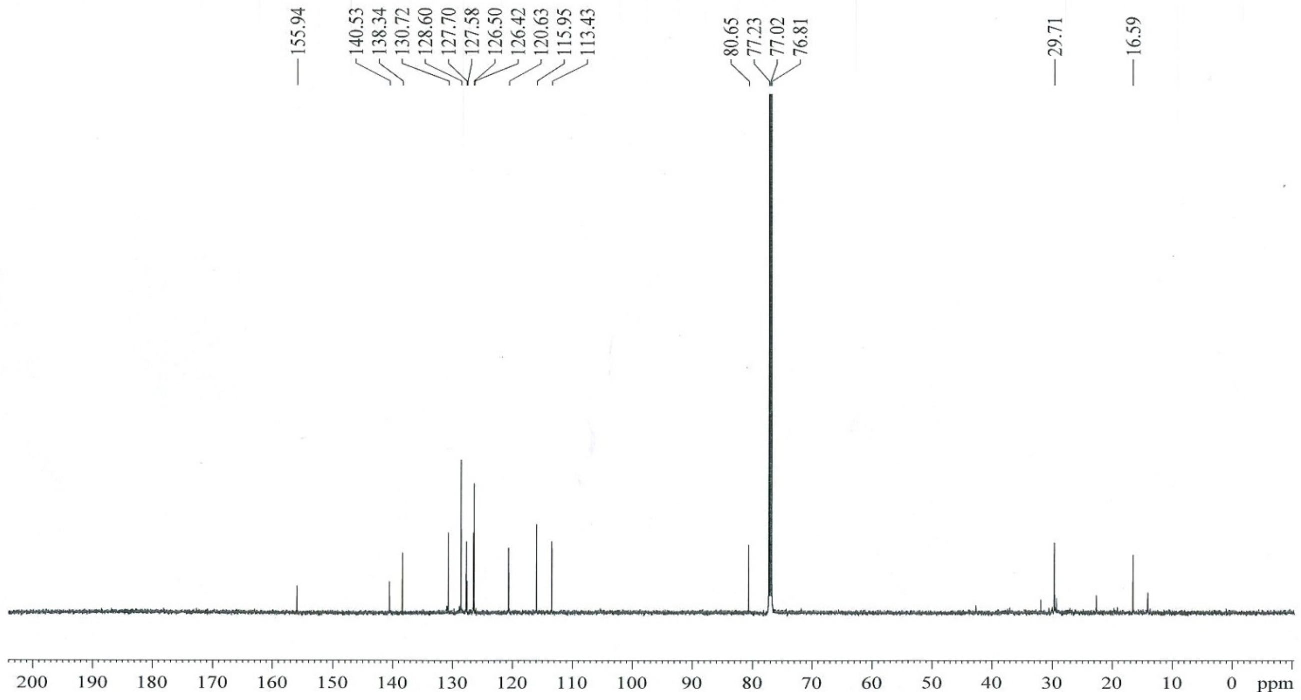

Figure S14 ^{13}C NMR (150 MHz) spectrum of 1-Phenyl-1-(o-methylphenoxy)prop-2-ene (4b) in $CDCl_3$.

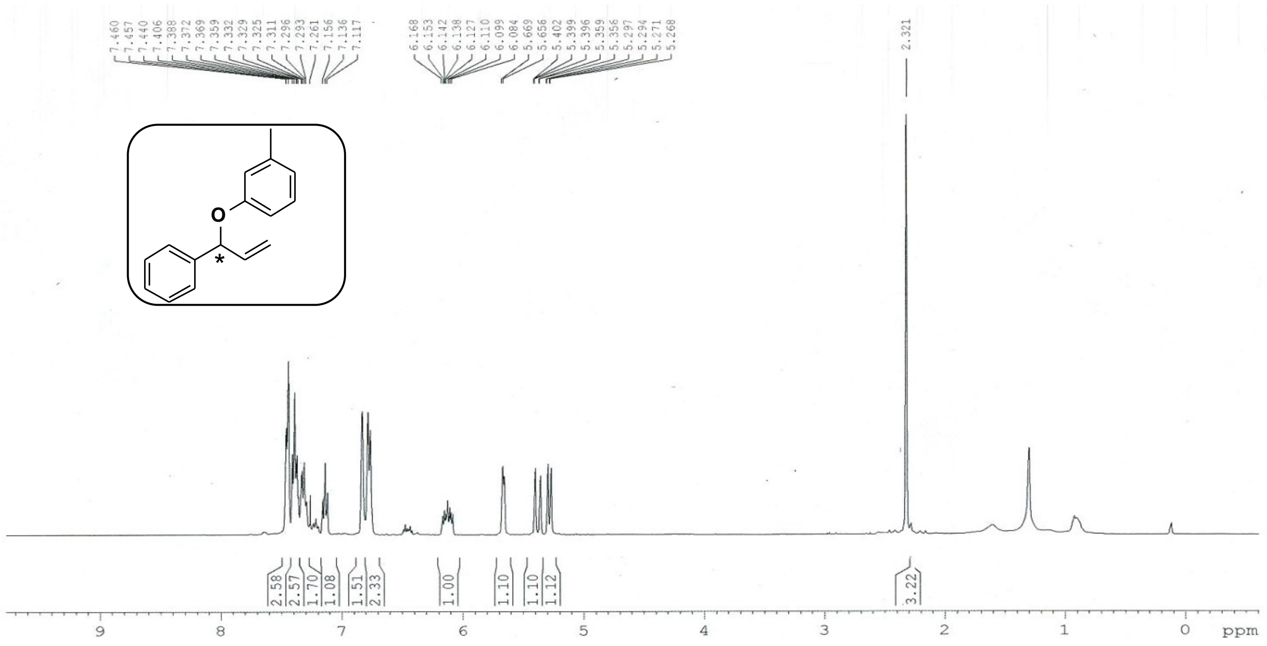

Figure S15 ^{1}H NMR (400 MHz) spectrum of 1-Phenyl-1-(m-methylphenoxy)prop-2-ene (4c) in $CDCl_3$.

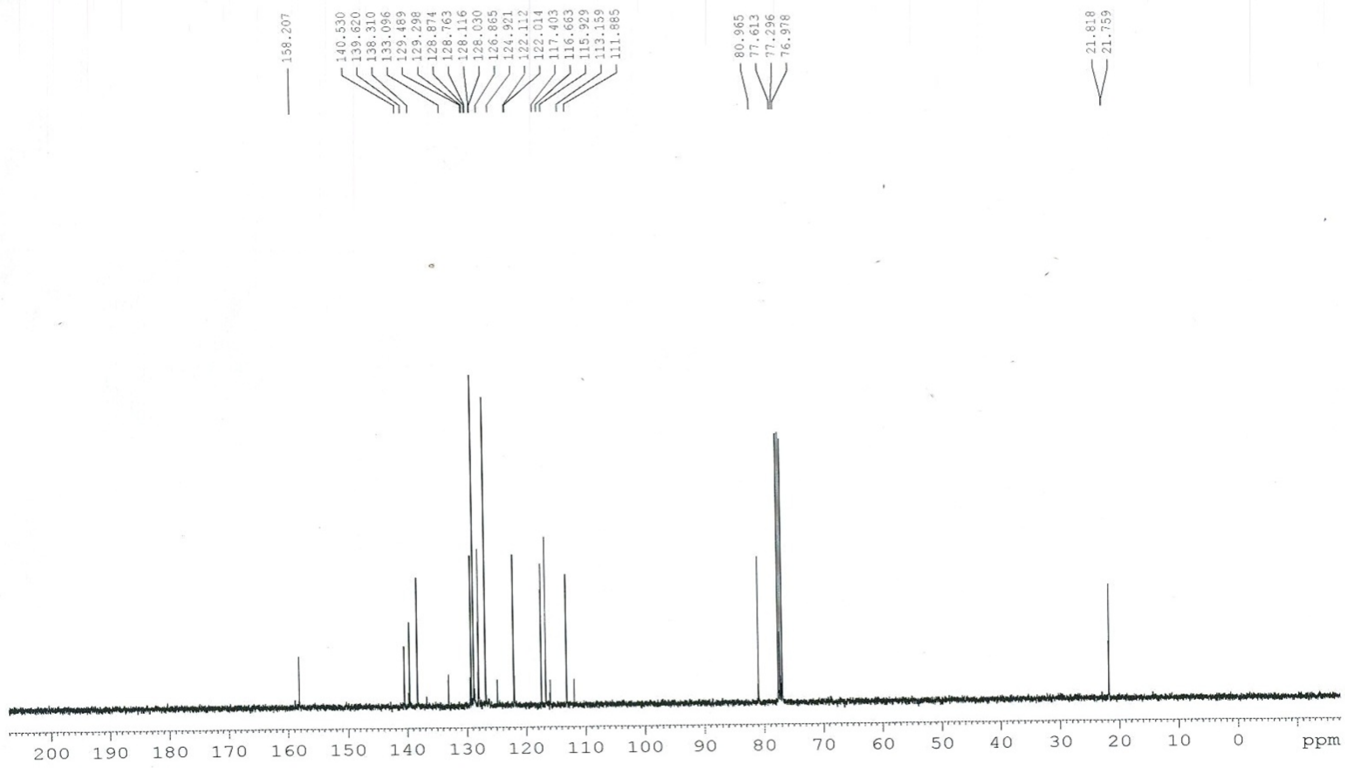

Figure S16 ^{13}C NMR (100 MHz) spectrum of 1-Phenyl-1-(m-methylphenoxy)prop-2-ene (4c) in $CDCl_3$.

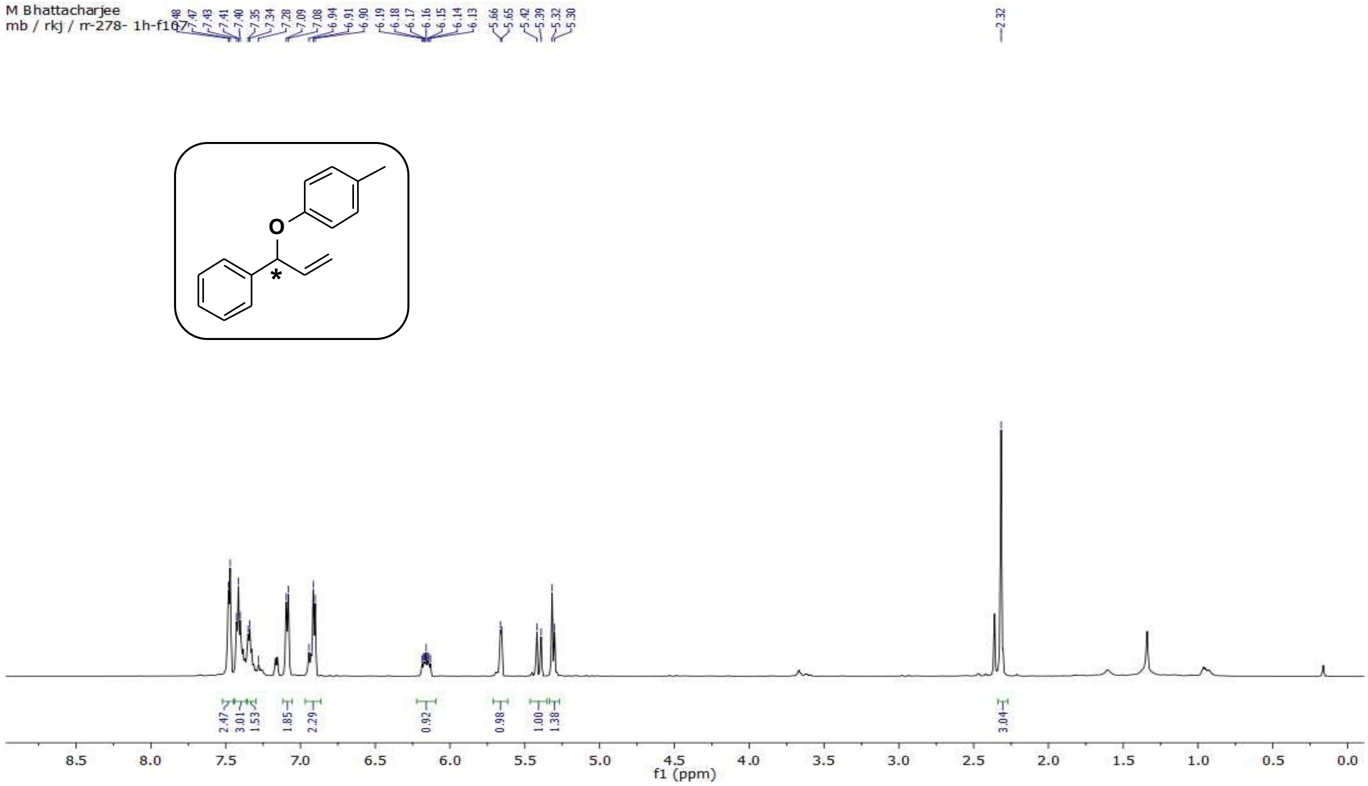

Figure S17 ^{1}H NMR (600 MHz) spectrum of 1-Phenyl-1-(p-methylphenoxy)prop-2-ene (4d) in $CDCl_3$.

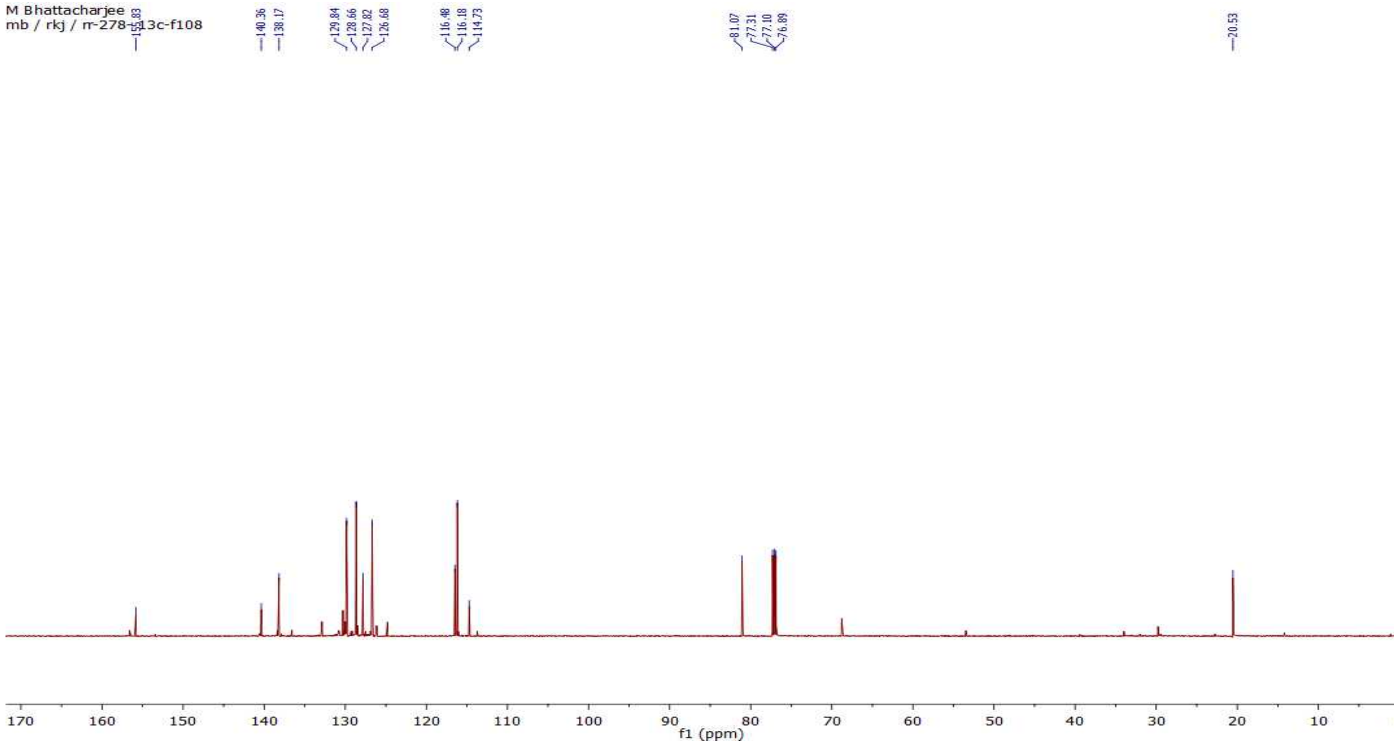

Figure S18 ^{13}C NMR (150 MHz) spectrum of 1-Phenyl-1-(p-methylphenoxy)prop-2-ene (4d) in $CDCl_3$.

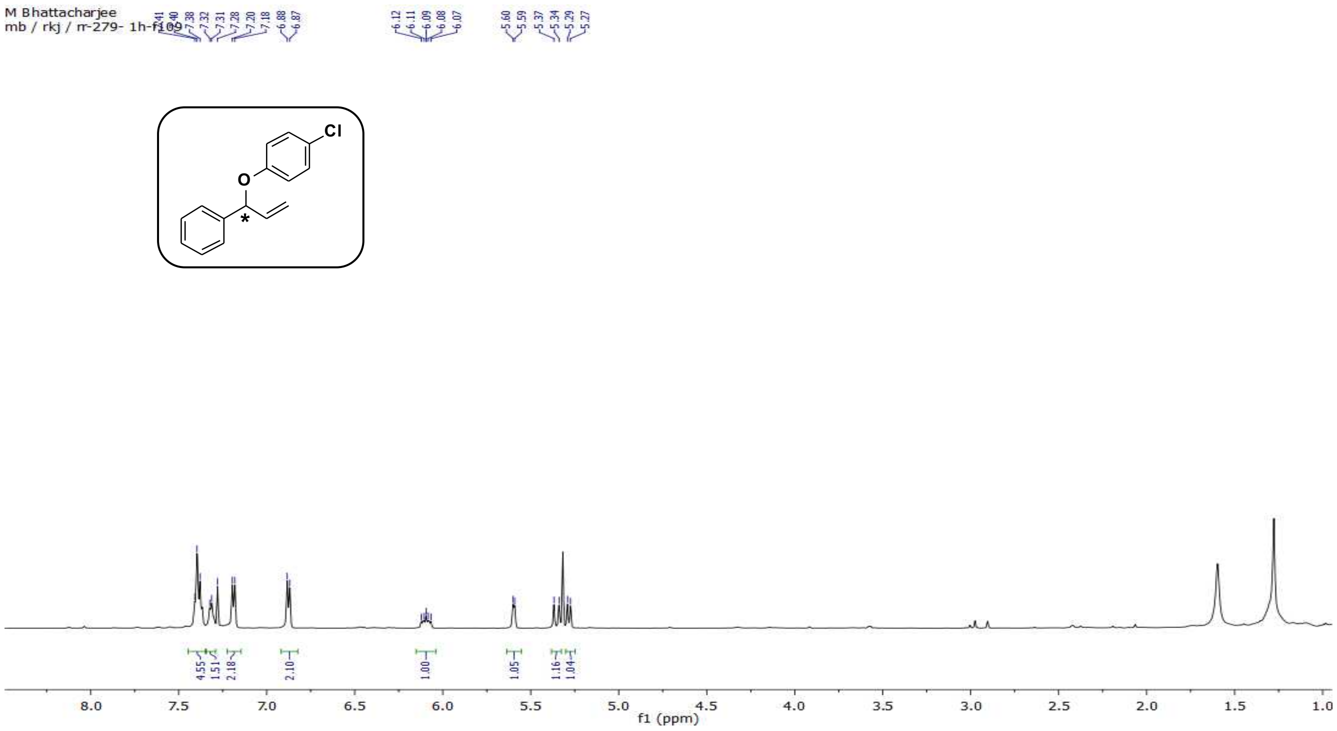

Figure S19 ^{1}H NMR (600 MHz) spectrum of 1-Phenyl-1-(p-chlorophenoxy)prop-2-ene (4e) in $CDCl_3$.

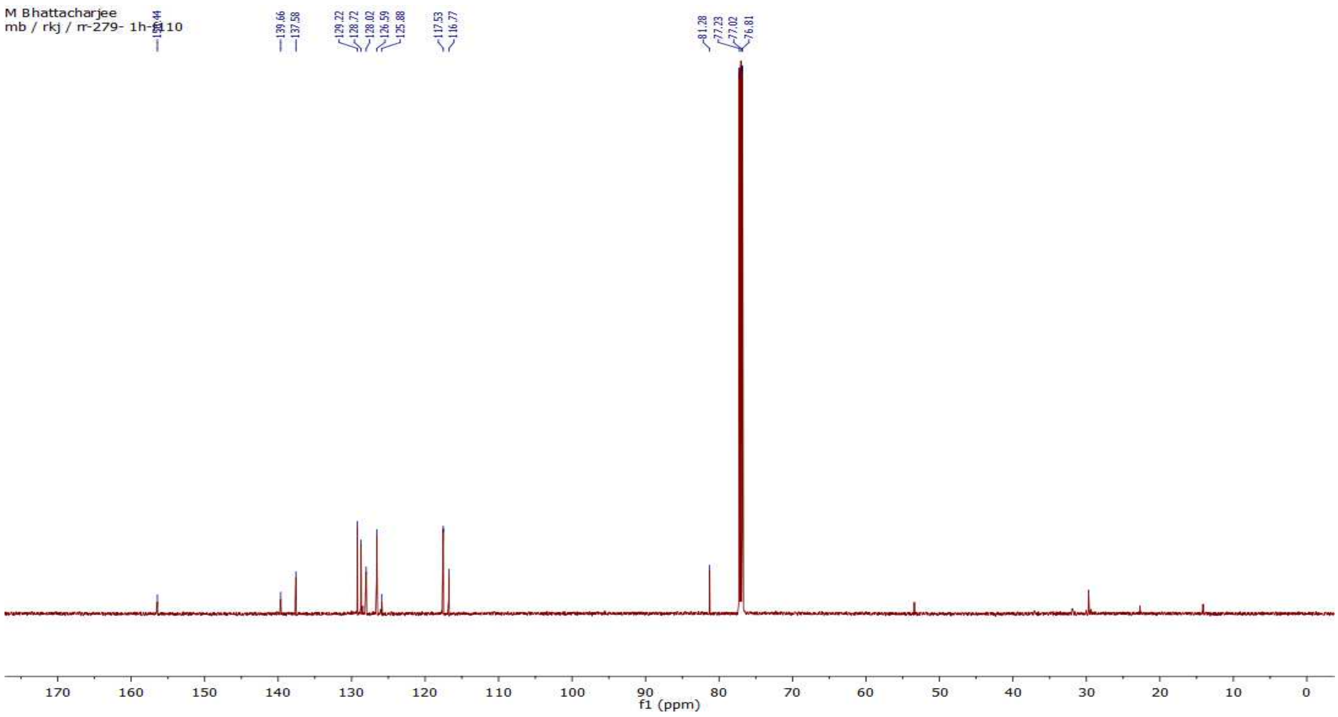

Figure S20 ^{13}C NMR (100 MHz) spectrum of 1-Phenyl-1-(*p*-chlorophenoxy)prop-2-ene (4e) in $CDCl_3$.

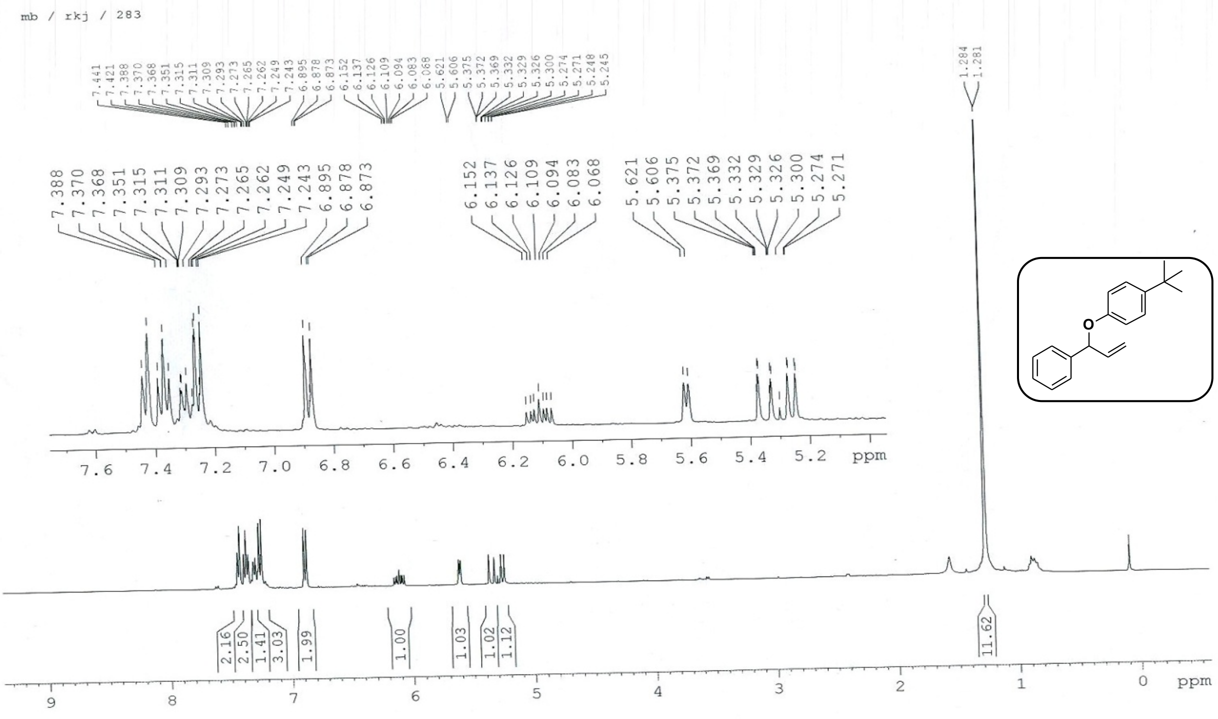

Figure S21 ^{1}H NMR (400 MHz) spectrum of 1-Phenyl-1-(*p*-tert-butylphenoxy)prop-2-ene (4f) in $CDCl_3$.

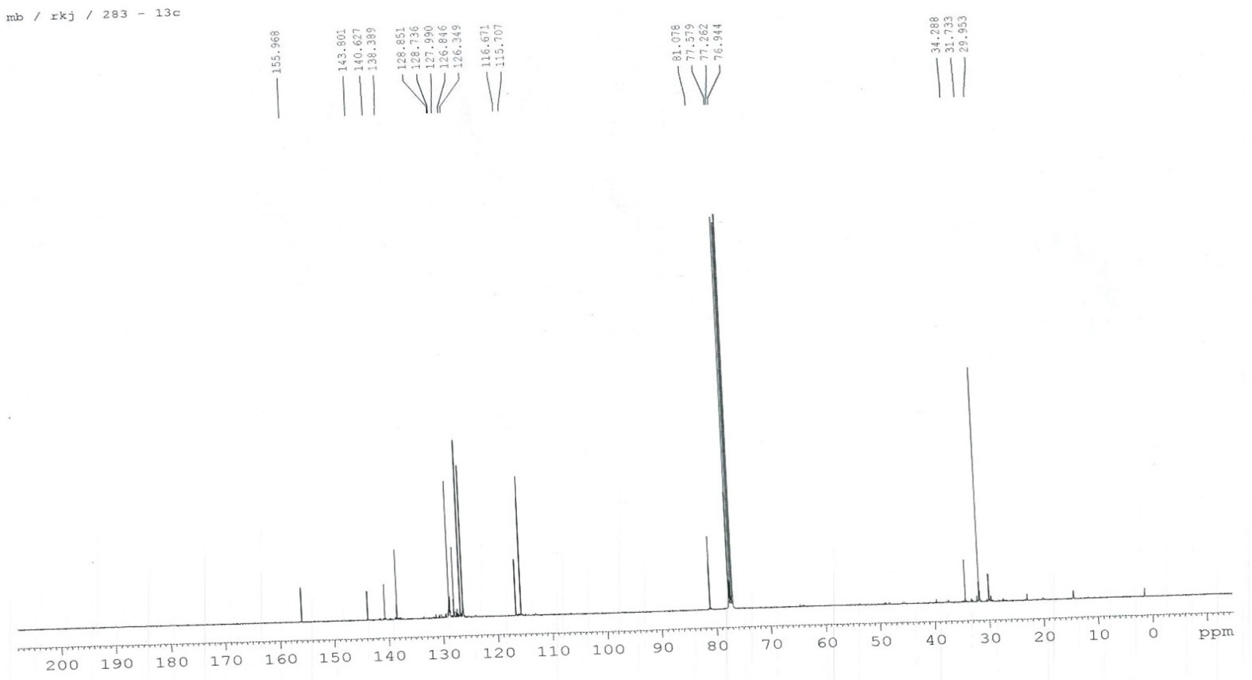

Figure S22 ^{13}C NMR (100 MHz) spectrum of 1-Phenyl-1-(*p*-tert-butylphenoxy)prop-2-ene (4f) in $CDCl_3$.

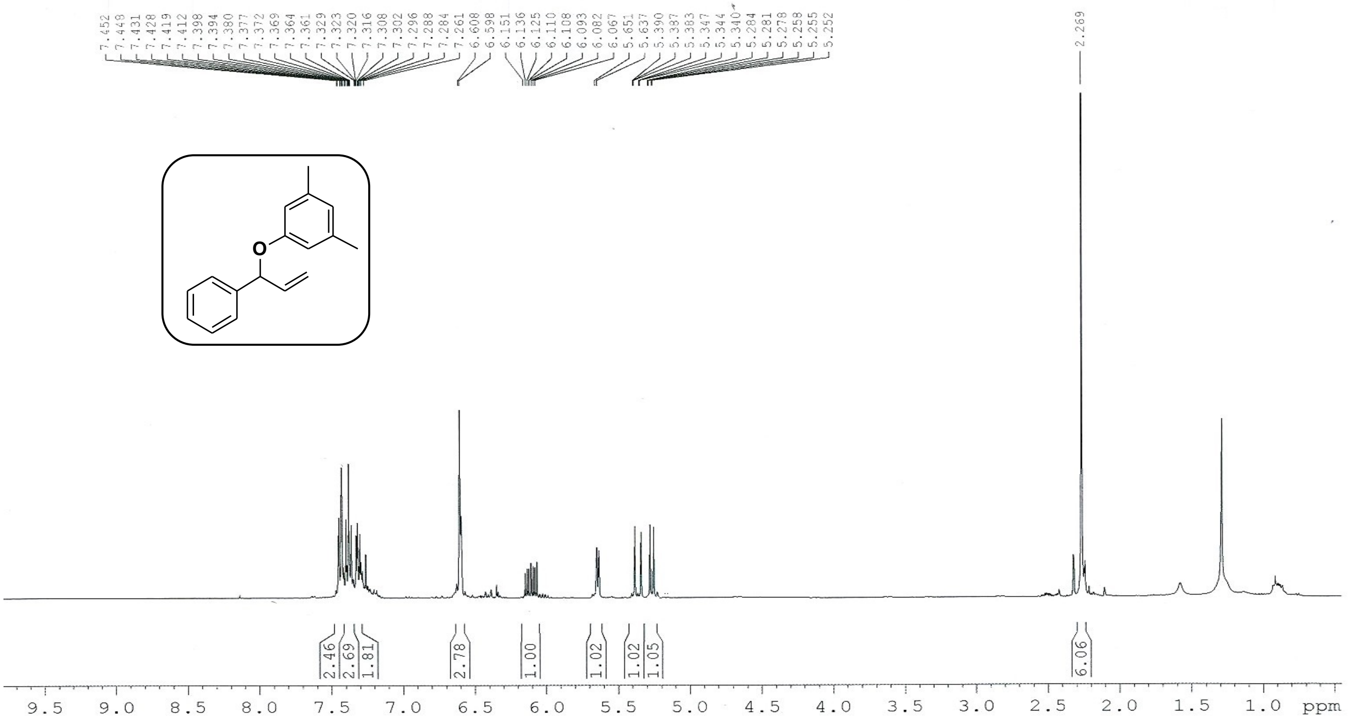

Figure S23 ^{1}H NMR (400 MHz) spectrum of 1-Phenyl-1-(3,5-dimethylphenoxy)prop-2-ene (4g) in $CDCl_3$.

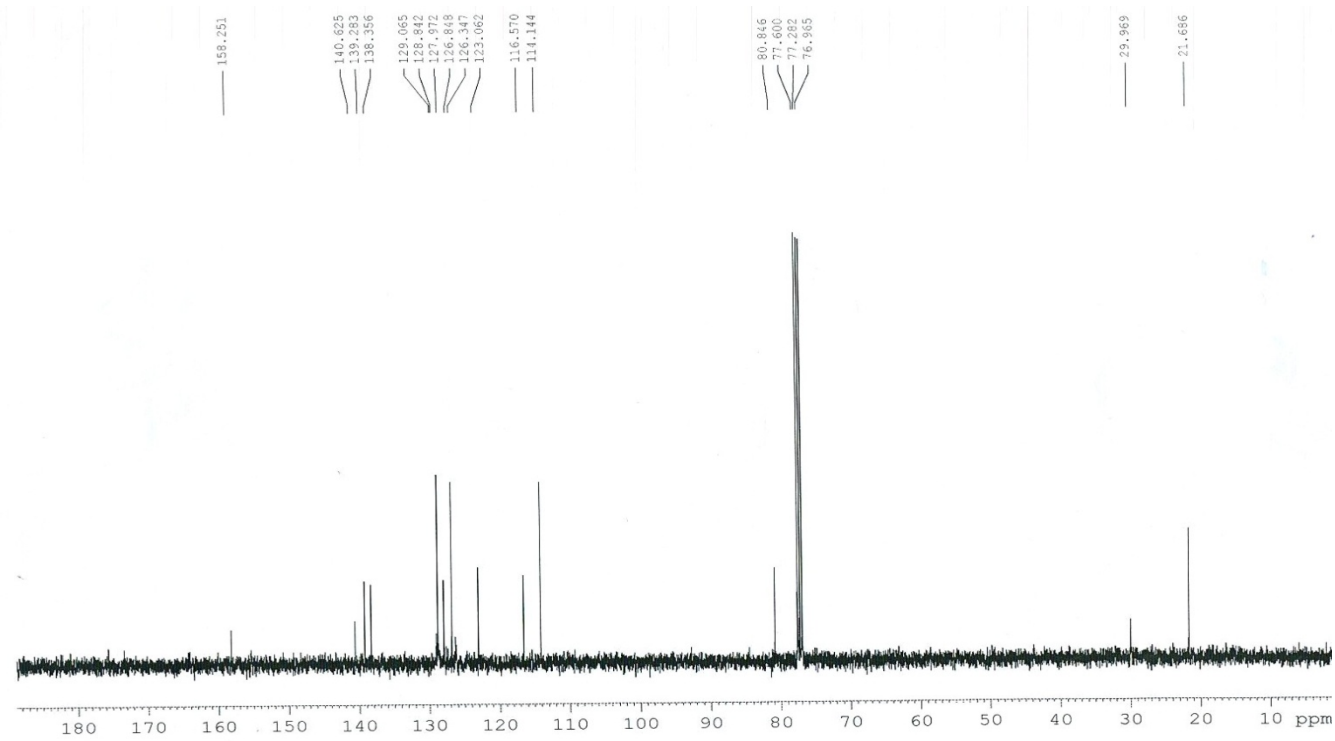

Figure S24 ^{13}C NMR (400 MHz) spectrum of 1-Phenyl-1-(3,5-dimethylphenoxy)prop-2-ene (4g) in $CDCl_3$.

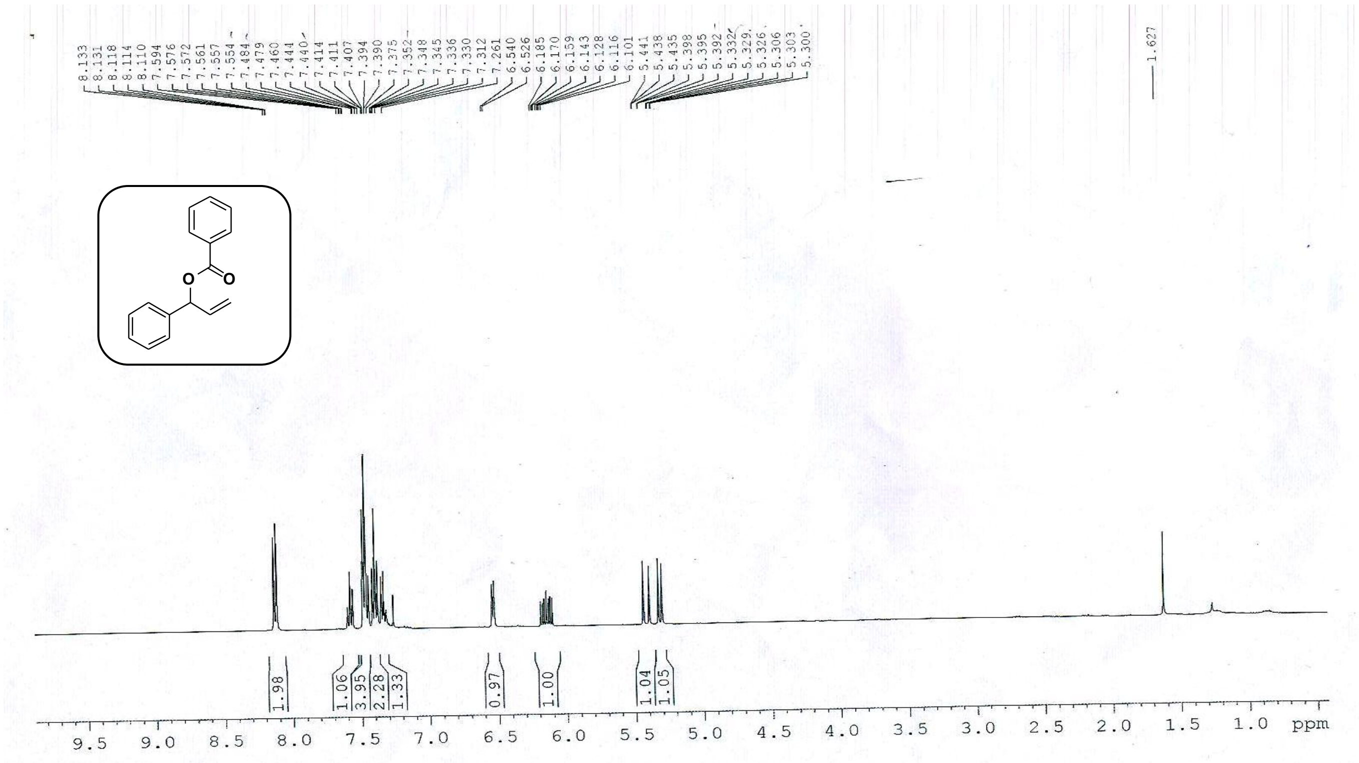

Figure S25 ^{1}H NMR (400 MHz) spectrum of 1-Phenyl-2-propenyl benzoate (7a) in $CDCl_3$.

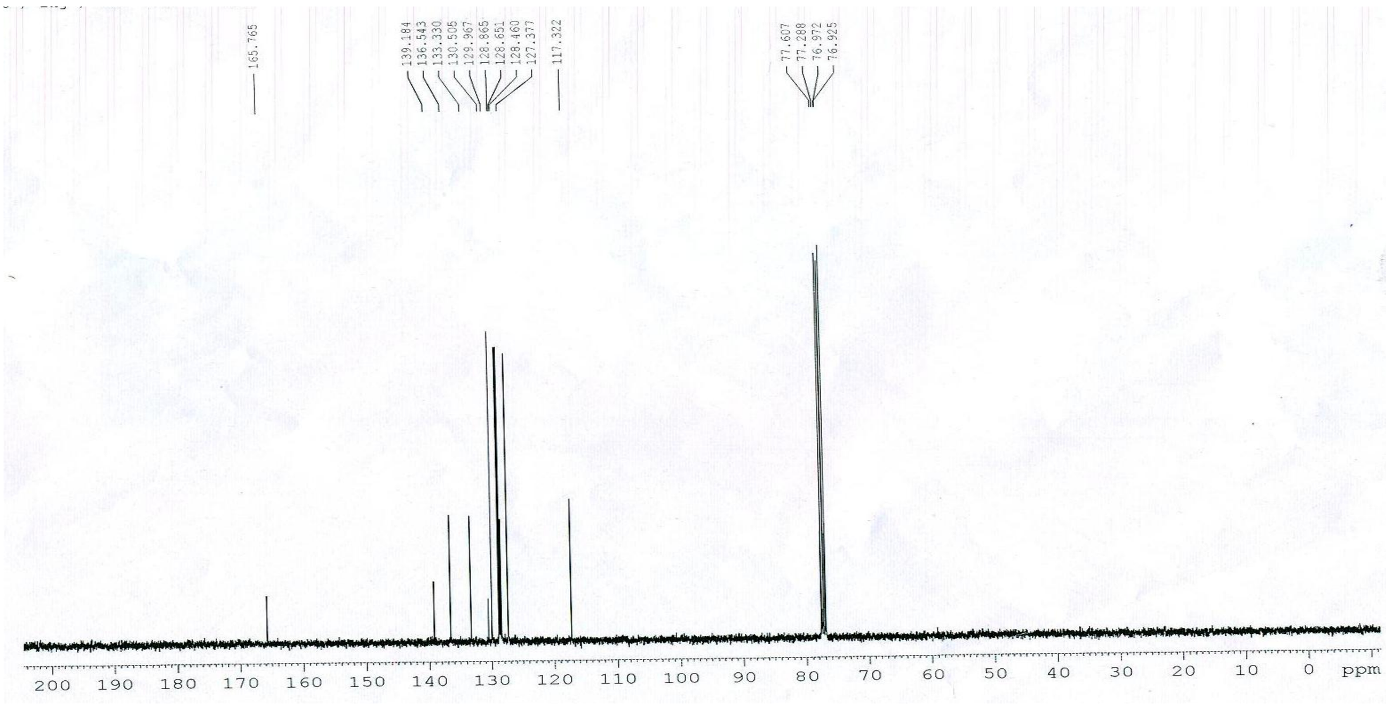

Figure S26 ^{13}C NMR (100 MHz) spectrum of 1-Phenyl-2-propenyl benzoate (7a) in $CDCl_3$.

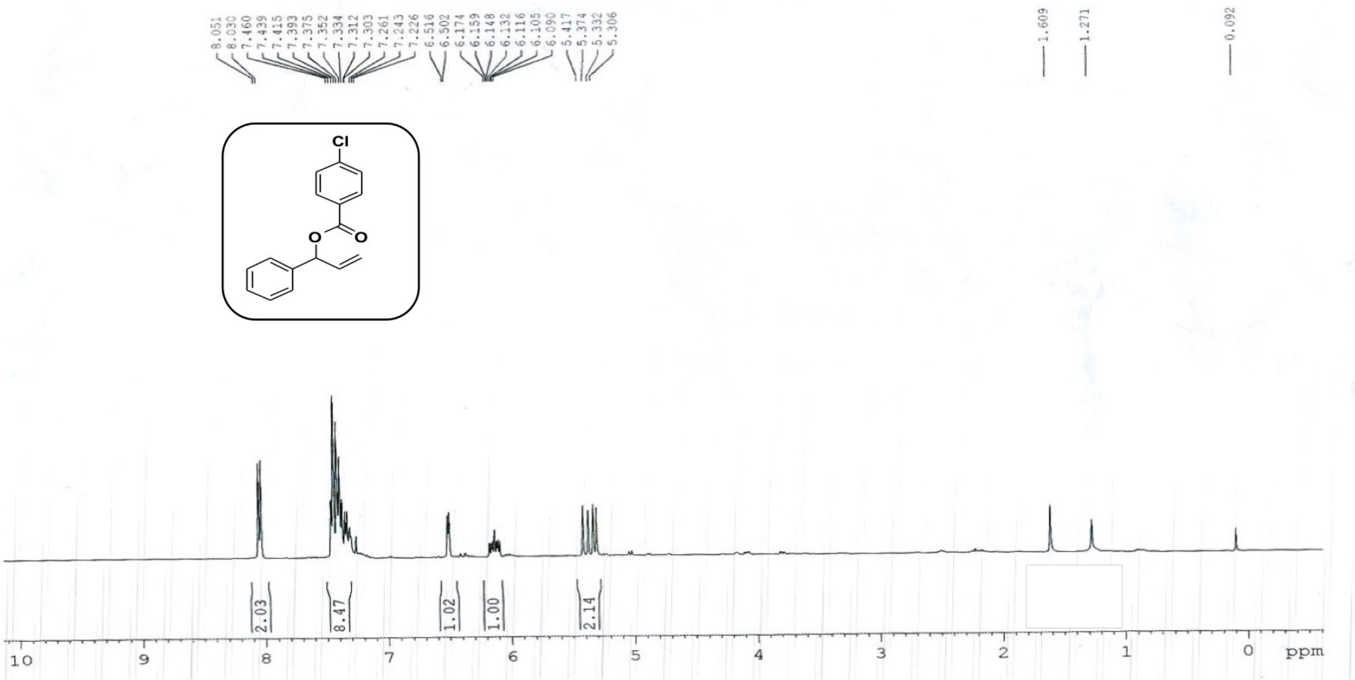

Figure S27 ^{1}H NMR (400 MHz) spectrum of 1-Phenyl-2-propenyl-4-chlorobenzoate(7b) in $CDCl_3$.

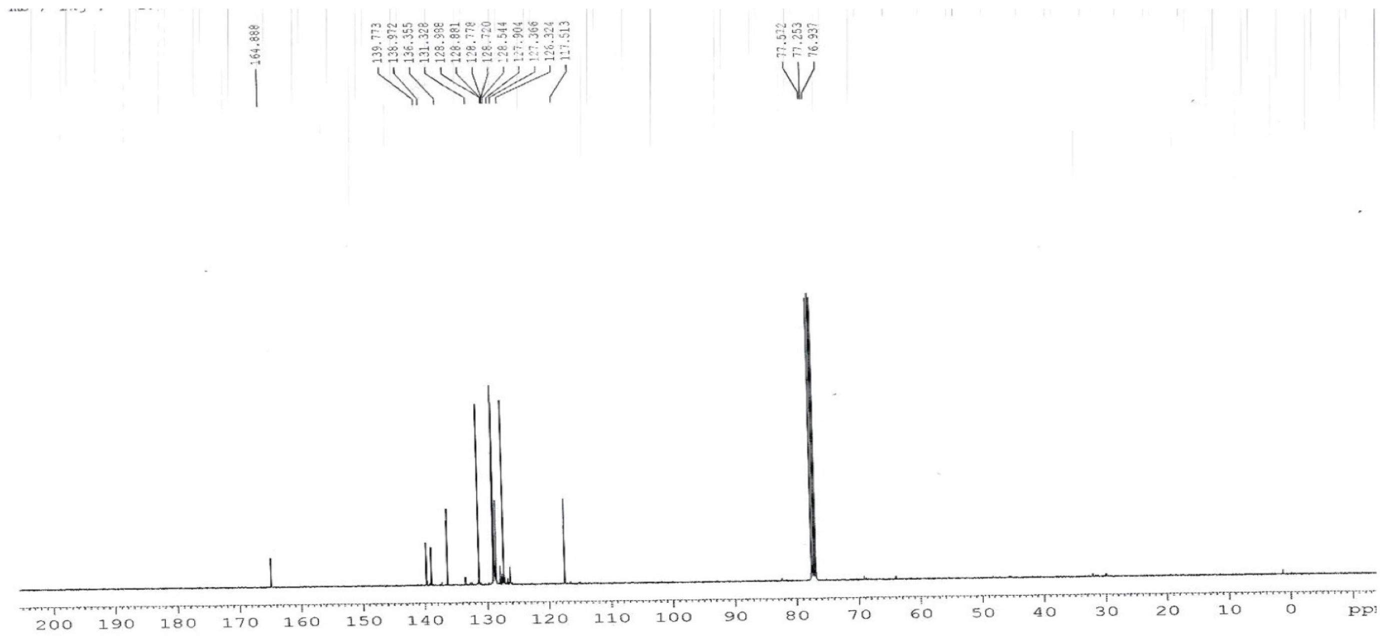

Figure S28 ^{13}C NMR (100 MHz) spectrum of 1-Phenyl-2-propenyl-4-chlorobenzoate (7b) in $CDCl_3$.

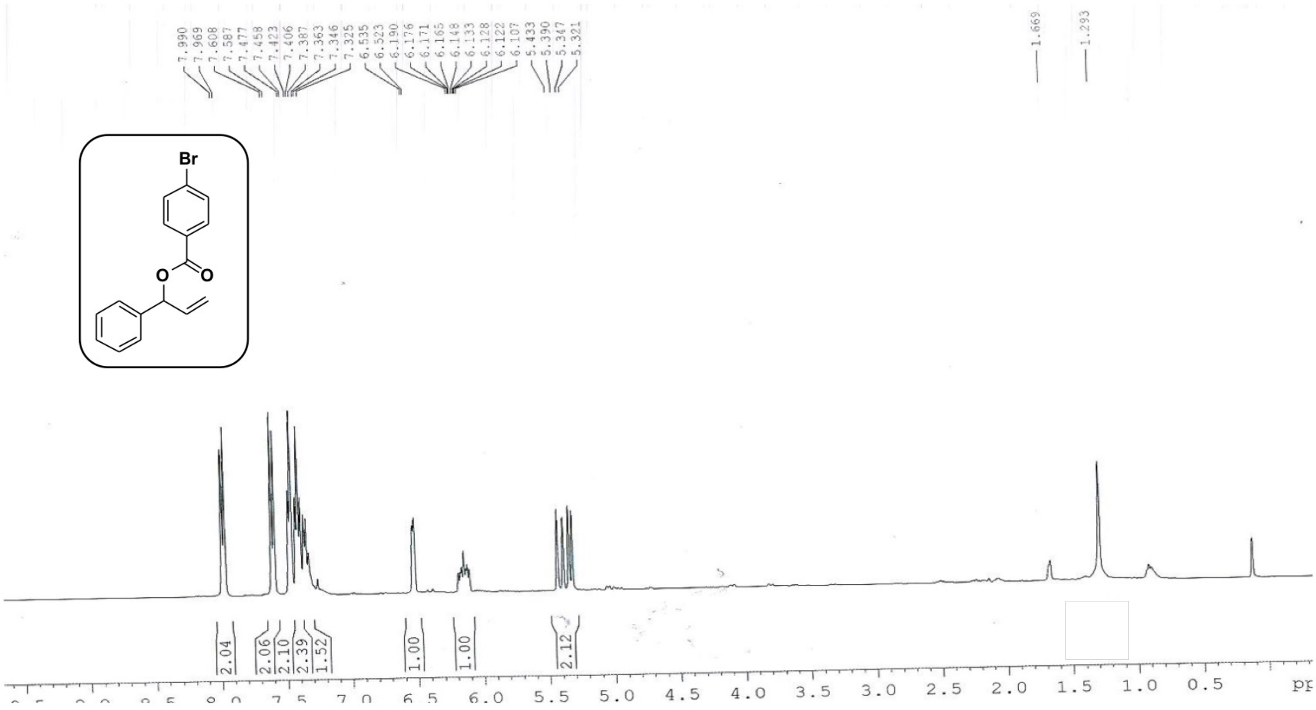

Figure S29 ^{1}H NMR (400 MHz) spectrum of 1-Phenyl-2-propenyl4-bromobenzoate (7c) in $CDCl_3$.

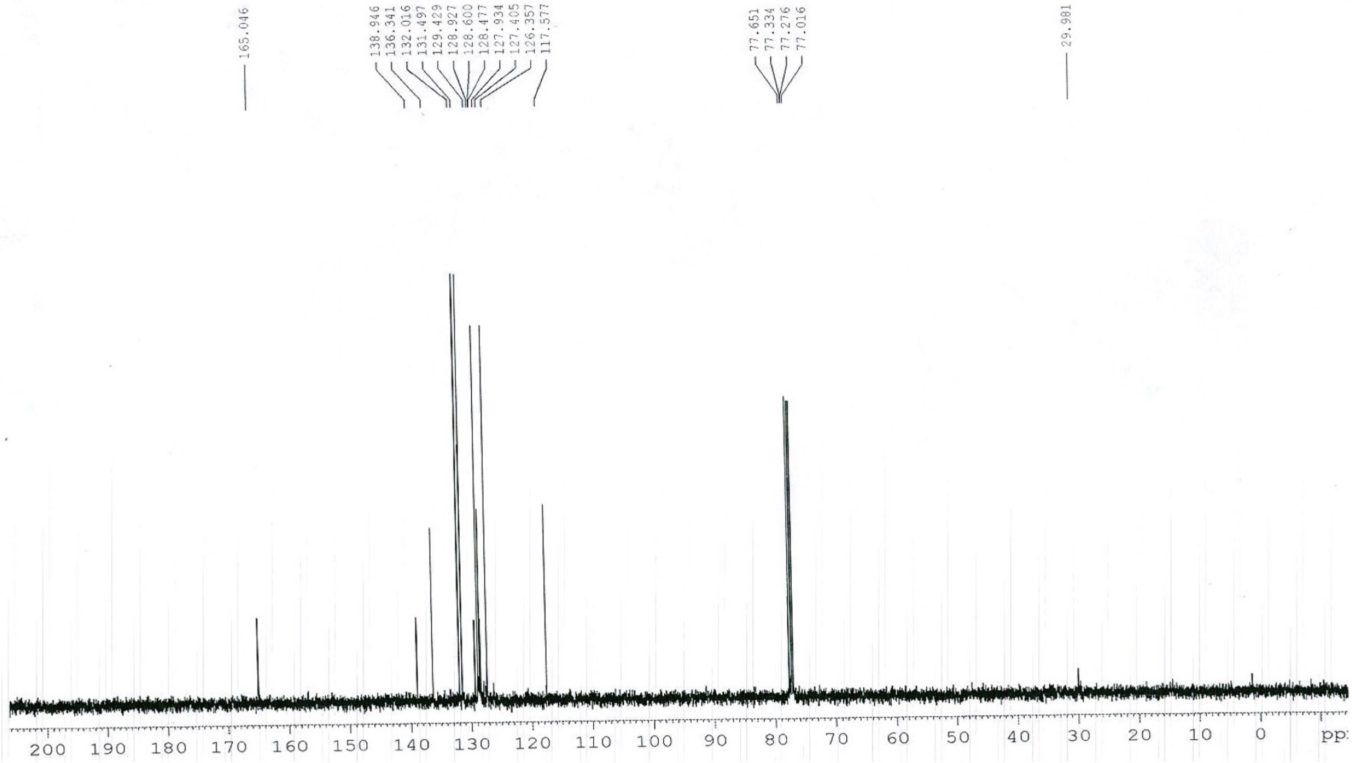

Figure S30 ^{13}C NMR (100 MHz) spectrum of 1-Phenyl-2-propenyl4-bromobenzoate (7c) in $CDCl_3$.

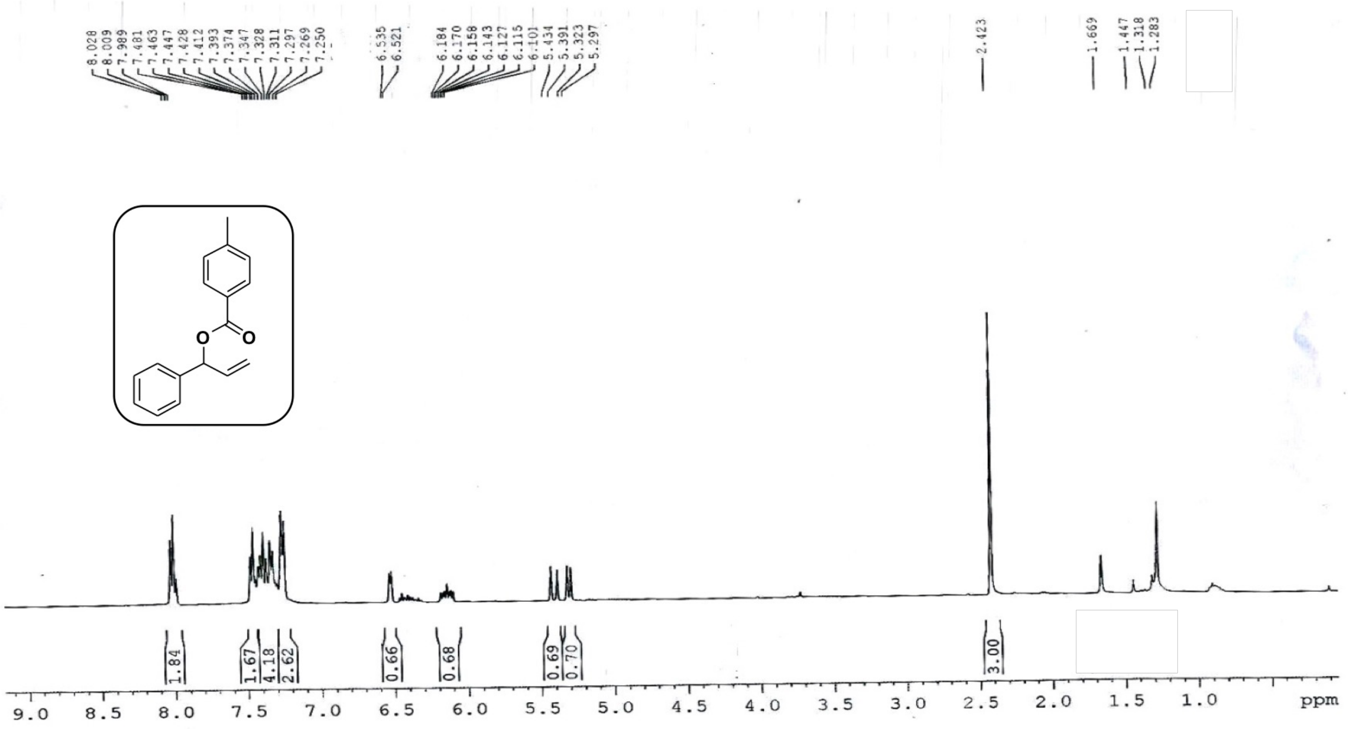

Figure S31 ^{1}H NMR (400MHz) spectrum of 1-Phenyl-2-propenyl 4-methylbenzoate (7d) in $CDCl_3$.

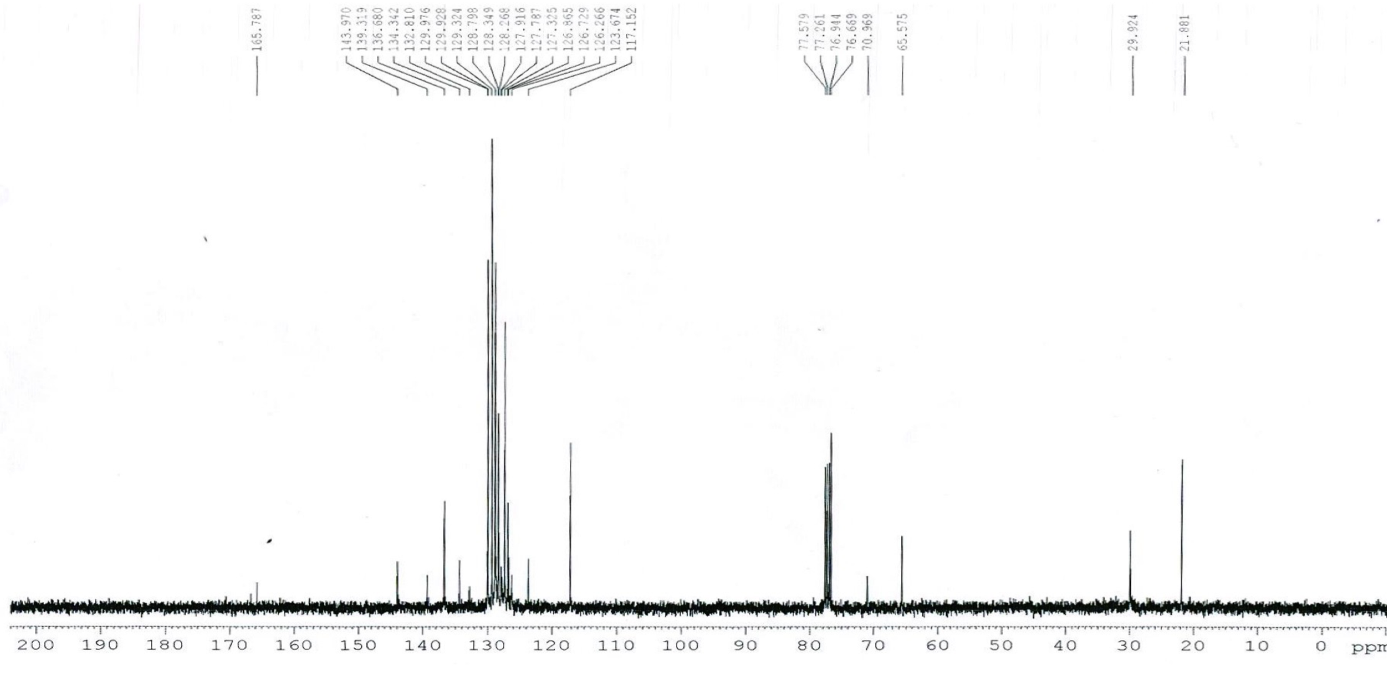

Figure S32 ^{13}C NMR (100MHz) spectrum of 1-Phenyl-2-propenyl 4-methylbenzoate (7d) in $CDCl_3$.

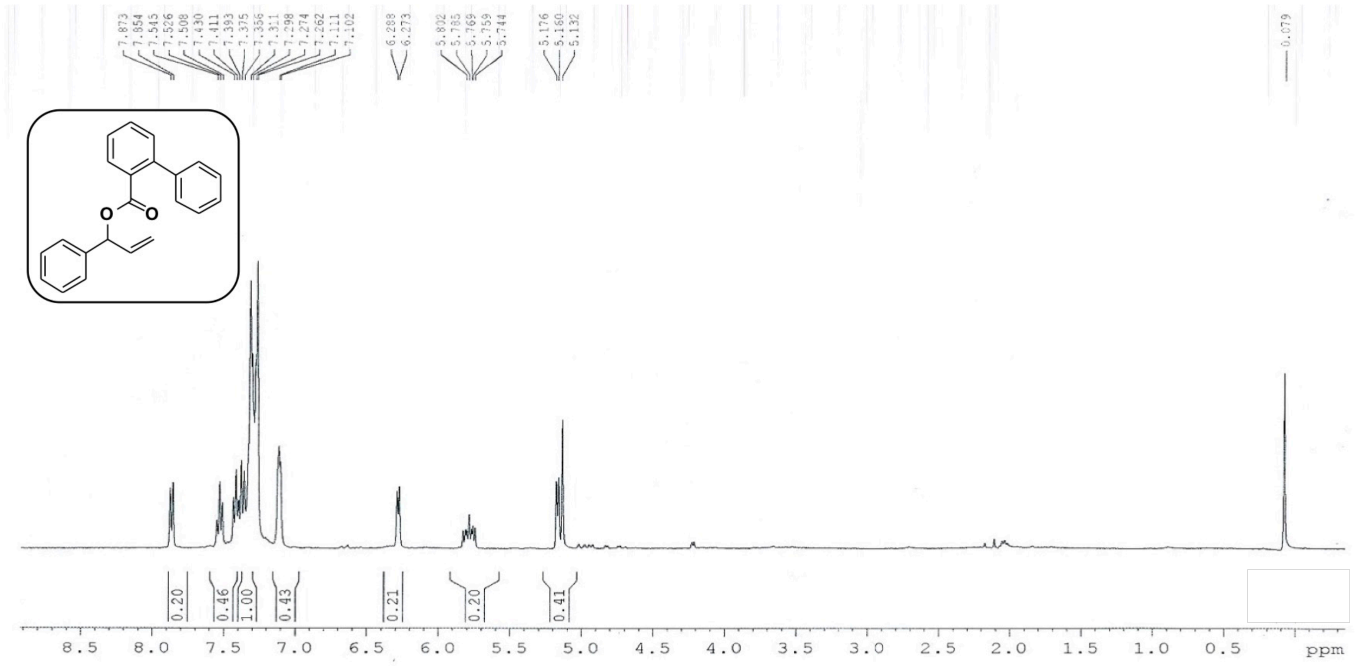

Figure S33 ^{1}H NMR (400MHz) spectrum of 1-phenylallyl biphenyl-2-carboxylate (7e) in $CDCl_3$.

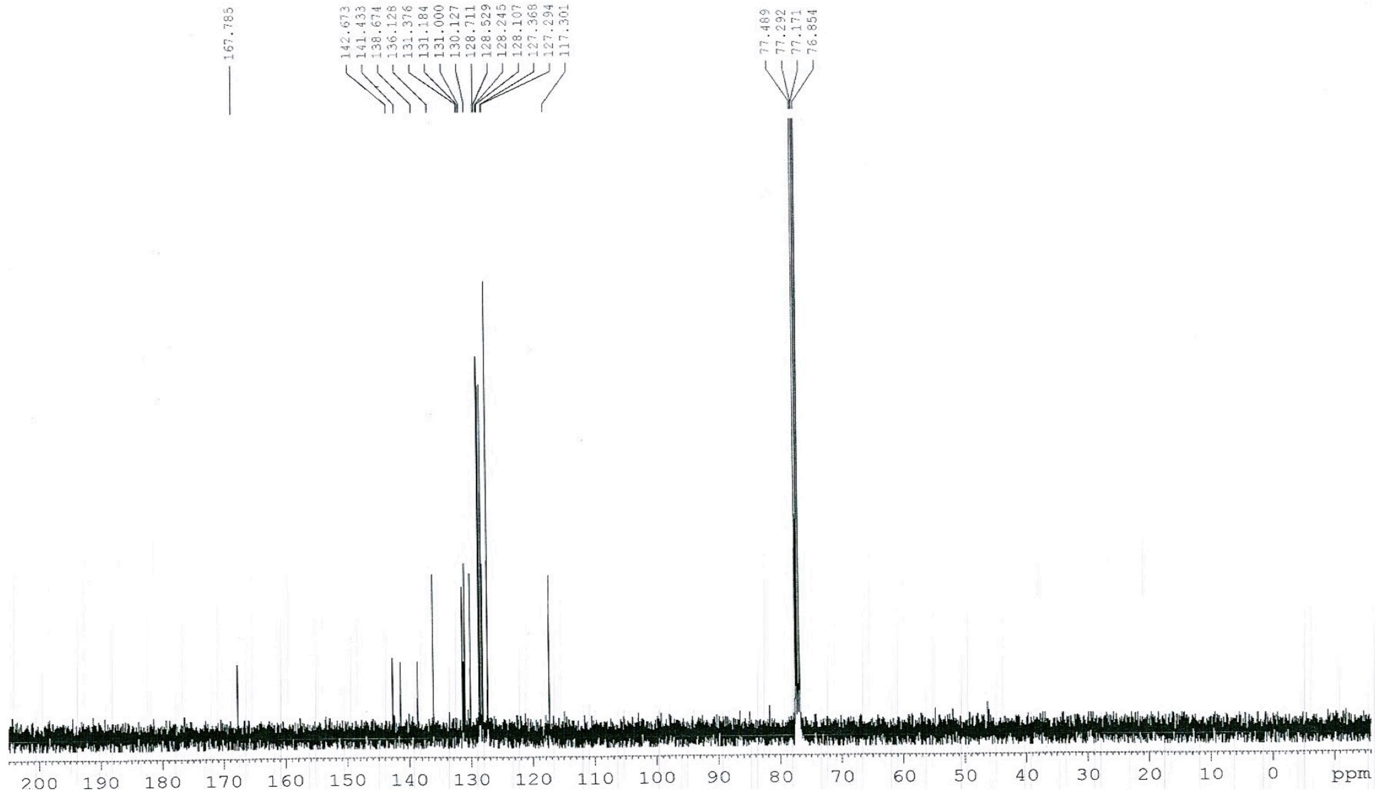

Figure S34 ^{13}C NMR (100MHz) spectrum of 1-phenylallyl biphenyl-2-carboxylate (7e) in $CDCl_3$.

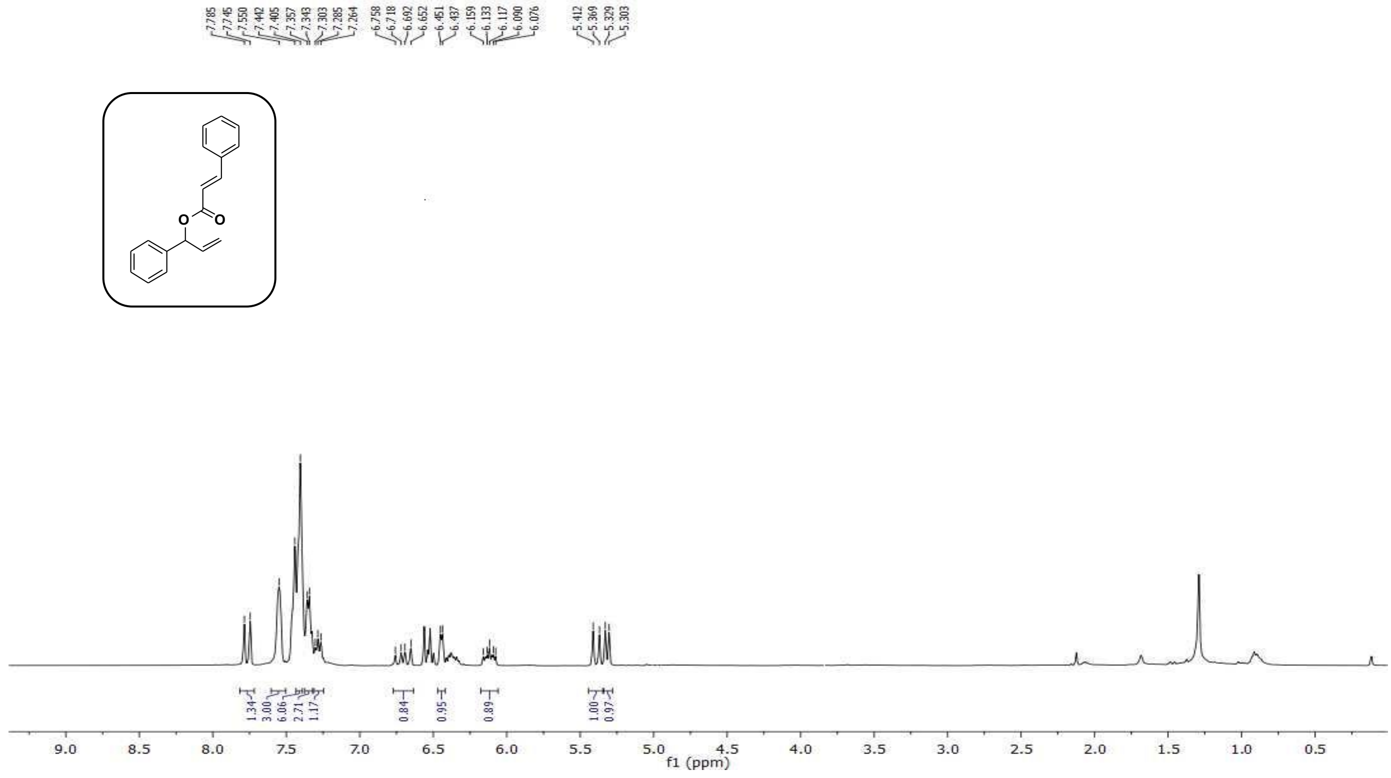

Figure S35 ^{1}H NMR (400MHz) spectrum of 1-Phenyl-2-propenyl cinnamate (7f) in $CDCl_3$.

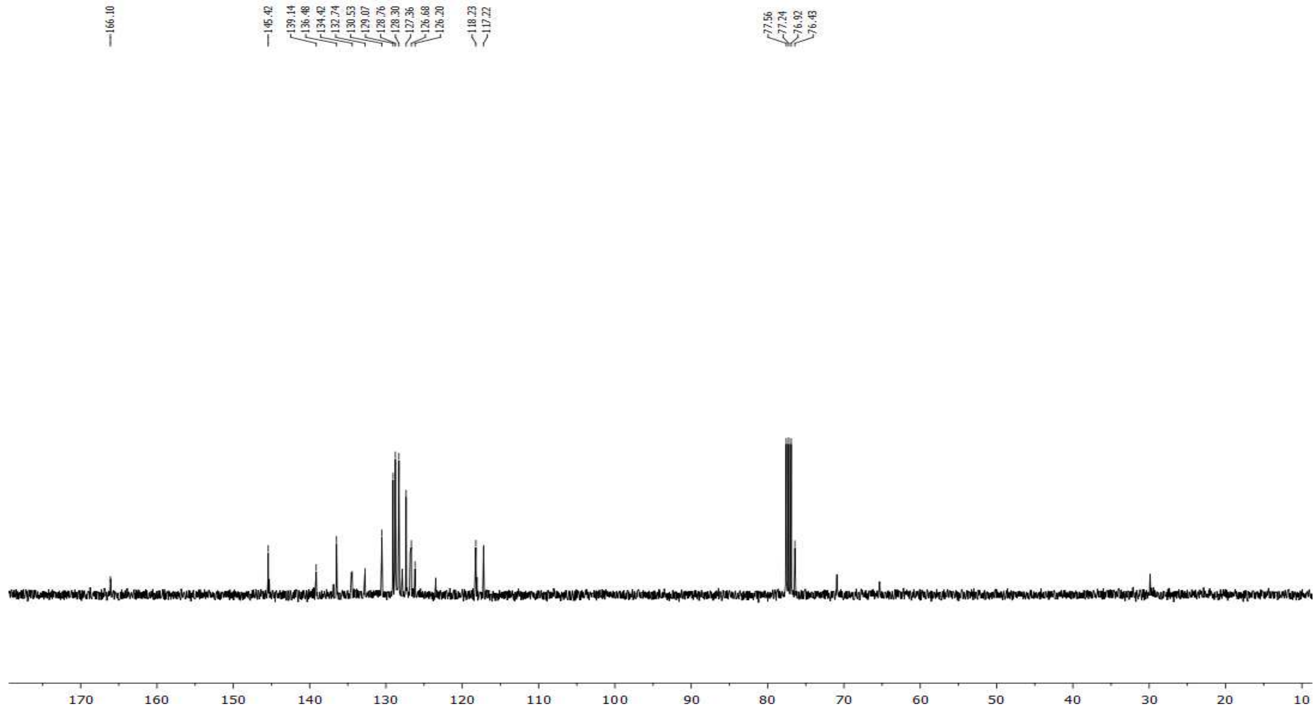

Figure S36 ^{13}C NMR (100MHz) spectrum of 1-Phenyl-2-propenyl cinnamate (7f) in $CDCl_3$.

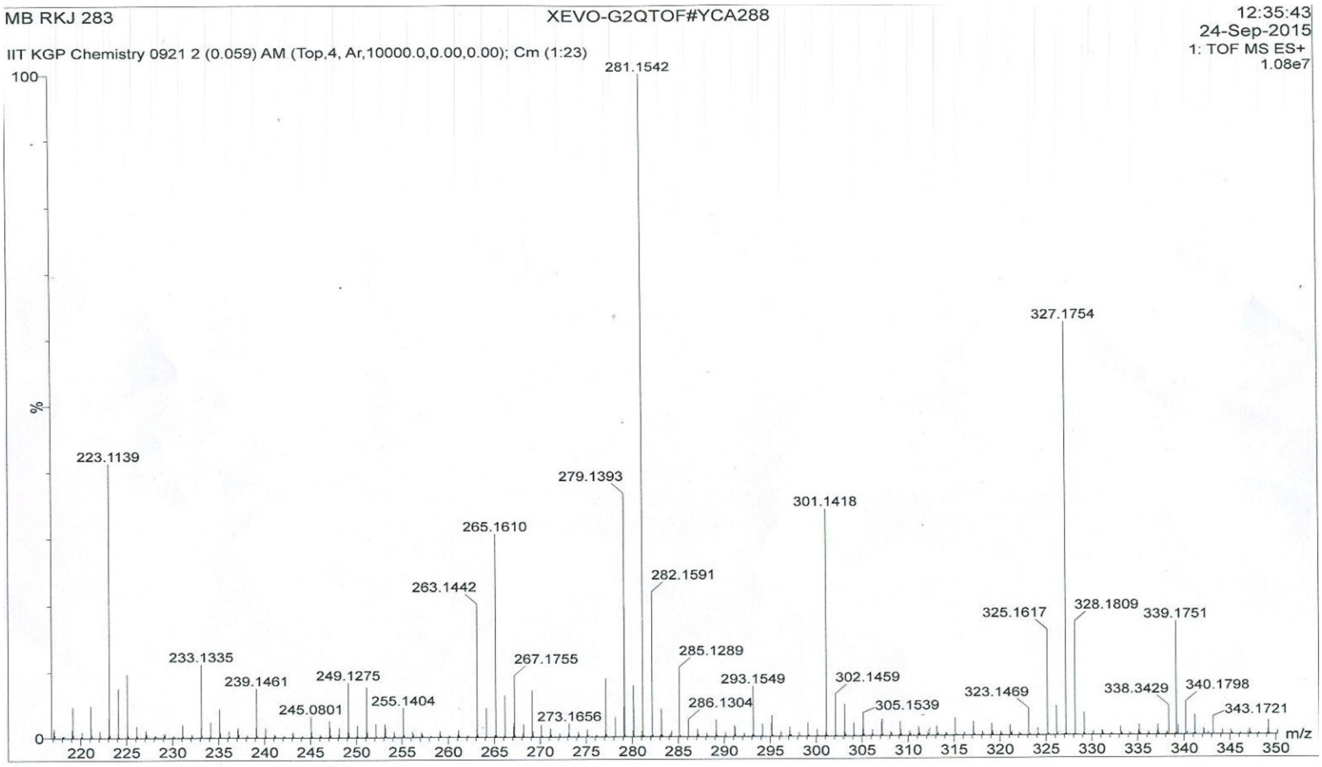

Figure S37 HRMS of 4f

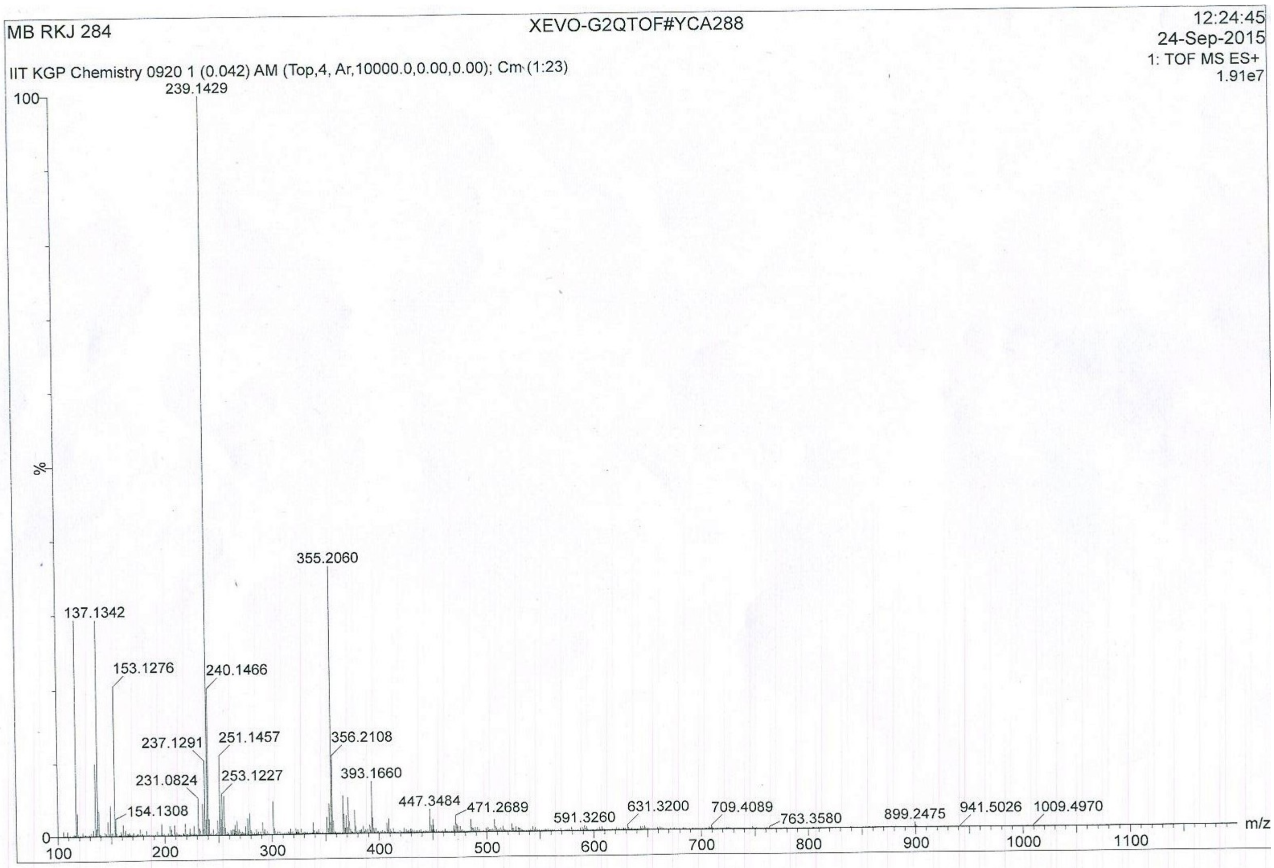

Figure S38 HRMS of 4g

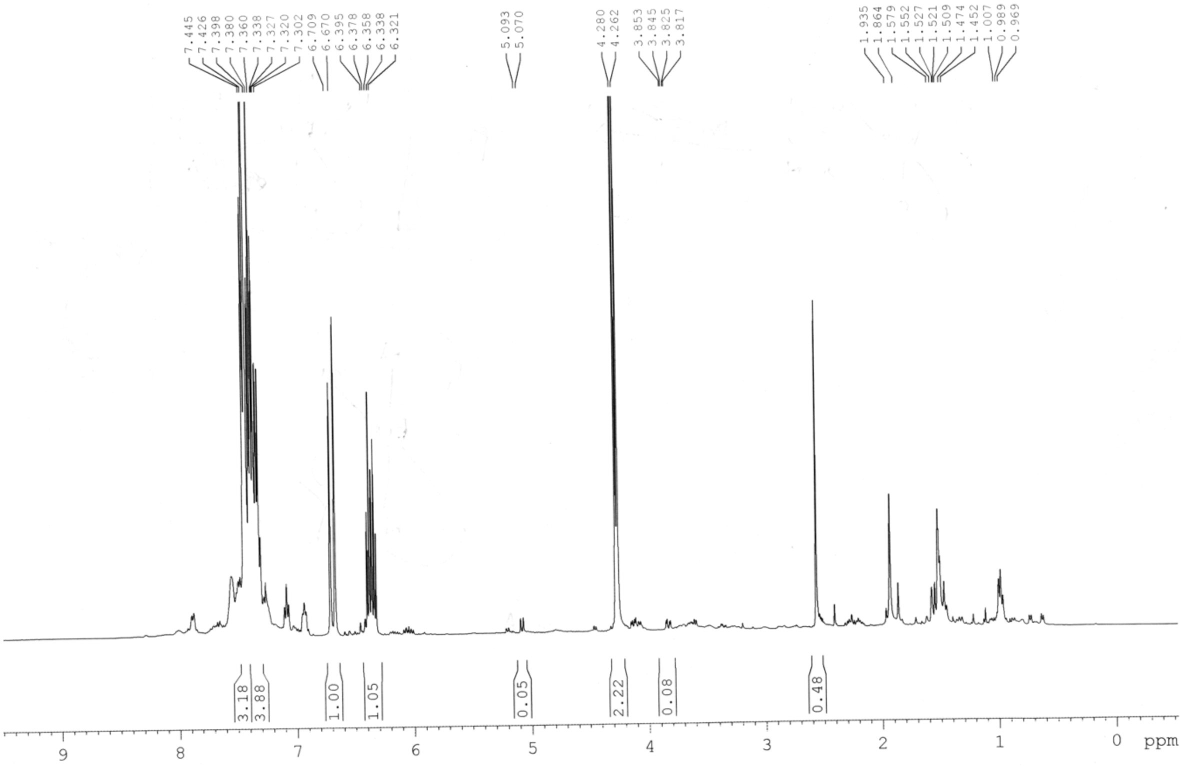

Figure S39 ^{1}H NMR (400 MHz) spectrum of **1** + cinnamyl alcohol (**2a**) in CD_3CN.

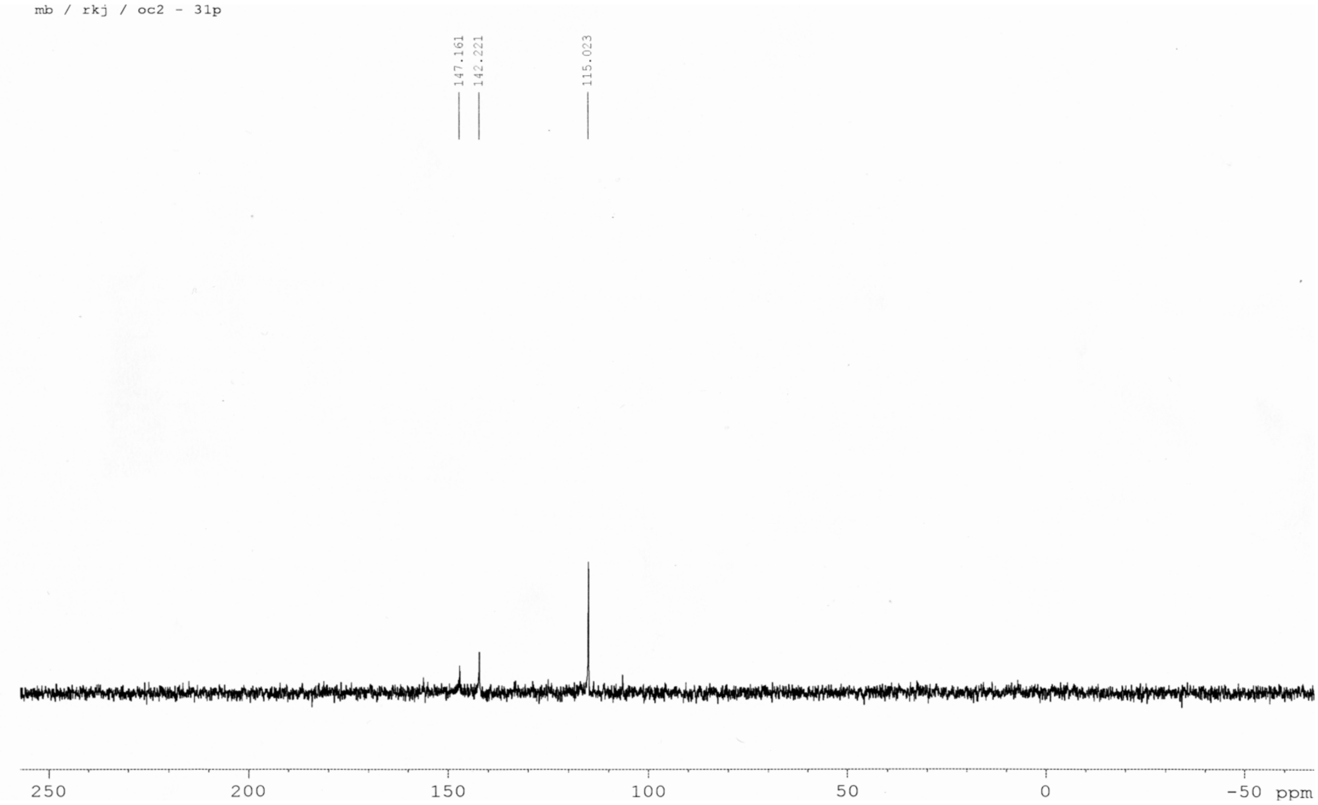

Figure S40 ^{13}P NMR (161.98 MHz) spectrum of **1** + cinnamyl alcohol (**2a**) in CD_3CN.

Sample Info : RKJ-1-2
CHIRALPAK IA-3, 250 MM, IPA/HEXANE : 10:90, 1.0 ml/min flow rate, 254

Additional Info : Peak(s) manually integrated

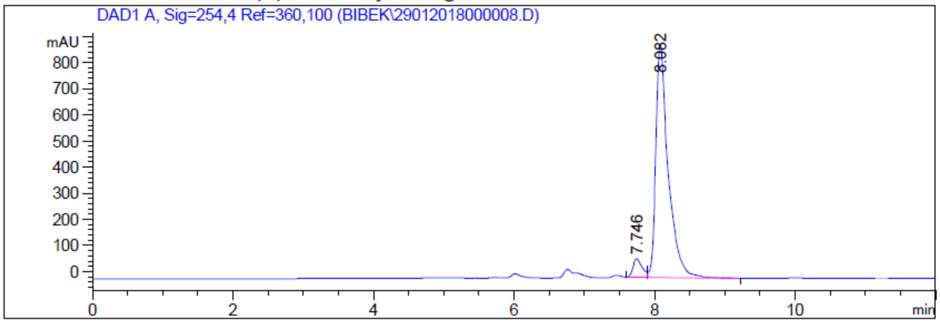

==
Area Percent Report
==

Sorted By : Signal
Multiplier : 1.0000
Dilution : 1.0000
Use Multiplier & Dilution Factor with ISTDs

Signal 1: DAD1 A, Sig=254,4 Ref=360,100

Peak #	RetTime [min]	Type	Width [min]	Area [mAU*s]	Height [mAU]	Area %
1	7.746	BV	0.1363	651.34174	71.45866	5.6403
2	8.082	VB	0.1764	1.08967e4	892.80103	94.3597

Totals : 1.15480e4 964.25969

Figure S41 Chiral HPLC of **4a**

```
Sample Info      : R J 383
                   CHIRALPAK IA-3 250MM , 10 % iPrOH/HEXANE 1.0 ml/min flow rate, 254 nm

Additional Info : Peak(s) manually integrated
```

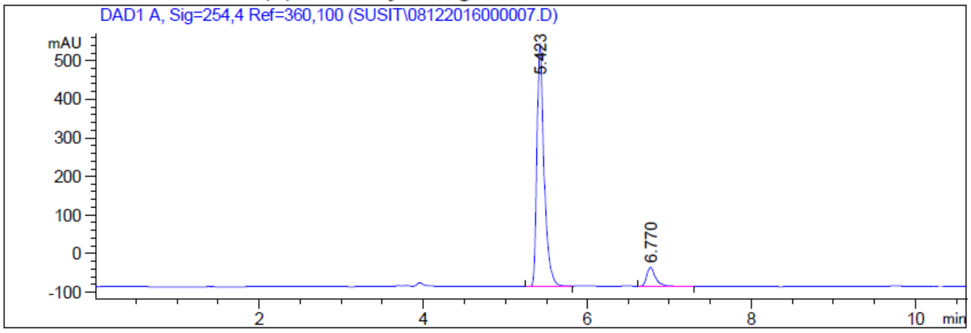


```
=====================================================================
                        Area Percent Report
=====================================================================

Sorted By             :      Signal
Multiplier            :      1.0000
Dilution              :      1.0000
Use Multiplier & Dilution Factor with ISTDs


Signal 1: DAD1 A, Sig=254,4 Ref=360,100
```

Peak #	RetTime [min]	Type	Width [min]	Area [mAU*s]	Height [mAU]	Area %
1	5.423	BV	0.0891	3806.06372	624.55359	90.9869
2	6.770	VB	0.1145	377.02780	49.70564	9.0131
Totals :				4183.09152	674.25923	

Figure S42 Chiral HPLC of **4b**

```
Sample Info     : RKJ-2
                  CHIRALPAK IA-3, 250 MM,   IPA/HEXANE : 10:90, 1.0 ml/min flow rate, 254

Additional Info : Peak(s) manually integrated
```

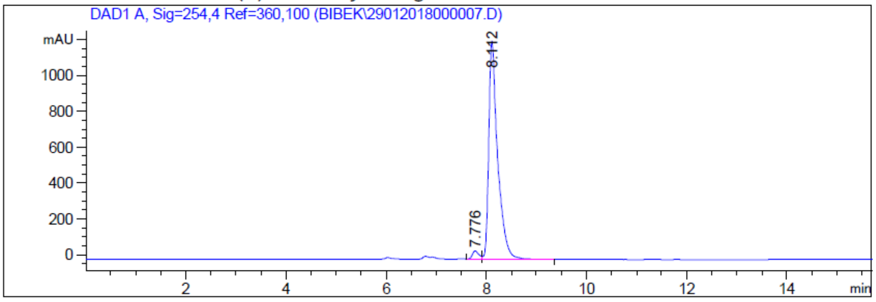


```
=====================================================================
                         Area Percent Report
=====================================================================

Sorted By             :      Signal
Multiplier            :      1.0000
Dilution              :      1.0000
Use Multiplier & Dilution Factor with ISTDs


Signal 1: DAD1 A, Sig=254,4 Ref=360,100

Peak RetTime Type  Width     Area      Height     Area
  #   [min]        [min]   [mAU*s]     [mAU]        %
----|-------|----|-------|----------|----------|--------|
   1   7.776 BV    0.1377  427.44101   46.29233   2.7700
   2   8.112 VB    0.1781 1.50035e4  1214.52356  97.2300

Totals :                  1.54310e4  1260.81589
```

Figure S43 Chiral HPLC of **4c**

Sample Info : R J 397
CHIRALPAK IA-3 250MM , 10 % iPrOH/HEXANE 1.0 ml/min flow rate, 254 nm

Additional Info : Peak(s) manually integrated

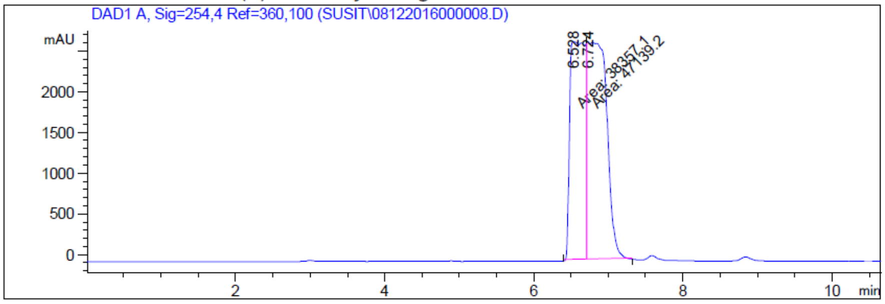

==
Area Percent Report
==

Sorted By : Signal
Multiplier : 1.0000
Dilution : 1.0000
Use Multiplier & Dilution Factor with ISTDs

Signal 1: DAD1 A, Sig=254,4 Ref=360,100

Peak #	RetTime [min]	Type	Width [min]	Area [mAU*s]	Height [mAU]	Area %
1	6.528	MF	0.2393	3.83571e4	2671.10107	44.8641
2	6.724	FM	0.2951	4.71392e4	2662.14160	55.1359

Totals : 8.54964e4 5333.24268

Figure S44 Chiral HPLC of **4d**

Sample Info   : RKJ-3
                   CHIRALPAK IA-3, 250 MM,   IPA/HEXANE : 10:90, 1.0 ml/min flc

Additional Info : Peak(s) manually integrated

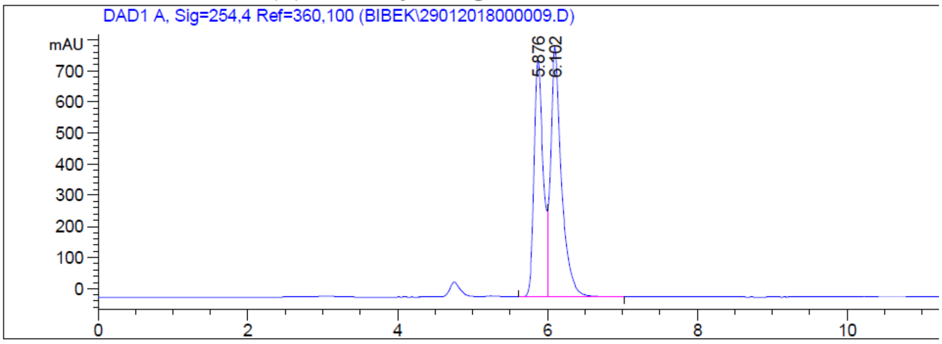

===

Area Percent Report

===

Sorted By : Signal
Multiplier : 1.0000
Dilution : 1.0000
Use Multiplier & Dilution Factor with ISTDs

Signal 1: DAD1 A, Sig=254,4 Ref=360,100

Peak #	RetTime [min]	Type	Width [min]	Area [mAU*s]	Height [mAU]	Area %
1	5.876	BV	0.1240	6263.58252	761.51666	43.3013
2	6.102	VB	0.1454	8201.54199	801.19952	56.6987

Totals : 1.44651e4   1562.71619

Figure S45 Chiral HPLC of **4g**

```
Sample Info     : R J 381
                  CHIRALPAK IA-3 250MM , 10 % iPrOH/HEXANE 1.0 ml/min flow rate, 254 nm

Additional Info : Peak(s) manually integrated
```

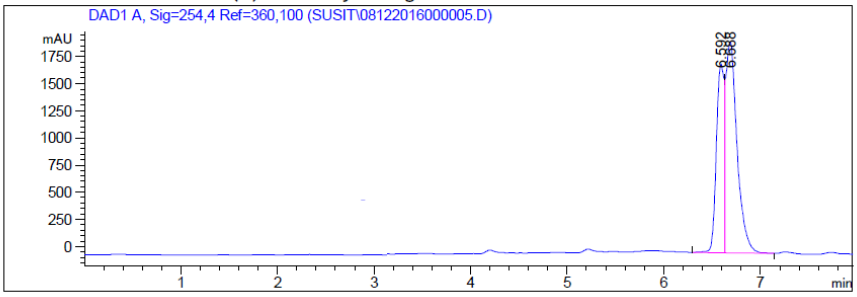


```
=====================================================================
                         Area Percent Report
=====================================================================

Sorted By             :      Signal
Multiplier            :      1.0000
Dilution              :      1.0000
Use Multiplier & Dilution Factor with ISTDs


Signal 1: DAD1 A, Sig=254,4 Ref=360,100
```

Peak #	RetTime [min]	Type	Width [min]	Area [mAU*s]	Height [mAU]	Area %
1	6.592	BV	0.0839	9504.08594	1736.27625	37.3413
2	6.688	VB	0.1198	1.59478e4	1942.84363	62.6587

Totals : 2.54519e4 3679.11987

Figure S46 Chiral HPLC of **7a**

DFT Study

Geometries in Cartesian coordinates (Angstroms), the structures of the following compounds:

C1a [Ru(AMP)$_2$(CH$_3$CN)$_2$][2BPh$_4$]

Ru	-0.070747000	-0.233097000	0.138207000
P	1.130728000	0.213420000	-1.910342000
C	1.520442000	-1.459141000	-4.151319000
H	0.922273000	-0.736128000	-4.694810000
C	2.596414000	-2.202177000	-2.118041000
H	2.816504000	-2.088917000	-1.059851000
C	3.754974000	0.933450000	-2.732005000
H	3.829313000	-0.050619000	-3.176087000
C	3.175584000	-3.265424000	-2.812046000
H	3.843742000	-3.948194000	-2.294020000
C	2.589021000	1.330576000	-2.049991000
C	2.911800000	-3.440879000	-4.174425000
H	3.362443000	-4.268077000	-4.714705000
C	1.757258000	-1.288755000	-2.778751000
C	4.768847000	3.096133000	-2.322951000
H	5.607652000	3.777003000	-2.433130000
C	2.540959000	2.626057000	-1.508271000
H	1.656632000	2.967697000	-0.990164000
C	4.833876000	1.810083000	-2.861138000
H	5.722487000	1.486203000	-3.394672000
C	1.596882000	1.871704000	2.769461000
C	-1.178606000	0.324377000	-3.272388000
C	2.083555000	-2.536970000	-4.840038000
H	1.887750000	-2.656327000	-5.901449000
C	3.615594000	3.503728000	-1.648951000
H	3.545888000	4.506917000	-1.238478000
H	-1.489812000	0.778944000	-4.214199000
H	-1.107157000	-0.755856000	-3.416503000
H	1.948863000	2.906424000	2.788672000
H	1.718214000	1.437915000	3.761847000
N	-2.076077000	-0.208346000	-0.957472000
O	0.121024000	0.903381000	-3.020279000
C	-2.198184000	0.673000000	-2.192581000
H	-1.918946000	1.655453000	-1.808798000
C	-3.659885000	0.795750000	-2.732801000
C	-4.075202000	-0.244489000	-3.792140000
C	-3.877794000	2.215173000	-3.291549000
H	-4.322723000	0.680567000	-1.863261000
H	-3.915129000	-1.276246000	-3.475207000
H	-5.139419000	-0.123102000	-4.018260000
H	-3.533378000	-0.095945000	-4.733176000
H	-3.699502000	2.989659000	-2.536224000
H	-3.217424000	2.419683000	-4.143077000
H	-4.906566000	2.327916000	-3.647130000

C	-2.652537000	-1.539590000	-1.111170000
O	-2.286249000	-2.320945000	-1.959007000
O	-3.598268000	-1.697769000	-0.193917000
C	-4.439781000	-2.942486000	-0.090404000
C	-5.240928000	-3.123852000	-1.379127000
C	-3.542604000	-4.141803000	0.212907000
C	-5.352910000	-2.612671000	1.089178000
H	-5.831798000	-2.229482000	-1.600520000
H	-4.594234000	-3.348976000	-2.229333000
H	-5.936577000	-3.959597000	-1.250832000
H	-2.905872000	-3.942297000	1.082064000
H	-4.170214000	-5.008055000	0.445863000
H	-2.916812000	-4.395790000	-0.644418000
H	-6.047683000	-3.440261000	1.261033000
H	-4.773065000	-2.456289000	2.004807000
H	-5.937482000	-1.709722000	0.889764000
H	-2.638489000	0.247898000	-0.235448000
C	-1.606993000	-1.293446000	2.835716000
N	-1.139571000	-0.899379000	1.853837000
C	-2.176179000	-1.751903000	4.095506000
H	-1.430618000	-1.639671000	4.889581000
H	-3.056708000	-1.150033000	4.341779000
H	-2.469536000	-2.803855000	4.026428000
C	0.154653000	-3.412015000	-0.547759000
N	0.162890000	-2.291894000	-0.258255000
C	0.158973000	-4.802272000	-0.980944000
H	0.046281000	-5.478386000	-0.128128000
H	-0.667551000	-4.962252000	-1.680602000
H	1.101314000	-5.017822000	-1.494991000
N	1.872800000	-0.228491000	1.341821000
P	-0.562708000	1.941023000	1.093483000
C	-2.298600000	2.067594000	1.705692000
C	-3.321171000	2.455491000	0.824277000
C	-2.614766000	1.827530000	3.052433000
C	-4.639752000	2.568731000	1.271385000
H	-3.091072000	2.732227000	-0.200989000
C	-3.931722000	1.958052000	3.497815000
H	-1.825395000	1.578271000	3.752413000
C	-4.948453000	2.315857000	2.608766000
H	-5.417321000	2.883294000	0.581482000
H	-4.161305000	1.800696000	4.548284000
H	-5.969743000	2.421974000	2.961955000
C	-0.314254000	3.658846000	0.485455000
C	-0.114197000	4.674313000	1.438304000
C	-0.448613000	4.005084000	-0.868338000
C	-0.031469000	6.006838000	1.036445000
H	-0.042871000	4.424458000	2.492320000
C	-0.380034000	5.343191000	-1.263245000
H	-0.574997000	3.238813000	-1.626839000

C	-0.168151000	6.343741000	-0.313148000
H	0.129008000	6.783338000	1.778440000
H	-0.483899000	5.600382000	-2.313189000
H	-0.111429000	7.383201000	-0.622071000
C	1.884219000	-1.209150000	2.419984000
O	1.412620000	-0.986017000	3.509969000
O	2.485655000	-2.314359000	1.991153000
C	2.750050000	-3.482142000	2.907971000
C	3.654011000	-3.025003000	4.052937000
C	1.421444000	-4.056229000	3.399524000
C	3.475802000	-4.459477000	1.985106000
H	4.572985000	-2.573603000	3.665015000
H	3.149602000	-2.310946000	4.706538000
H	3.938028000	-3.895745000	4.652873000
H	0.766450000	-4.300740000	2.555792000
H	1.614667000	-4.979957000	3.954606000
H	0.908143000	-3.359231000	4.064173000
H	3.736100000	-5.365685000	2.540170000
H	2.842878000	-4.747507000	1.138895000
H	4.400334000	-4.020479000	1.597744000
H	2.504329000	-0.592336000	0.631452000
C	2.447115000	1.120222000	1.739010000
H	2.446242000	1.679082000	0.799943000
O	0.178263000	1.895470000	2.555621000
C	3.922828000	1.008311000	2.225358000
C	4.519260000	2.399077000	2.521482000
C	4.818906000	0.276014000	1.208424000
H	3.923591000	0.435914000	3.164044000
H	4.039198000	2.899922000	3.366974000
H	5.579605000	2.297505000	2.771653000
H	4.449553000	3.054807000	1.644764000
H	4.541849000	-0.775259000	1.069704000
H	4.808681000	0.774059000	0.231528000
H	5.853910000	0.277191000	1.564185000

C1b [Ru(AMP)$_2$(CH$_3$CN)$_2$][2BPh$_4$]

Ru	-0.026605000	0.238970000	-0.033867000
P	-1.916686000	1.578132000	0.290005000
P	-0.943139000	-1.902413000	0.531053000
C	-2.815334000	-3.796193000	-0.422477000
H	-3.285289000	-3.725280000	0.551308000
C	-1.603809000	5.958734000	-1.239084000
H	-1.562386000	6.979392000	-1.607711000
C	-1.732210000	3.317169000	-0.291939000
C	-1.093668000	-3.141846000	-2.000946000
H	-0.209432000	-2.571192000	-2.261062000
C	-3.243887000	-1.510306000	2.126512000
H	-3.618660000	-0.884172000	1.326173000

C	-1.615886000	-4.039347000	-2.933619000
H	-1.131284000	-4.152092000	-3.899677000
C	-1.985144000	-2.129116000	2.027401000
C	-1.371152000	3.579686000	-1.623673000
H	-1.140629000	2.760670000	-2.297859000
C	-2.743703000	-4.803243000	-2.623178000
H	-3.148777000	-5.499001000	-3.352079000
C	-1.964221000	5.706475000	0.084841000
H	-2.203400000	6.529764000	0.751430000
C	-1.686851000	-3.013649000	-0.728730000
C	-3.586544000	-2.512739000	4.303739000
H	-4.205992000	-2.661659000	5.183139000
C	-1.542614000	-2.952001000	3.079276000
H	-0.585159000	-3.453625000	3.009879000
C	-0.494050000	0.730492000	3.185992000
C	-5.045551000	-0.319991000	-1.644477000
H	-5.197467000	-1.214064000	-2.242193000
C	-3.770187000	-0.022660000	-1.157407000
H	-2.951146000	-0.696697000	-1.374940000
C	-3.560004000	1.121733000	-0.374220000
C	-2.034841000	4.392884000	0.557127000
H	-2.347621000	4.212068000	1.579803000
C	-1.312053000	4.891236000	-2.094820000
H	-1.050252000	5.081857000	-3.132031000
C	-6.120325000	0.520165000	-1.351397000
H	-7.111808000	0.288331000	-1.728863000
C	-5.919652000	1.661243000	-0.568809000
H	-6.753749000	2.316468000	-0.336461000
C	-4.038106000	-1.705638000	3.256657000
H	-5.010829000	-1.226659000	3.316154000
C	-4.648601000	1.966734000	-0.087408000
H	-4.505903000	2.860167000	0.511292000
C	-1.281290000	1.987940000	2.863206000
C	1.493192000	-3.140410000	0.282222000
C	-3.341076000	-4.677802000	-1.367984000
H	-4.212602000	-5.274570000	-1.116026000
C	-2.340168000	-3.134536000	4.210879000
H	-1.988287000	-3.773803000	5.015239000
H	1.845786000	-4.048026000	0.770638000
H	1.157386000	-3.403011000	-0.721861000
H	-1.172749000	-0.118550000	3.250825000
H	-0.013619000	0.857559000	4.163271000
H	-1.824389000	2.290498000	3.762193000
H	-0.622783000	2.806006000	2.562417000
N	2.181486000	-0.852325000	-0.483880000
O	0.400195000	-2.691901000	1.103882000
C	2.616716000	-2.107808000	0.252888000
H	2.769015000	-1.764711000	1.281815000
C	3.965800000	-2.694231000	-0.258714000

C	4.392074000	-3.929790000	0.559862000
C	5.098404000	-1.651862000	-0.217975000
H	3.823558000	-3.012870000	-1.298609000
H	3.756473000	-4.802064000	0.383646000
H	5.410387000	-4.219841000	0.283446000
H	4.393432000	-3.717829000	1.636770000
H	4.881228000	-0.750061000	-0.798280000
H	5.318825000	-1.352343000	0.813652000
H	6.015684000	-2.085373000	-0.628776000
C	2.350962000	-0.911191000	-1.912576000
O	1.910222000	-1.819549000	-2.585057000
O	3.038278000	0.149986000	-2.330490000
C	3.503815000	0.297271000	-3.754009000
C	4.416921000	-0.875163000	-4.114220000
C	2.296769000	0.403405000	-4.684925000
C	4.282081000	1.611301000	-3.705248000
H	5.253527000	-0.948719000	-3.412151000
H	3.873691000	-1.821937000	-4.128118000
H	4.834232000	-0.707297000	-5.112410000
H	1.633033000	1.213059000	-4.360974000
H	2.644939000	0.637356000	-5.696331000
H	1.743283000	-0.536821000	-4.721271000
H	4.698298000	1.830720000	-4.692980000
H	3.628327000	2.442905000	-3.420257000
H	5.110245000	1.550567000	-2.992549000
H	2.750404000	-0.078663000	-0.146372000
N	0.572132000	0.367341000	2.174943000
O	-2.304691000	1.754962000	1.877861000
C	1.762748000	1.166503000	2.468256000
O	1.747948000	2.374029000	2.433769000
O	2.762515000	0.362883000	2.807534000
C	4.029331000	0.903667000	3.454575000
C	4.798729000	1.727261000	2.424564000
C	3.650654000	1.707580000	4.698300000
C	4.768323000	-0.375702000	3.837640000
H	5.000231000	1.139269000	1.522605000
H	4.255322000	2.635010000	2.154204000
H	5.763259000	2.021930000	2.850598000
H	3.045120000	1.103465000	5.382369000
H	4.568238000	1.983982000	5.227574000
H	3.113776000	2.624927000	4.451241000
H	5.710293000	-0.116922000	4.330545000
H	4.175112000	-0.978346000	4.532040000
H	5.002463000	-0.982048000	2.957927000
H	0.824305000	-0.605695000	2.362231000
C	1.715252000	2.969789000	-0.568999000
N	1.125012000	1.982838000	-0.440041000
C	2.416101000	4.239089000	-0.699030000
H	1.736882000	4.982744000	-1.129029000

H	2.737437000	4.579233000	0.290713000
H	3.291630000	4.131769000	-1.346602000
C	-0.904990000	0.018361000	-3.101630000
N	-0.539273000	0.096705000	-2.004813000
C	-1.423812000	-0.113166000	-4.457157000
H	-0.728915000	0.323093000	-5.180399000
H	-1.564907000	-1.173677000	-4.690913000
H	-2.389856000	0.397165000	-4.533228000

C1c [Ru(AMP)$_2$(CH$_3$CN)$_2$][2BPh$_4$]

Ru	0.058053000	0.220910000	-0.206544000
P	-1.340089000	2.213826000	0.124899000
C	-0.786518000	5.947965000	-2.565745000
H	-0.687529000	6.825642000	-3.197411000
C	-1.053224000	3.683150000	-0.934358000
C	-0.892005000	3.541971000	-2.322192000
H	-0.874436000	2.554049000	-2.773457000
C	-0.944366000	6.093673000	-1.186134000
H	-0.969756000	7.084473000	-0.742494000
C	0.726456000	1.631788000	2.673283000
C	-5.227061000	1.917417000	-1.188033000
H	-5.732436000	1.857971000	-2.147817000
C	-3.833863000	1.943338000	-1.138233000
H	-3.270041000	1.926471000	-2.066379000
C	-3.170079000	2.057510000	0.094936000
C	-1.087584000	4.968750000	-0.372260000
H	-1.244053000	5.092550000	0.694462000
C	-0.769172000	4.671197000	-3.134126000
H	-0.668150000	4.556365000	-4.210024000
C	-5.973239000	2.008214000	-0.008719000
H	-7.058447000	2.012668000	-0.050944000
C	-5.319250000	2.114526000	1.219510000
H	-5.893772000	2.201555000	2.137131000
C	-3.923147000	2.135083000	1.276309000
H	-3.425933000	2.259177000	2.231665000
C	0.081524000	2.954481000	2.289175000
C	0.298307000	-2.896007000	1.838668000
H	0.689191000	-3.457967000	2.685421000
H	-0.262988000	-3.576548000	1.197213000
H	-0.033085000	0.979981000	3.108460000
H	1.483948000	1.817245000	3.445350000
H	-0.124000000	3.509611000	3.208306000
H	0.741693000	3.557489000	1.661294000
N	1.385912000	0.882198000	1.536628000
O	-1.200743000	2.784100000	1.668773000
C	2.636814000	1.617369000	1.241432000
O	2.633927000	2.653796000	0.621134000
O	3.639464000	1.005039000	1.838421000

C	5.013326000	1.648305000	1.965658000
C	5.605968000	1.869010000	0.576363000
C	4.860930000	2.943681000	2.761051000
C	5.785806000	0.593771000	2.752667000
H	5.605265000	0.940688000	-0.001814000
H	5.064744000	2.642487000	0.027887000
H	6.645176000	2.196285000	0.687774000
H	4.398449000	2.752981000	3.735565000
H	5.854902000	3.364258000	2.944662000
H	4.275615000	3.689447000	2.218601000
H	6.804528000	0.950054000	2.933425000
H	5.314621000	0.403511000	3.721982000
H	5.841842000	-0.345256000	2.195796000
H	1.697121000	-0.006529000	1.935472000
C	1.925105000	1.924424000	-2.150516000
N	1.242671000	1.262980000	-1.490501000
C	2.779903000	2.801784000	-2.937882000
H	2.179411000	3.616313000	-3.355959000
H	3.549144000	3.228855000	-2.286495000
H	3.261136000	2.251148000	-3.751915000
C	-1.722063000	-0.687764000	-2.707058000
N	-1.103150000	-0.346912000	-1.789407000
C	-2.475827000	-1.156896000	-3.863457000
H	-2.215759000	-0.570343000	-4.750551000
H	-2.236033000	-2.209472000	-4.047544000
H	-3.550076000	-1.063332000	-3.678699000
N	-1.276630000	-0.997426000	1.240873000
P	1.456744000	-1.788272000	-0.363699000
C	-0.588085000	-1.767502000	2.372482000
H	0.069907000	-1.036054000	2.848598000
O	1.480938000	-2.428043000	1.164176000
C	3.270545000	-1.649126000	-0.618716000
C	3.744275000	-1.112582000	-1.827377000
C	4.180963000	-2.208263000	0.290155000
C	5.107212000	-1.136672000	-2.122926000
H	3.049676000	-0.710238000	-2.559300000
C	5.544590000	-2.231310000	-0.012413000
H	3.824463000	-2.644230000	1.216122000
C	6.010448000	-1.697908000	-1.215880000
H	5.463600000	-0.735522000	-3.067392000
H	6.241114000	-2.684257000	0.687455000
H	7.069713000	-1.731048000	-1.452933000
C	1.068724000	-3.184906000	-1.487870000
C	1.202587000	-4.510183000	-1.048775000
C	0.762382000	-2.933164000	-2.833528000
C	0.997550000	-5.566725000	-1.937013000
H	1.486309000	-4.722030000	-0.022862000
C	0.577214000	-3.993113000	-3.722592000
H	0.693069000	-1.912454000	-3.197708000

C	0.682023000	-5.311489000	-3.272805000
H	1.099002000	-6.589828000	-1.587360000
H	0.362452000	-3.791812000	-4.768675000
H	0.533932000	-6.135742000	-3.963943000
C	-2.233637000	-1.874302000	0.543191000
O	-1.858217000	-2.747532000	-0.203177000
O	-3.469500000	-1.569131000	0.896697000
C	-4.656299000	-2.406836000	0.454314000
C	-4.815217000	-2.271012000	-1.058215000
C	-4.454734000	-3.855291000	0.900083000
C	-5.813868000	-1.754719000	1.204410000
H	-4.889549000	-1.215155000	-1.339708000
H	-3.983550000	-2.741992000	-1.587054000
H	-5.740529000	-2.769377000	-1.365278000
H	-4.303162000	-3.913680000	1.983182000
H	-5.362731000	-4.420895000	0.666670000
H	-3.616554000	-4.331791000	0.389156000
H	-6.742781000	-2.280681000	0.962983000
H	-5.662441000	-1.814202000	2.286658000
H	-5.926023000	-0.705317000	0.921424000
H	-1.844266000	-0.283712000	1.698259000
C	-1.593015000	-2.245716000	3.464442000
C	-2.424242000	-1.076067000	4.026170000
C	-0.864944000	-2.950128000	4.627797000
H	-2.281267000	-2.970290000	3.010073000
H	-3.072608000	-0.604721000	3.281254000
H	-3.080263000	-1.439630000	4.822846000
H	-1.777502000	-0.308456000	4.472353000
H	-0.450651000	-3.923053000	4.350276000
H	-0.051225000	-2.330816000	5.026499000
H	-1.568417000	-3.128287000	5.446409000

CH_3CN

C	0.000259000	0.017402000	1.180819000
H	0.078827000	1.046385000	1.545833000
H	-0.925284000	-0.420704000	1.567674000
H	0.847443000	-0.556658000	1.569332000
C	-0.000058000	-0.004130000	-0.280481000
N	-0.000313000	-0.021237000	-1.440695000

Ligand (L)used for calculation [$C_7H_{16}NO_3P$]

C	0.741257000	-1.240135000	0.288941000
H	0.788027000	-2.316544000	0.492835000
H	0.769124000	-0.697855000	1.241493000
N	-0.555716000	0.517657000	-0.862938000
P	3.352588000	-0.804433000	0.175696000
C	-0.529172000	1.529099000	0.056861000

O	-0.768842000	1.431095000	1.249234000
O	-0.222369000	2.704248000	-0.560136000
H	-0.109424000	0.742897000	-1.741368000
C	-0.543596000	-0.901513000	-0.478660000
H	-0.502282000	-1.448010000	-1.429773000
O	1.842290000	-0.858639000	-0.549859000
C	-1.844054000	-1.324388000	0.251834000
C	-1.831903000	-2.817986000	0.619553000
C	-3.076083000	-1.005299000	-0.608478000
H	-1.907087000	-0.737124000	1.173854000
H	-1.074775000	-3.062922000	1.371378000
H	-2.803782000	-3.110593000	1.032582000
H	-1.647721000	-3.445280000	-0.263253000
H	-3.116133000	0.053071000	-0.879212000
H	-3.071159000	-1.591653000	-1.537426000
H	-3.994824000	-1.253219000	-0.065021000
C	-0.218443000	3.848786000	0.301201000
H	0.046226000	4.692313000	-0.338001000
H	-1.204759000	4.003556000	0.747445000
H	0.516919000	3.730934000	1.101942000
H	3.974700000	-1.945495000	-0.433159000
H	3.941487000	0.083803000	-0.770611000

Reactant (2a)

C	1.725635000	0.519402000	-0.311101000
C	-0.530637000	0.187212000	0.783255000
C	0.493110000	0.945126000	0.008283000
H	2.356203000	1.213639000	-0.867926000
C	2.364728000	-0.804905000	-0.002952000
H	2.692383000	-1.292183000	-0.930889000
H	3.263394000	-0.667650000	0.613815000
H	1.699160000	-1.497400000	0.519621000
H	-0.154820000	-0.744143000	1.204369000
H	0.185238000	1.937411000	-0.317767000
Cl	-1.964347000	-0.283766000	-0.274541000
H	-0.964672000	0.793343000	1.581061000

Product (4a)

C	1.837177000	-0.809798000	-0.034402000
C	3.143298000	1.335416000	-0.436544000
C	2.330894000	0.363413000	-0.852923000
C	1.967063000	-0.679968000	1.483780000
H	1.419337000	0.180030000	1.877572000
H	1.578496000	-1.585324000	1.959293000
H	3.020673000	-0.575060000	1.762670000
H	2.007001000	0.338366000	-1.893265000
O	0.518101000	-1.229363000	-0.441492000

C	-0.605352000	-0.479732000	-0.201133000
C	-1.814469000	-1.174624000	-0.356750000
C	-0.628509000	0.878320000	0.141272000
C	-3.028335000	-0.520893000	-0.172086000
H	-1.773401000	-2.226209000	-0.623725000
C	-1.856721000	1.519832000	0.329169000
H	0.292124000	1.438858000	0.240619000
C	-3.059368000	0.832961000	0.176282000
H	-3.955881000	-1.073767000	-0.297410000
H	-1.862225000	2.574116000	0.594668000
H	-4.007341000	1.342205000	0.323952000
H	3.498740000	1.402844000	0.588327000
H	3.489743000	2.108043000	-1.117354000
H	2.433853000	-1.678770000	-0.343391000

Intermediate (1)

Ru	0.008595000	-0.051571000	-0.005162000
P	0.461600000	-1.230370000	-1.916383000
C	-3.007038000	-1.976039000	1.194180000
C	2.999586000	-1.807439000	-1.322445000
H	3.639987000	-2.686915000	-1.401221000
H	3.370550000	-1.041368000	-2.005368000
H	-3.322694000	-3.018472000	1.111984000
H	-3.713401000	-1.437581000	1.824236000
N	2.230048000	-0.032287000	0.344400000
O	1.704592000	-2.273958000	-1.765632000
C	3.002812000	-1.337473000	0.132629000
H	2.420373000	-2.065102000	0.707239000
C	4.428275000	-1.293739000	0.763925000
C	5.524972000	-0.682686000	-0.128474000
C	4.837509000	-2.706216000	1.223619000
H	4.342892000	-0.673510000	1.668926000
H	5.296381000	0.329422000	-0.474009000
H	6.460227000	-0.629997000	0.436683000
H	5.718799000	-1.300443000	-1.012338000
H	4.111060000	-3.136290000	1.922866000
H	4.948744000	-3.396463000	0.379641000
H	5.803636000	-2.665374000	1.735038000
C	2.823618000	1.126916000	-0.303926000
O	2.966753000	1.218141000	-1.498942000
O	3.145014000	2.028758000	0.616817000
H	2.267545000	0.156325000	1.347703000
C	-0.440023000	1.534912000	2.875612000
N	-0.198785000	1.040453000	1.855881000
C	-0.781578000	2.148342000	4.151717000
H	-1.871649000	2.161728000	4.258025000
H	-0.350919000	1.570104000	4.975607000
H	-0.399317000	3.173030000	4.196733000

C	0.073705000	2.707907000	-1.843386000
N	0.044700000	1.781256000	-1.148727000
C	0.130045000	3.863555000	-2.730294000
H	0.026959000	4.789197000	-2.155073000
H	1.089395000	3.873448000	-3.258505000
H	-0.678576000	3.811976000	-3.466765000
N	-2.245542000	-0.048451000	-0.307105000
P	-0.305934000	-1.955176000	1.244675000
C	-2.899574000	1.015038000	0.438328000
O	-3.225285000	0.924882000	1.594920000
O	-3.068960000	2.069236000	-0.359507000
C	-3.744300000	3.212598000	0.234072000
H	-2.287223000	0.236142000	-1.285033000
C	-2.968649000	-1.387104000	-0.223915000
H	-2.368804000	-2.044299000	-0.865343000
O	-1.769322000	-1.964137000	1.937153000
C	-4.397694000	-1.309318000	-0.835799000
C	-5.102148000	-2.680305000	-0.791735000
C	-4.370642000	-0.785824000	-2.284336000
H	-4.983299000	-0.609937000	-0.222027000
H	-5.314021000	-3.017872000	0.226488000
H	-6.062209000	-2.614707000	-1.311672000
H	-4.508861000	-3.452944000	-1.296910000
H	-4.031976000	0.254085000	-2.366588000
H	-3.741448000	-1.416327000	-2.926405000
H	-5.380300000	-0.809408000	-2.704422000
H	-0.481526000	-2.130607000	-2.452991000
H	0.747667000	-0.446396000	-3.058727000
H	-0.127045000	-3.228871000	0.647349000
H	0.474320000	-2.127100000	2.405343000
H	-3.115411000	3.651469000	1.011241000
H	-3.893714000	3.911763000	-0.586575000
H	-4.699424000	2.900566000	0.658802000
C	3.773901000	3.249097000	0.136585000
H	3.988939000	3.827853000	1.032596000
H	4.691665000	3.005419000	-0.401049000
H	3.087584000	3.786256000	-0.520495000

Intermediate (2)

C	1.902707000	-0.220498000	2.152902000
H	2.732594000	0.141984000	2.758436000
H	1.806469000	-1.298828000	2.291510000
N	1.057101000	-0.309873000	-0.256465000
P	-0.339607000	1.308247000	1.982334000
C	0.885381000	-1.774565000	-0.295179000
O	0.322353000	-2.359147000	0.599994000
O	1.390921000	-2.262514000	-1.405699000
H	1.363292000	-0.022764000	-1.189827000

C	2.177127000	0.152980000	0.694334000
H	2.166469000	1.244555000	0.586335000
O	0.716735000	0.365019000	2.752087000
C	3.568739000	-0.355446000	0.203608000
C	4.698587000	0.122035000	1.140894000
C	3.865395000	0.111654000	-1.235394000
H	3.557346000	-1.454391000	0.221635000
H	4.675380000	-0.357917000	2.123212000
H	5.665438000	-0.125126000	0.692742000
H	4.674058000	1.210126000	1.282785000
H	3.180618000	-0.298029000	-1.987461000
H	3.858014000	1.207500000	-1.305224000
H	4.865145000	-0.221246000	-1.529691000
C	-3.157193000	-1.359006000	-1.166884000
C	-2.798694000	-1.178642000	0.277785000
H	-3.170696000	-2.404269000	-1.477977000
H	-4.083517000	-0.863989000	-1.458436000
Ru	-0.900030000	0.510884000	-0.066144000
C	-3.613693000	-0.514692000	1.145377000
Cl	-1.836012000	-0.574827000	-2.248090000
C	1.287043000	-3.713021000	-1.612762000
H	1.782089000	-3.888870000	-2.565225000
H	1.793562000	-4.230449000	-0.797070000
H	0.233912000	-3.994267000	-1.654042000
H	-1.342249000	1.390817000	2.965053000
H	0.181875000	2.623689000	1.995810000
H	-2.018912000	-1.833801000	0.680387000
N	-0.256960000	2.178331000	-0.977836000
C	-0.021289000	3.154176000	-1.560721000
C	0.251143000	4.380703000	-2.298263000
H	1.186718000	4.832397000	-1.950850000
H	0.334641000	4.160356000	-3.368420000
H	-0.567465000	5.093152000	-2.144662000
C	-3.528824000	-0.563990000	2.633244000
H	-3.610364000	0.435257000	3.079391000
H	-4.402492000	-1.118572000	3.007182000
H	-2.629850000	-1.069546000	2.997649000
H	-4.464096000	0.035497000	0.735045000

Intermediate (3)

C	1.997669000	0.727391000	1.936464000
H	2.009983000	1.736029000	2.370768000
H	2.910899000	0.208191000	2.230561000
N	1.306000000	-0.271704000	-0.287997000
P	-0.649625000	0.106435000	2.189391000
C	1.905408000	-1.539246000	-0.147315000
O	2.618043000	-1.871316000	0.780468000
O	1.584786000	-2.327084000	-1.185514000

H	1.059816000	-0.089743000	-1.266809000
C	1.957851000	0.877307000	0.388673000
H	1.310491000	1.723812000	0.137801000
O	0.958189000	-0.021665000	2.569836000
C	3.378005000	1.194069000	-0.159057000
C	3.962715000	2.455219000	0.504844000
C	3.371121000	1.366142000	-1.687747000
H	4.025828000	0.343319000	0.092193000
H	4.119897000	2.334179000	1.581758000
H	4.934618000	2.698764000	0.060294000
H	3.305301000	3.322908000	0.353328000
H	3.045360000	0.462281000	-2.213402000
H	2.709117000	2.187933000	-1.993245000
H	4.379950000	1.603351000	-2.045482000
Cl	-1.055309000	-0.328387000	-2.504776000
C	2.071366000	-3.677666000	-1.120826000
H	1.712903000	-4.150665000	-2.035180000
H	3.164030000	-3.690959000	-1.079330000
H	1.668258000	-4.185701000	-0.240553000
H	-1.101719000	-0.735517000	3.235205000
H	-0.983875000	1.361823000	2.784663000
N	-1.190260000	1.839994000	-0.320959000
C	-1.366614000	2.923490000	-0.698143000
C	-1.624616000	4.254369000	-1.237694000
H	-0.797399000	4.934641000	-1.008823000
H	-1.736579000	4.189116000	-2.325541000
H	-2.545924000	4.667684000	-0.813178000
C	-1.542420000	-2.400648000	0.017580000
C	-3.266838000	-0.612620000	-0.037764000
C	-2.606522000	-1.690748000	0.624790000
H	-0.977702000	-3.097300000	0.634987000
H	-1.547074000	-2.610708000	-1.048374000
H	-3.411394000	-0.697005000	-1.114382000
Ru	-1.108530000	-0.224326000	0.039003000
C	-4.300697000	0.223288000	0.679667000
H	-5.308210000	-0.206402000	0.568203000
H	-4.089028000	0.300519000	1.754132000
H	-4.338828000	1.240247000	0.271992000
H	-2.775666000	-1.814002000	1.696128000

<u>Intermediate (4)</u>

C	-2.864595000	0.469717000	-1.683137000
H	-2.736011000	0.688860000	-2.751735000
H	-3.870117000	0.768257000	-1.385272000
N	-1.410998000	0.687192000	0.375837000
P	-1.490967000	-1.872235000	-1.515515000
C	-2.401101000	0.499608000	1.354333000
O	-3.593240000	0.424146000	1.134697000

O	-1.844980000	0.430842000	2.578268000
H	-0.526210000	1.018409000	0.787284000
C	-1.823689000	1.323791000	-0.896063000
H	-0.898148000	1.372034000	-1.478133000
O	-2.862065000	-0.946669000	-1.504428000
C	-2.347660000	2.776973000	-0.726838000
C	-2.649148000	3.422635000	-2.091390000
C	-1.353853000	3.649562000	0.055922000
H	-3.284375000	2.723373000	-0.157023000
H	-3.443650000	2.905291000	-2.638583000
H	-2.971281000	4.460974000	-1.955061000
H	-1.753977000	3.437758000	-2.727873000
H	-1.190111000	3.280998000	1.073847000
H	-0.378617000	3.693300000	-0.446552000
H	-1.731272000	4.675180000	0.137252000
C	-2.765164000	0.182949000	3.650527000
H	-2.155890000	0.170535000	4.554260000
H	-3.518553000	0.973507000	3.703426000
H	-3.266291000	-0.779047000	3.513019000
H	-2.167522000	-3.117439000	-1.459762000
H	-1.152292000	-1.895337000	-2.903136000
N	1.121640000	-0.163262000	-1.540963000
C	1.951719000	0.443177000	-2.079885000
C	3.038650000	1.203579000	-2.684442000
H	2.646274000	2.034589000	-3.279293000
H	3.675121000	1.599125000	-1.883562000
H	3.639374000	0.557996000	-3.333442000
C	-0.446713000	-2.362134000	1.751787000
C	1.376679000	-2.916285000	0.177955000
C	0.106869000	-3.208140000	0.764180000
H	-1.475168000	-2.532734000	2.062331000
H	0.187956000	-1.855206000	2.473764000
H	2.133301000	-2.509822000	0.847057000
Ru	0.041221000	-1.201458000	-0.060400000
C	1.921327000	-3.738973000	-0.965578000
H	2.491336000	-4.607303000	-0.602428000
H	1.117945000	-4.115931000	-1.610871000
H	2.598560000	-3.145793000	-1.590182000
H	-0.530715000	-3.954007000	0.287560000
O	1.177053000	0.096208000	1.300767000
C	2.321243000	0.729234000	1.099412000
C	2.366466000	2.149206000	1.120446000
C	3.554842000	0.062544000	0.873806000
C	3.555266000	2.848488000	0.923810000
H	1.440996000	2.686446000	1.315243000
C	4.738871000	0.770377000	0.674785000
H	3.566402000	-1.022534000	0.862405000
C	4.756902000	2.170349000	0.687943000
H	3.544244000	3.936622000	0.955725000

H	5.664073000	0.219747000	0.512581000
H	5.685323000	2.716179000	0.543486000

TS1

C	2.184729000	0.101152000	1.886485000
H	3.133570000	0.431925000	2.302933000
H	1.966905000	-0.897951000	2.276575000
N	1.002550000	-0.436266000	-0.302559000
P	-0.357376000	0.871017000	2.161226000
C	0.753760000	-1.853808000	0.008204000
O	0.178306000	-2.164989000	1.029610000
O	1.186633000	-2.633276000	-0.952740000
H	1.174708000	-0.372740000	-1.310229000
C	2.272780000	0.140577000	0.359309000
H	2.264617000	1.189163000	0.046861000
O	1.231408000	1.055578000	2.412877000
C	3.558227000	-0.532848000	-0.209369000
C	4.823946000	0.095853000	0.412439000
C	3.629874000	-0.409813000	-1.744727000
H	3.543076000	-1.598762000	0.057325000
H	4.950404000	-0.144364000	1.471872000
H	5.708089000	-0.288902000	-0.104309000
H	4.829359000	1.187478000	0.299969000
H	2.834876000	-0.949403000	-2.273030000
H	3.615786000	0.642264000	-2.058957000
H	4.570213000	-0.838604000	-2.103574000
C	-3.250467000	-0.851506000	-1.618345000
C	-2.968695000	-1.097519000	-0.166843000
H	-3.426220000	-1.767795000	-2.183243000
H	-4.053394000	-0.136336000	-1.797117000
Ru	-0.890707000	0.486329000	-0.008818000
C	-3.665708000	-0.487566000	0.831839000
Cl	-1.741340000	-0.087981000	-2.427701000
C	1.003388000	-4.082997000	-0.788632000
H	1.442166000	-4.515451000	-1.685011000
H	1.525946000	-4.410533000	0.110939000
H	-0.062297000	-4.305257000	-0.719414000
H	-0.798448000	-0.121888000	3.064337000
H	-0.828100000	2.056851000	2.754197000
H	-2.352900000	-1.966811000	0.076177000
N	-0.170055000	2.268099000	-0.573297000
C	0.114421000	3.331124000	-0.941813000
C	0.457496000	4.668242000	-1.407276000
H	1.299525000	5.064460000	-0.829149000
H	0.732771000	4.635089000	-2.467360000
H	-0.403938000	5.334599000	-1.284768000
C	-3.678298000	-0.908769000	2.265436000
H	-3.592397000	-0.052867000	2.946024000

H	-4.659805000	-1.355504000	2.482648000
H	-2.912653000	-1.654492000	2.498179000
H	-4.358310000	0.313710000	0.561389000
